# NMR Characterization of Information Flow and Allosteric Communities in the MAP Kinase p38γ

**DOI:** 10.1038/srep28655

**Published:** 2016-06-29

**Authors:** Phillip C. Aoto, Bryan T. Martin, Peter E. Wright

**Affiliations:** 1 Department of Integrative Structural and Computational Biology and Skaggs Institute for Chemical Biology, The Scripps Research Institute, 10550 North Torrey Pines Road, La Jolla, 92037 California, United States

## Abstract

The intramolecular network structure of a protein provides valuable insights into allosteric sites and communication pathways. However, a straightforward method to comprehensively map and characterize these pathways is not currently available. Here we present an approach to characterize intramolecular network structure using NMR chemical shift perturbations. We apply the method to the mitogen activated protein kinase (MAPK) p38γ. p38γ contains allosteric sites that are conserved among eukaryotic kinases as well as unique to the MAPK family. How these regulatory sites communicate with catalytic residues is not well understood. Using our method, we observe and characterize for the first time information flux between regulatory sites through a conserved kinase infrastructure. This network is accessed, reinforced, and broken in various states of p38γ, reflecting the functional state of the protein. We demonstrate that the approach detects critical junctions in the network corresponding to biologically significant allosteric sites and pathways.

Transfer of a signal from one site in a protein to another is a pervasive theme in protein regulation. Although our understanding of this process has advanced^1^,^2^,^3^,^4^,^5^ our ability to identify allosteric sites and the pathways between sites remains incomplete. This is largely due to difficulties in observing imperceptible conformational changes and associated altered dynamics that frequently accompany allosteric signaling^2^,^4^,^6^,^7^. Identifying allosteric sites is therapeutically desirable since these sites can provide greater specificity and potency compared to orthosteric sites, which are often spatially generic across functionally diverse proteins^8^,^9^. High throughput experimental^10^ and computational methods^11^,^12^,^13^ have recently been developed to identify allosteric binding sites. Characterizing the pathways of physically and dynamically coupled residues between these sites provides insight into the mechanism of allostery and can even lead to identification of novel allosteric sites^12^. The ability to identify signaling pathways is made difficult due to prerequisites of detailed structural and functional knowledge as well as limitations in longer timescales of simulated motions. We present an NMR based method to observe allosteric information flow independent of structural and functional knowledge. In contrast to NMR methods that identify a functional correlation^14^,^15^,^16^,^17^,^18^,^19^ the method presented here provides a description of the intrinsic relationship between conformationally and dynamically coupled residues.

NMR chemical shifts report on the time-averaged local chemical environment and therefore reflect conformation and dynamic processes on a broad timescale (ps-ms). Thus the chemical shift is ideally suited to detect forms of allostery that may be difficult or impossible to detect by other methods. Here we show how site-directed mutagenesis of methyl containing side chains can be used to mildly perturb the allosteric network at various locations throughout the protein. The effects of each of these perturbations propagate through allosteric networks and are measured as changes in ^1^H-^13^C methyl chemical shifts. Communication pathways are identified by mediation analysis of the chemical shift perturbations and are used in 2^nd^ order Markov model network analysis^20^ to characterize overlapping allosteric communities and flow between communities. Using a network analysis approach with chemical shift perturbations provides characterization of the network structure, reveals the overall flow of communication within that structure, and identifies critical nodes within the pathways.

In the present work, chemical shift network analysis was used to investigate the allosteric network in a eukaryotic kinase, p38γ MAPK. Eukaryotic kinases are a large family of phosphotransferases that regulate diverse cellular functions and yet share a common architectural core^21^ composed of an active site and an ATP binding site at the interface between the N-terminal and C-terminal lobes (N and C lobes). The spatially conserved core includes catalytic residues surrounding and contributing to contiguous networks of hydrophobic residues in “spines” that span across the two lobes^21^. The dynamic formation or breakage of the spines determines the activation state of the kinase^21^. It is likely that allosteric signals from core regulatory motifs, such as the activation loop, gatekeeper residue, DFG loop, catalytic loop, substrate binding site, and the hinge between lobes, are communicated through the completed spines. Experimental approaches have provided evidence for allostery between the conserved motifs in various kinases^22^,^23^,^24^,^25^ and molecular dynamic simulations have suggested correlated motions across the spines and regulatory motifs in Protein kinase A (PKA) and c-Src kinase^26^,^27^,^28^. However, the nature of the communication between conserved elements is yet to be completely characterized.

By network analysis of chemical shift perturbations caused by methyl mutagenesis of p38γ we observe the pathways of communication between the various regulatory elements of p38γ and how they respond to changes in catalytic state. The network structure reveals how these elements are connected to each other and how they communicate. In the inactive apo, ATP bound, inhibitor bound (DFG-out), and phosphorylated forms of p38γ we find that the structure of the allosteric network reflects the conserved kinase infrastructure, including distinction between N and C lobes, hydrophobic spines, as well as MAPK specific regulatory elements. The flow of information revealed by the analysis identifies novel mechanisms for allosteric effects in docking site interactions, auto-activation, and DFG-out inhibition. Critical nodes in these networks - those that have the highest number of connections and greatest amount of communication flowing through them - are found to correspond very well to known allosteric sites. We characterize the pathways connecting these regulatory nodes to the active site. The results presented demonstrate the method’s effectiveness to characterize network structure and identify allosteric sites by improving our understanding of the allosteric pathways within p38γ.

## Results

### Identification of long-range perturbations

**Figure 1:**
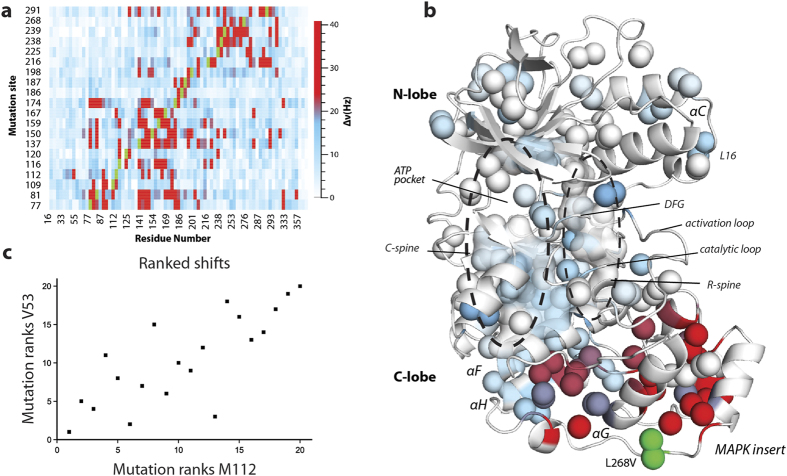
Long-range methyl chemical shift perturbations. (**a**) Heat-map of ^1^H-^13^C chemical shift perturbations (in Hz) for methyl resonances in p38γ (mutation-wildtype) caused by minimally disruptive mutations. Green represents mutation site. (**b**) ^1^H-^13^C chemical shift perturbations due to the substitution L268V in the MAPK insert mapped onto a homology model of the inactive apo p38γ structure. Colors correspond to shift differences in Hz, using the same scale in (**a**). (**c**) The ranks of each mutation induced chemical shift perturbation for V53 and M112. Each point corresponds to a mutant as ranked among the set of mutations for the specified residue.

A set of 20 conservative methyl mutants was used in conjunction with triple resonance NMR experiments to assign 73% of methyl resonances for Ile(δ1), Leu(δ1/δ2), Val(γ1/γ2), and Met(ε) in p38γ (see Methods and Supplementary text). The residues for which methyls have been assigned are well distributed throughout the protein, including all regulatory elements. The single-site mutations used for assignments were found to cause long-range perturbations, at distances up to 40 Å or greater, in the local environment of methyl groups (Fig. 1a). Long range chemical shift perturbations caused by binding of substrate, inhibitor, and nucleotide to kinases have been observed previously, but have been described only in qualitative terms^23^,^24^,^29^,^30^. By using conservative mutagenesis, all intrinsic pathways present in a single state of p38γ are probed. In addition, we considered the full range of chemical shift perturbations down to very small differences, ~1 Hz, revealing subtle and underappreciated redistribution of sampled states in the conformational ensemble.

The most obvious pathways for propagation of chemical shift changes follow known conserved hydrophobic spines. For example, mutations such as L268V in the MAPK insert, exhibit chemical shift perturbations that extend from the site of mutation to the αF-helix and residues within and surrounding the ‘regulatory’ spine (R-spine), a distance of >40 Å (Fig. 1b). This supports the hydrophobic spine model of allosteric communication and agrees well with statistical sequence comparison that implicates a linkage between the MAPK insert and active site^31^.

To characterize the communication pathways along the spines and other previously unknown routes, rank correlation coefficients between methyl groups were determined across the set of mutation-induced chemical shift perturbations. For example, the rank correlation between M112 and V53 is shown in Fig. 1c, wherein mutations were given integer ranks based on the magnitude of induced chemical shift perturbations for each methyl resonance. We ranked perturbations in order to 1) allow identification of monotonic relationships, 2) reduce the effect of errors in chemical shift measurement, and 3) allow inclusion of perturbations near mutation sites (see Supplementary text). In addition, the mutations are distributed throughout the protein (Supplementary Fig. S2) so that analysis of the chemical shift changes should reflect an unbiased view of the network. As expected, we found correlations of perturbations between residues not only local to one another but distant as well. This led us to investigate the communication network reflected by these correlations.

### A network of overlapping communities

**Figure 2:**
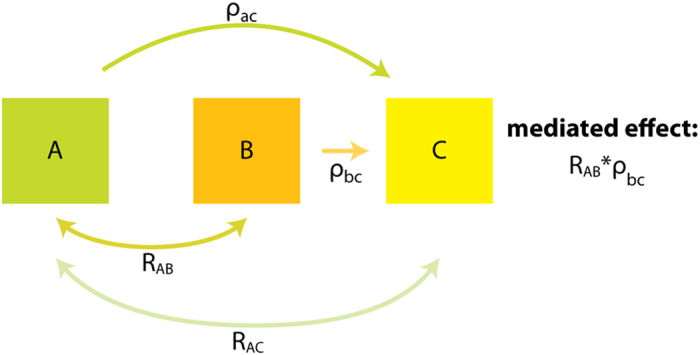
Mediation analysis. Effects of residue A on C are mediated by B if 1) A affects mediator or residue B (2 point correlation R_AB_); 2) B affects C (multiple regression coefficient ρ_bc_); 3) Positive correlations; 4) Indirect effect (ρ_ac_ < R_AC_). If these criteria are met then flow travels from A to B to C with the mediated effect of R_AB_*ρ_bc_.

Uncovering the underlying communication network of a system reveals its inherent structure and interdependency, which can be as valuable in understanding the function of that system as its tertiary structure. Since multiple communication pathways have been proposed to connect regulatory elements to effector sites within proteins^32^, including MAPKs^25^, and since a residue may be involved in more than one pathway, it is intuitive to consider allosteric networks as overlapping communities of interacting residues. There are several network analysis methods to characterize overlapping communities that provide information on the network hierarchy^33^,^34^, as well as the behavior within the network^20^. Since we are interested in how the communities of correlated residues interact with one another within the context of the intramolecular communication network, we chose to use the Mapequation method^20^. This method, which optimizes the minimum length of a random walk on the network, identifies communities as patterns of flow between residues and, among popular methods, provides one of the most accurate descriptions of networks^35^. This treatment is ideal for analysis of the p38γ data since each mutation causes chemical shift perturbations that flow in a spatially directed manner from the site of mutation. To convert chemical shift perturbation pathways into quantifiable flow we utilized the correlations of perturbations across the entire set of mutations, where a stronger correlation between residues corresponds to stronger flow of information between those residues. Mediated effects of these perturbations were analyzed in order to identify and quantify directed flow along 3-residue paths (Fig. 2). These 3-residue paths provide insight into how the flow of chemical shift perturbation depends on where it originated, in other words the memory effects of the network. The mediation analysis identified 875 pairwise correlations and 1304 sets of 3-residue flow in inactive p38γ (Supplementary Table S8). In the Mapequation analysis, random walks on the network were weighted by the directed flow for every 3-residue path in order to model the spread of perturbations across the protein. The use of memory in the Mapequation has been shown to produce a richer and more accurate network description than analysis without memory effects, which utilizes pairwise flow^20^.

### Network structure of inactive apo p38γ

**Figure 3:**
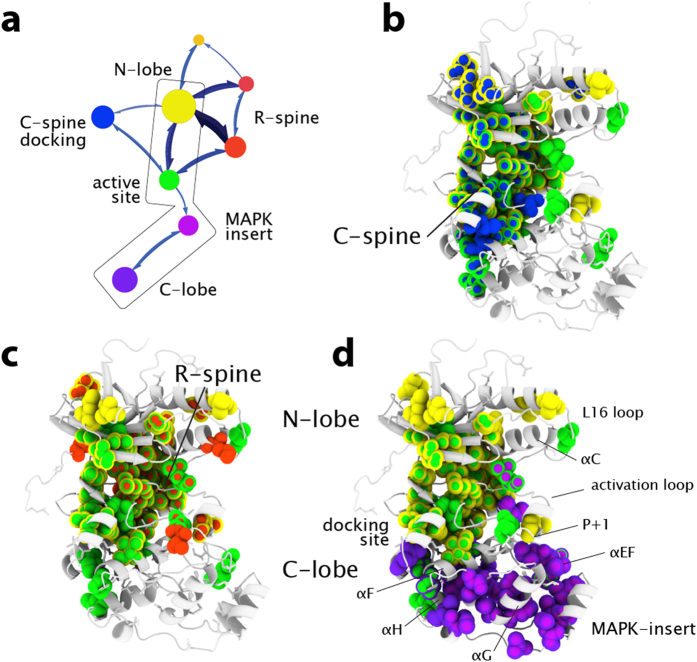
Network structure and flow of inactive apo p38γ. The method identified 15 communities with membership greater than 5 residues in inactive p38γ, but only the largest 8 communities are shown for clarity. Identified communities from chemical shift perturbation networks are colored based on tertiary structure and regulatory element: N-lobe, yellow; C-lobe, purple; active-site, green; C-spine, blue; R-spine, red; MAPK-insert, lavender. Modules depicted with similar shades of color overlap the same structural element. (**a**) Network map of inactive p38γ. The size of the modules represents the amount of flux and connections between residues within the module. Thickness of arrows between communities represents the amount of flux between communities. The C-spine (**b**) and R-spine (**c**) and connected communities are mapped on the homology model of inactive apo p38γ. Residues in multiple communities are depicted as multicolor spheres. The boxed region of the network map (panel a) is mapped on the structure in (**d**), illustrating the communities and flow linking C-lobe and N-lobe.

To ensure that the novel data handling used in our method did not adversely impact the networking results, we compared the results with those obtained using simpler pairwise ranked correlations. We found that the clustering results from the Mapequation network analysis agrees well with results from two point correlations using the link community method^34^ (Supplementary Fig. S3) and agglomerative clustering (Supplementary Fig. S4). For all of these methods, despite the fact that no structural information was used, the communities identified from chemical shift perturbations correspond extremely well to structural features of p38γ (Fig. 3, see Supplementary Table S9 for community membership). Communities can be conceptualized as clusters of residues that react similarly to each mutation and have significant communication with one another. The modules with the highest amount of internal communication from the Mapequation analysis, represented by module diameter in Fig. 3a, correspond to the N-lobe (yellow) and C-lobe (purple) of p38γ. Although the network structure nicely reflects the tertiary structure, it also identifies communities that span various structural elements within the architectural core, demonstrating that communication networks cannot be readily inferred by protein structure alone. The two major communities in inactive apo p38γ are connected to each other by two overlapping communities composed of 1) the active site, at the interface between lobes, and docking site (green) and 2) the MAPK-insert and GHI subdomain in the C-lobe (light purple) (Fig. 3a box, Fig. 3d). The GHI subdomain, composed of the α-helices G, H, and I, is a conserved feature of eukaryotic kinases that has been proposed to integrate signals originating in the C-lobe with the active-site^21^,^26^. Thus the communities of chemical shift perturbation reflect the conserved and MAPK-specific infrastructure.

### Network flow reveals relationship between communities

The network analysis of inactive apo p38γ also reveals the degree of information flow, which corresponds to the propagation of chemical shift perturbations, between communities of related residues. This novel nuanced understanding of the p38γ communication network is represented on the map in Fig. 3a as the thickness of the arrows connecting communities. Mapping flow reveals that the active site community has stronger connections to the N-lobe than to the C-lobe and MAPK-insert, therefore discounting a role of the C-lobe in the regulation of inactive apo p38γ. Interestingly, a community that corresponds well to the spatially conserved ‘catalytic spine’ (C-spine) is detected and displays weak flow to the rest of the protein (Figs. 3a,b; blue). The weak information flow between the C-spine and other parts of the protein likely reflects the lack of bound ATP. ATP binding has been proposed to complete the C-spine and prime the active kinase for activity by stabilizing compaction of the N and C lobes^21^. The compaction of the lobes concomitant with ATP binding will likely increase the C-spine module’s connections and flow to other communities in the protein.

In contrast, a community that corresponds closely to the spatially conserved hydrophobic R-spine (Fig. 3a,c; red) is strongly connected to the rest of the protein, reflected by high information flow to other communities. This suggests, unexpectedly, that the R-spine is at least partially preformed and transmits information within inactive p38γ. A preformed R-spine is unexpected in an inactive kinase, since the hydrophobic packing of the R-spine typically indicates that the active-site residues are preorganized for catalysis^21^. However, it is possible that the R-spine residues form a dynamic contiguous network yet are not properly organized for phospho-transferase activity. The physically connected but likely ill-formed spine in p38γ may have evolved to allow allosteric signal propagation along the conserved architecture while remaining inactive. Our results offer a broadened understanding of hydrophobic spines, outside of their fundamental role in facilitating the active state: these pathways dynamically pre-exist in the inactive state and enable allosteric communication between lobes.

### ATP binding to inactive p38γ reconfigures the network structure

**Figure 4:**
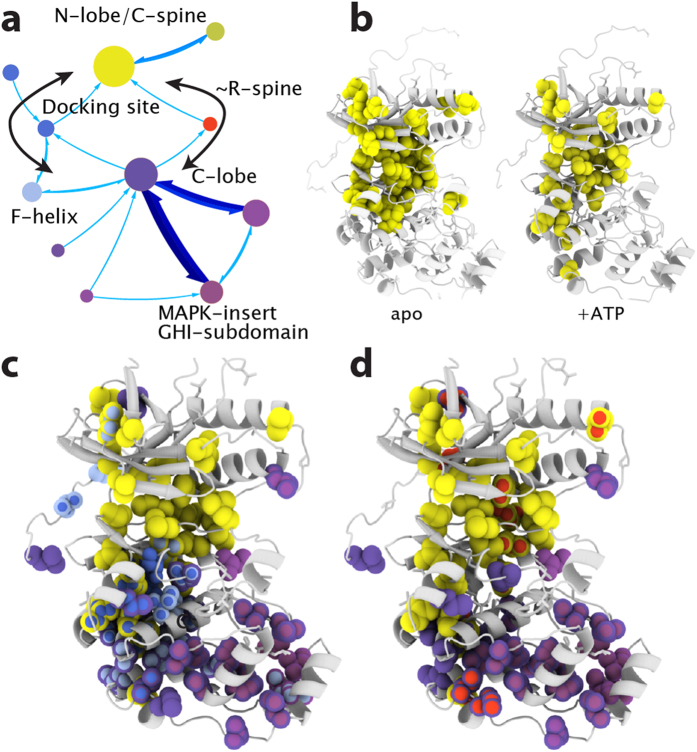
Response of p38γ network to ATP binding. (**a**) Network structure of ATP + p38γ, communities are colored based on tertiary structure and regulatory element: N-lobe/C-spine, yellow; C-lobe, purple; docking site, blue; R-spine, red; MAPK-insert, lavender. (**b**) Comparison of N-lobe communities in inactive apo and ATP bound p38γ. Completion of the C-spine by ATP extends the N-lobe community further into the C-lobe. (**c**) Communities representing a pathway (left black arrow in (**a**)) between C-lobe (purple) and N-lobe (yellow) through the docking site (blue) and C-spine (yellow). The pathway through the C-spine is not present in apo inactive p38γ. (**d**) Communities involved in the R-spine (red) pathway (right black arrow in a) between N (yellow) and C (purple) lobes.

To investigate how completion of the C-spine affects the network, we used the same mutagenesis strategy to produce 13 mutants of p38γ in complex with ATP. These mutants were chosen quasi-randomly, to ensure that mutations sampled the entire protein. Simulations using chemical shift data for the apo enzyme, for which 20 mutations are available, show that data obtained from as few as 11 mutations are sufficient to define the network structure, demonstrating the robustness of the method (Supplementary text and Fig. S5). Mediation analysis was performed on the chemical shift changes caused by the 13 mutations in the ATP complex and 719 significant pairwise correlations and 1066 three amino acid paths were identified (Supplementary Table S10). Although the adenine ring of the bound ATP completes the p38γ C-spine, the unphosphorylated kinase is catalytically inert. The network analysis reveals that, in the presence of bound ATP, the C-spine’s connections are strengthened to the extent that the C-spine becomes incorporated into the N-lobe community (Fig. 4a,b, see Supplementary Table S11 for community membership). This is illustrated by the expansion of the N-lobe community further into the C-lobe, in comparison to the apo state network (compare Figs 4a and 3a). In addition, the relative flow into and out of the R-spine is decreased compared to the inactive apo state. This may reflect the improper compaction of the R-spine caused by ATP binding^30^,^36^, which likely reduces aberrant hydrolysis of ATP by inactive p38γ. The completion of the C-spine by ATP reconfigures p38γ’s network by creating two discrete pathways between the N and C-lobes via 1) methyls surrounding the R-spine and 2) through methyls in the αF-helix, docking-site, and C-spine (Fig. 4a,c,d).

### Activation reinforces existing pathways

**Figure 5:**
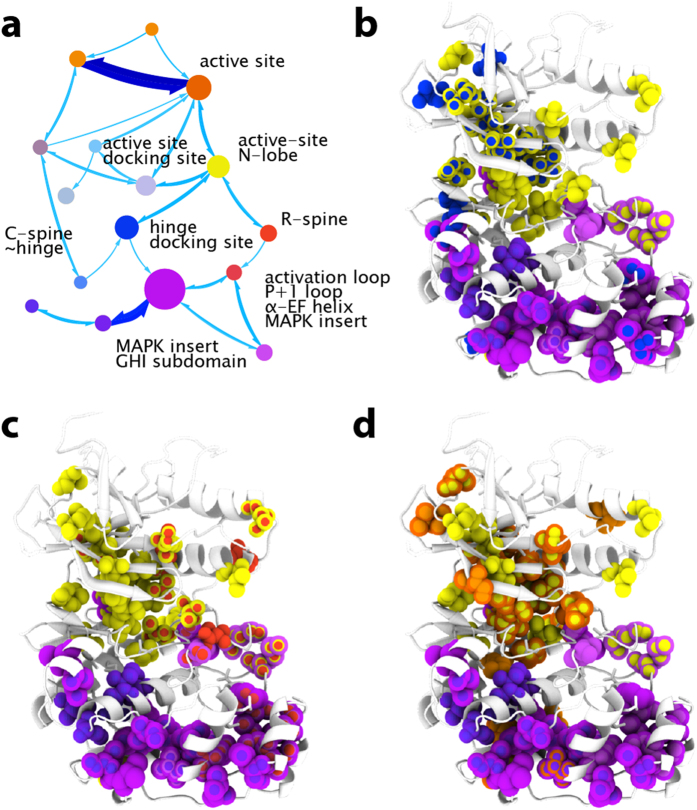
Network response to activation. Network analysis identified 16 communities with membership greater than 5 residues in active p38γ. Communities are represented in shades of color based on tertiary structure and regulatory element: N-lobe/active-site, yellow; C-lobe/MAPK-insert/GHI subdomain, purple; active-site/GHI subdomain, orange; C-spine/hinge, blue; R-spine, red. The network map is depicted in (**a**) with flow and membership represented as in Fig. 3. Flux through communities connecting N and C-lobes are depicted: through the C-spine hinge (**b**), through the R-spine (**c**), and through the active site residues (**d**).

Activation of MAPKs involves dual phosphorylation of the activation loop, which leads to salt bridge formation between the phosphorylated residues and the N and C-lobes, and causes the lobes to close towards each other accompanied by a concerted rearrangement of secondary structural elements around the active site. This compaction and reorganization aligns the catalytic residues for activity and completes the R-spine^21^. Since our chemical shift network analysis finds evidence for the pre-existence of the hydrophobic R-spine pathway in the inactive apo state of p38γ, we were motivated to investigate how this network responds to activation. The same set of 13 conservative p38γ mutants used in the analysis of the ATP-bound state were dual phosphorylated by MKK6 and chemical shift perturbations due to the mutations were used to analyze the network structure. 625 significant pairwise correlations and 460 sets of 3-residue pathways were identified in active p38γ (Supplementary Table S12). Chemical shift based network analysis reveals that upon activation, the R-spine community, as expected, forms more extensive connections between the N and C-lobes (Fig. 5a,c; red, see Supplementary Table S13 for community membership) than in the inactive state, which only displays connections from the R-spine to the N-lobe. The residues in this hydrophobic spine have membership in multiple overlapping communities, including those containing the ATP binding site, active site, GHI subdomain, and MAPK insert. This suggests the R-spine is properly formed by activation and makes more extensive connections with the rest of the protein when in the activated state.

The N and C-lobe compaction is also reflected in an extension of a pathway between lobes, through the docking site (Fig. 5a,b; blue). This highlights the importance of the docking site, which has been observed to allosterically enhance substrate binding, nucleotide binding, and the catalytic rate of active p38α^30^,^37^. Concomitant with activation, the regulatory GHI subdomain^21^,^26^ becomes a member of the active site community (Fig. 5a,d; orange), which is also the community with the second highest amount of internal communication. Together, this suggests that the GHI subdomain has more influence on the active site in the activated state of p38γ than in the inactivated state. A comparison of the network maps of inactive and activated apo p38γ reveals that activation reduces overall flow across the network but extends existing communication pathways involved in regulation (Figs 3a and 5a). It is likely that binding of substrate and nucleotide, which is accompanied by further conformational rearrangements that lead towards catalytic competence^26^,^30^, will further enhance flow over the pathways.

### BIRB796 binding to inactive p38 causes network fragmentation

**Figure 6:**
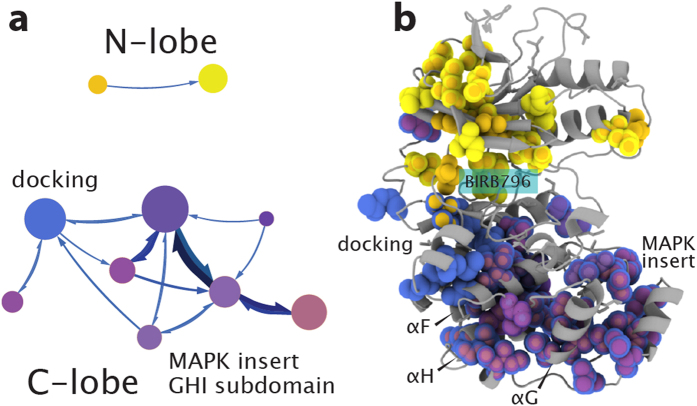
Network disruption by BIRB-796. (**a**) The method identified 17 communities with membership greater than 5 residues in DFG-out p38γ, of which only 10 are shown for clarity. Communities are represented in shades of color based on tertiary structure and regulatory element: N-lobe, yellow; C-lobe, purple; docking site, blue. Communities in the C-lobe (purple) are heavily overlapped and include the MAPK insert, portions of the GHI subdomain, and F-helix. Darker shades of purple indicate communities that include the F-helix, lighter shades of purple indicate communities that include the H-helix. (**b**) Communities are mapped on the DFG-out p38γ homology model, illustrating the disruption in communication between N and C-lobes. The docking site has become insular, represented by weak flow to other communities and membership only in the C-lobe.

Kinases can also be artificially modulated towards dysfunctional states. In particular, DFG-out inhibitors bind in the active site of kinases and disrupt the DFG-motif that constitutes a part of the R-spine^38^,^39^,^40^. This disruption impedes catalysis and alters the dynamics and conformation of the activation loop to prevent phosphorylation by upstream kinases. To gain insight into how the communication network in p38γ behaves following breakage of the conserved infrastructure, we bound the DFG-out inhibitor BIRB796 to the inactive kinase and performed network analysis using the chemical shift perturbations associated with 12 methyl mutations. 589 significant pairwise correlations and 500 sets of three residue paths were identified (Supplementary Table S14). The resulting community map reveals that the DFG-out inhibitor severely reduces connection and flow between the N and C-lobes (Fig. 6a,b, see Supplementary Table S15 for community membership). The disruption of the R-spine is reflected in the network by the loss of communities spanning across the active site. This includes the loss of the R-spine community as well as the loss of the active-site community, which acts as an interface between the N and C-lobes in the inactive apo state. Within the C-lobe of the inhibitor-bound complex, regulatory elements are identified in separate overlapping communities. Information flow between the module containing the docking-site and the overlapping module containing only the MAPK insert and GHI subdomain is relatively weak, indicating that the docking site is connected with the rest of the C-lobe but is fairly isolated in terms of communication (Fig. 6a,b). The loss of communication between the docking site and C-lobe, as well as the loss of communication between N- and C-lobes, likely contributes to the inability of upstream kinases to phosphorylate BIRB796-bound p38, since docking site interactions will no longer cause the allosteric conformational change in the activation loop required for phosphorylation^25^.

### Identification of critical nodes and pathways in allostery

The network structure maps described above provide novel insight into pathways and nodes in allosteric communication. For example, in inactive apo p38γ, the N-lobe community not only has high internal communication between residues within the community (module diameter) but also has more connections to other modules, four, than does the C-lobe community, which connects to only one module, suggesting that the N-lobe has an important role in inactive apo p38γ (Fig. 3a). Inactive p38γ complexed with ATP exhibits increased flow between the C-lobe community and overlapping communities consisting of the MAPK-insert and GHI subdomain (Fig. 4a,c,d). Activation also causes an inversion of the major community membership and communication networks, where more internal connectivity and flow is now present in the C-lobe (Fig. 5a) compared to the inactive state (Fig. 3a). This suggests regulatory importance of C-lobe elements, such as the MAPK insert and GHI subdomain, in the compact conformation of the activated and ATP-bound states. A more direct approach to identifying critical residues and regions is to consider residues that have the highest number of network connections. The changes in the localization of residues with the highest number of connections highlight the reorganization of the allosteric network in the different states of p38γ as well as the importance of regulatory elements in various states (Supplementary text, Supplementary Fig. S6).

### Network flow identifies critical pathways

**Figure 7:**
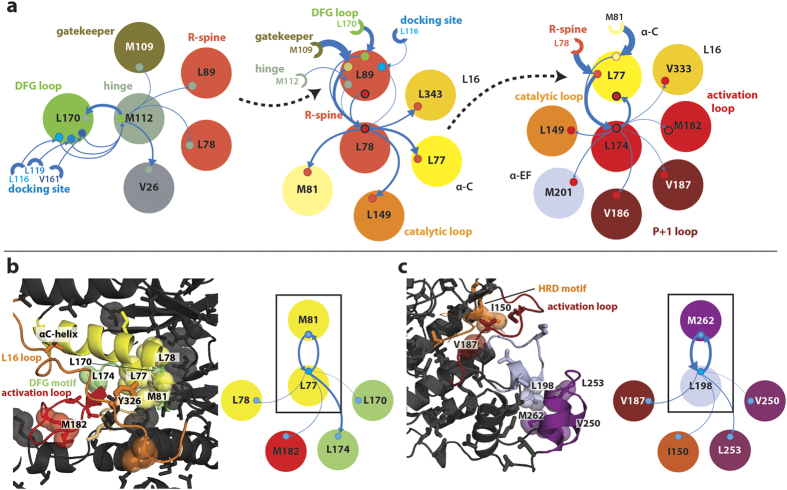
Critical network nodes reveal regulatory sites and pathways. (**a**) A series of highly ranked memory nodes in inactive p38γ reveal multiple pathways that extend greater than 20 Å from the docking site to the activation loop. Large circles represent residues with a direct connection to the memory node of interest and small circles represent connected memory nodes. Semi-circles depict residues with an indirect connection to the memory node. The relative amount of flow between residues is indicated by the thickness of solid arrows. Dashed arrows indicate the common residue belonging to the next memory node in the path. Colors correspond to regulatory elements, as labeled. Memory nodes (left to right) include M109-M112, L89-L78, and L77-L174. (**b**) A significant memory node in the αC-helix of apo p38γ suggests a route for auto-activation by Y326 phosphorylation. (**c**) A highly ranked memory node in the MAPK insert of activated p38γ reveals a communication pathway to catalytic residues.

Examination of residues with high connectivity and high information flow identifies undiscovered critical pathways. We ranked the magnitude of flow of chemical shift perturbations across all pairs of correlated residues (memory nodes) to and from other memory nodes. Flow is explicitly attributable to a series of 3-residue paths over each memory node. Thus we can identify critical memory nodes, in terms of flow, and the communication pathways of that flow. In the inactive apo form of p38γ, a series of highly ranked memory nodes form pathways from the docking site to the activation loop and active site residues, over a distance of >20 Å (Fig. 7a). A highly ranked memory node representing flow through L170, within the DFG loop, to M112, a hinge residue, exhibits incoming flow from docking site residues L116, L119, and V161 (Fig. 7a-left). Information is transferred across the L170-M112 memory node to the so-called ‘gatekeeper’ residue (M109) and the N-terminal portion of the conserved R-spine (L89 and L78). The gatekeeper residue, which is between hydrophobic spines and has been proposed to interface with the R-spine to constrain DFG loop and activation loop flexibility^41^, also has connections to the R-spine memory node (L89 –L78) (Fig. 7a-middle). The R-spine memory node takes additional input from the DFG loop (L170) and directly from the docking site (L116) with flow continuing to the catalytic loop (L149), the L16 loop (L343), and the αC-helix (L77). Finally, information flow across the memory node L77-L174, which involves the αC-helix (L77) and the DFG motif/activation loop (L174), spreads further into the activation loop (M182) and into the P + 1 loop (V186, V187) (Fig. 7a-right). Thus the highly ranked memory nodes and their communication pathways reveal a novel mechanism for the allosteric communication related to activation between docking site, activation loop, and P + 1 loop. In addition, these pathways may be involved in docking site mediated auto-activation (see Supplementary text).

In apo forms of p38γ, the significance of the αC-helix is highlighted by the M81-L77 highly ranked memory node. The memory node displays flow from the αC-helix (L77, M81) to active site residues (Fig. 7b). Communication is observed across the M81-L77 memory node to the DFG motif (L170), αC-helix/R spine (L78), and activation loop (M182). Significantly, the side chain of L77 makes contact with the side chain of Y326 in the L16 loop in crystal structures of p38. The phosphorylation of Y326 in p38α/β in T-cells induces activation of p38 via auto-phosphorylation of T183 in the activation loop^42^,^43^. Thus, changes in interaction with the M81-L77 memory node following Y326 phosphorylation may be communicated by the pathways detected in our network analysis and stabilize an active conformation of the R-spine and DFG loop.

A final example of the network structure providing a mechanistic understanding of allosteric pathways is given by a memory node in the MAPK-insert of activated p38γ (Fig. 7c). The M262-L198 memory node, representing flow from M262 in the MAPK-insert to L198 in the αEF-helix, reveals information flow to the P + 1/activation loop (V187) and HRD motif (I150). The HRD motif includes the catalytic base (D153). Thus, the flow over this memory node suggests that changes in interaction between L198 and M262 in the MAPK insert leads to perturbation of the P + 1 loop, activation loop, and catalytic loop, direct participants in p38γ activity. Interestingly, this memory node is only highly ranked in the network analysis of the activated state and is not present in the inactive states. It is possible that the formation of this pathway is concomitant with stabilization of the active state. Indeed, lipid molecules have been found to bind in the hydrophobic groove formed between the MAPK-insert and αEF-helix, between L198 and M262, in the inactive form of p38α leading to auto-activation^44^.

## Discussion

We have demonstrated the utility of a simple memory network analysis of NMR chemical shift perturbations caused by conservative mutagenesis. Mutations of methyl-containing residues are commonly made to obtain methyl chemical shift assignments and thus these constructs can serve dual purpose. The method provides a highly detailed map of overlapping communities in allosteric networks, corresponding information flow, and ranks of residues in relation to the network. The overlapping communities found in p38γ correlate well with the 3D structure, despite no structural information being used as input. The analysis also confirms an underlying conserved kinase infrastructure, originally identified by local spatial alignment^21^, as well as the MAPK specific infrastructure, including the MAPK insert and L16 loop.

Consideration of the network structure of a protein and its flow provides insight into important structural regions and pathways of communication. Previous application of graph theory to computational simulations to identify the topology of non-overlapping communities of correlated motions has demonstrated the utility of revealing the network structure of a protein^26^,^45^,^46^. In particular, *in silico* network analysis of PKA by McClendon *et al*. showed that functionally significant structural features are partitioned into non-overlapping communities, effectively acting as a hub for these communities^26^. Similarly, our network analysis of p38γ finds that key regulatory elements are contained entirely in their own communities and that functional significance is indicated by a high degree of overlap and flow with other communities. For instance, communities that overlap a large number of neighboring communities likely act as nodes in communication. In addition high flux of information identifies communities that share relatively large amounts of information, again suggesting functional relevance. One example of this is the active site community, including docking site residues and the interface between N and C lobes, which has high overlap and high flux with neighboring communities in inactive p38γ. In the activated form of p38γ, the R-spine module is highly overlapped with the N-lobe, C-lobe, and MAPK insert but lacks high information flux traveling through it. The flow in and out of this community will likely increase upon ATP or substrate binding as the compact state becomes stabilized^30^,^36^. Likewise, the C-spine/N-lobe community in the ATP bound inactive form of p38γ is highly overlapped with other communities but lacks high flux. Again, activation is likely to increase the flow through this spine. Thus the network structure and flow not only reflects important regulatory regions but also reveals the functional state of the system.

Finally, one of the more powerful aspects of the network analysis is the ability to identify critical network nodes and pathways that correspond to biologically relevant allosteric sites in the enzyme. We have demonstrated that our method correctly identifies previously known regions of regulatory function and also provides new understanding of the allosteric communication pathways between these regions, providing valuable mechanistic insights into allosteric pathways that can guide further experiments. In p38γ, for example, residues within key pathways could be mutated and the effect on cooperativity of docking peptide binding, activation, or catalysis could be characterized. In addition, solvent accessible pockets that are strongly connected to critical nodes and pathways may be more promising candidates for targeting by small molecule compounds than pockets that are only weakly connected to the network^8^,^9^. The network analysis method of chemical shift perturbations provides an information-rich and direct view of allosteric networks of proteins in solution.

## Methods

### Minimally perturbing mutagenesis

Isotopically labeled His_6_ N-terminally tagged human MAPK p38γ was overexpressed in a PET15b (Invitrogen) plasmid in *E. coli* Rosetta plysS BL21(DE3) (Invitrogen) cells at 288 K. Site-directed mutagenesis was performed by Quikchange (Agilent) to generate the following conservative mutations: L77V, M81L, M109L, M112L, L116V, M120L, M137L, I150L, L159V, L167V, L174V, V186A, V187A, L198V, M216L, L225V, I238L, M239L, L268V, and M291L. Methyl mutants were expressed in cells grown in 50 ml of M9 minimal media containing ^13^C glucose as carbon source. Purification procedures were adapted from^47^. Briefly, cells were lysed by sonication in 50 mM Tris pH 8.0, 500 mM NaCl, 10 mM β-mercaptoethanol, 0.1% Triton X-100, and 1 mM PMSF and p38γ was purified by Ni-NTA affinity chromatography. Purified protein was exchanged into NMR buffer containing 25 mM deuterated Hepes pH 7.2, 250 mM NaCl, 1 mM TCEP, and 0.01% w/v NaN_3_ in 100% D_2_O. Final protein concentrations were between 100–400 μM. Sets of mutants were expressed simultaneously with a wildtype sample and purified identically with the same buffer stock.

### Activation and ligand binding

A constitutively active mutant of GST-tagged MKK6 (S207D, T211D) was overexpressed in Rosetta plysS BL21(DE3) (Invitrogen) *E. coli* cells at 293 K in LB media. Cells were lysed by sonication in 20 mM Tris pH 8.0, 100 mM NaCl, 5 mM DTT, 0.1% Triton X-100, 1 mM EDTA, and 10% glycerol. GST-MKK6 was purified by GST-Sepharose 4b (GE) affinity chromatography. Protein was dialyzed into 20 mM Hepes pH 7.6, 100 mM NaCl, and 2 mM DTT. p38γ was activated (dual phosphorylation on Thr183/Tyr185) by incubation with the mutant MKK6 at room temperature in 75 mM Hepes pH 7.5, 0.1 mM PMSF, 1 mM NaF, 0.1 mM NaVO_4_, 0.1 mM β-glycerol phosphate, 10 mM MgCl_2_, and 1 mM DTT. Completion of phosphorylation was monitored by a phosphorylation-dependent change in elution time over analytical HPLC (C4 reverse-phase) and by change in ^1^H-^13^C chemical shift of p38γ . Following activation of p38γ, MKK6 and ATP were removed by Ni-NTA affinity chromatography, as described above, with an added 10x column volume wash of column buffer with 1 M NaCl and additional dialysis against 25 mM Hepes pH 7.2, 250 mM NaCl, 5 mM DTT, and 1 mM EDTA. Doramapimod (BIRB796) from Selleck Chemicals was resuspended in deuterated DMSO and incubated at room temperature with purified inactive p38γ at 1:2 molar excess. The pH of the sample was readjusted, as necessary, to pH 7.2 and centrifuged to remove insoluble BIRB796. ATP and MgCl_2_ were prepared in NMR buffer and added in molar excess (1:50) to inactive p38γ. Complete binding of BIRB796 or ATP to p38γ was monitored by ^1^H-^13^C chemical shift.

### NMR spectroscopy

^1^H-^13^C HMQC NMR experiments were performed in NMR buffer at 293 K on Bruker Avance 750 MHz or 900 MHz spectrometers equipped with triple resonance gradient probes. Data were acquired using optimized Poisson-gap non-uniform sampling (NUS)^48^ in the indirect dimension. This allowed a higher number of transients to be collected over a given time for greater signal to noise. 40–100% of 512 real and imaginary points in the ^13^C dimension were collected over a spectral width of 40 ppm. Spectra of the mutants were acquired together with an identically prepared wildtype sample and with identical NMR experimental parameters (% NUS, number of increments, spectral width). Data were reconstructed and processed with compressed sensing in MDDNMR and NMRPipe^49^,^50^,^51^. Spectra were analyzed with NMRView5^52^. ^1^H-^13^C combined chemical shift differences were calculated in Hz at 750 MHz using the equation Δ*v* = ((Δ^1^H*750)^2^ + (Δ^13^C*188.8)^2^)^1/2^^53^.

### Network analysis

Three residue pathways of information flow were identified by mediation analysis of correlated chemical shifts over the entire set of mutations. A modified method of Baron & Kenny^54^ as described in^55^ was used to identify mediated pathways (Fig. 2). In brief, residues A, B, and C form a ‘pathway’ of A to B to C if B mediates the effect of A on C. We simplify analysis by only considering positive correlations (Supplementary text). The tests used to identify mediation through B are: 1) A affects B, i.e. the two-point correlation between residues A and B (R_AB_) is significant; 2) B affects C when considering multiple regression of A, B, and C, i.e. the partial correlation between residues B and C (ρ_bc_) is significant; 3) there is an indirect effect, i.e. the partial correlation between A and C (direct effect, ρ_ac_) is less than the two point correlation between A and C (R_AC_). The mediated effect is then quantitated by R_AB_*ρ_bc_ (Fig. 2). Uncorrected p-values from t-tests of two-point correlations and multiple-regressions were used to determine significance. In the inactive apo p38γ dataset, correlations and partial correlations were considered significant if p < 0.02, for the inactive ATP complex p < 0.03, for the inactive BIRB796 complex p < 0.03, and for the active apo p38γ p < 0.04. The p-value cutoffs were chosen for enrichment in positive correlations (Supplementary Fig. S8). In order to minimize the effects of errors due to p-value cutoff and to create a continuum of information flow from 0 (no flow) to 1 (high flow), soft-thresholding was utilized^56^. This involves choosing a soft-threshold limit using the approximate scale-free topology criterion of 57 and raising correlation coefficients by the chosen soft-thresholding power. For all data, a soft-threshold of 8 was applied to the two-point correlation coefficients. The mediated or indirect effects were used as the weights in a random walk over the network and the two-point correlation coefficients were used as teleportation probabilities (jumps between nodes) in the MapEquation software with 2^nd^ order dynamics^20^,^58^. The perturbations for a common set of 78 methyl containing residues were included in the analysis of various states of p38γ. Other assigned methyls in the inactive apo state were discarded due to ambiguity in assignment in either ATP bound, BIRB796 bound, and activated states. Under these conditions we found that at least 11 mutants were required to reliably reproduce the network structure reported (Supplementary text). For each state of p38γ, 20,000,000 runs in the MapEquation software were performed and the shortest walk length was chosen. All statistical analyses were conducted in the R software environment^59^ utilizing various packages^57^,^60^,^61^,^62^. Python scripts for preparation of chemical shift data for R input, R scripts, and input for MapEquation are available for download at http://www.scripps.edu/wright. Homology models for p38γ were generated with the I-TASSER structure prediction method^63^. In brief, the inactive apo and BIRB796 bound models were predicted by I-TASSER using threading structures (p38α) with 62 to 67% sequence identity to aligned regions of p38γ. In addition activated structures of p38 were manually excluded as templates. The model for BIRB796 bound p38γ was predicted with the added restraint of a p38α DFG-out template (1WBT), which has 63% sequence identity to aligned regions in p38γ. The activated model was generated with the activated structure of p38γ (1CM8) as a template.

## Additional Information

**How to cite this article**: Aoto, P. C. *et al*. NMR Characterization of Information Flow and Allosteric Communities in the MAP Kinase p38γ. *Sci. Rep*. **6**, 28655; doi: 10.1038/srep28655 (2016).

## Supplementary Material

Supplementary Information
