# Supplementary material for: NMR Characterization of Information Flow and Allosteric Communities in the MAP Kinase p38γ
Source: Sci Rep. 2016 Jun 29;6:28655. doi: 10.1038/srep28655 (PMC4926091; doi:10.1038/srep28655)
Supplement: Supplementary Information [file srep28655-s1.pdf]

NMR Characterization of Information Flow and Allosteric Communities in the MAP Kinase p38γ

Phillip C. Aoto, Bryan T. Martin, and Peter E. Wright*

Supplementary Information

Protein expression and purification

Specifically methyl protonated samples (Ile δ1, Val γ1 or γ2, Leu δ1 or δ2) for 3D side-chain assignment were expressed in cells grown in M9 minimal media in D_2O and ^2H glucose supplemented with Val/Leu precursor α-ketoisovalerate (2-keto-3-methyl-d_3-3-d_1-1,2,3,4- ^{13}C butyrate) and Ile precursor α-keto-butyrate (2-keto-3,3,-d_2-1,2,3,4 ^{13}C butyrate) [1]. Protein was purified and exchanged into NMR buffer as described in the main text, except that samples used for backbone assignments were collected at pH 6.8 in 10% D_2O.

NMR spectroscopy

Ile, Leu, and Val methyl side-chain resonances were assigned using methyl to backbone correlation experiments with specifically labeled methyl residues [2]. 3D side-chain correlation spectra were acquired at 293K using 30-50% non-uniform sampling (NUS) on a Bruker Avance III 800MHz spectrometer equipped with cryoprobe. Spectra for backbone assignments were acquired at 293K on Bruker Avance 750MHz and 900MHz spectrometers equipped with triple resonance gradient probes. Amide ^1H-^{15}N, ^{13}C CO, Cα, and Cβ resonances were assigned using 3D TROSY HNCA, HNCACB, HNCOCA, and HNCO spectra acquired with 30-50% NUS. Sinusoidal weighted Poisson gap sampling schemes were generated as in [3] and optimal schemes were selected [4] for use in the ^{13}C indirect dimension. The ^{15}N constant time dimension was acquired with uniform sampling. NUS was implemented on 750MHz and 900MHz spectrometers by modification of the pulse program to include explicit looping based on an increment list corresponding to the NUS scheme. Mutagenesis described in the main text was used to supplement

assignments for residues that lacked backbone assignments or which had degenerate Cα and Cβ chemical shifts, and also to assign Met ε-methyls. A ^{13}C NOESY experiment was also acquired to aid in side chain assignment. Stereospecific methyl assignments were made with methyl CHD_2 labeled samples using a constant-time ^{13}C HSQC experiment as described in [5,6].

Chemical shift assignments

Assignment of methyls by triple resonance requires prior assignment of Cα and Cβ chemical shifts. The ^{1}H-^{15}N TROSY spectrum of His-tagged apo p38γ contains 364 out of an expected 368 backbone resonances (Fig. S1a). Although a high percentage of expected cross peaks are observed, many peaks appear to be undergoing exchange broadening, and their cross peak intensities are near the noise and their linewidths are broad. As a consequence only 285 amide resonances or 77% of the backbone, excluding the His tag and prolines, could be assigned (Fig. S2). The backbone resonances of most regulatory regions of p38γ were assigned, including the activation loop, DFG loop, αC-helix, αF-helix, MAPK insert, and docking site. Secondary structure predictions from TALOS+, using the assigned backbone chemical shifts, accurately predicts the known secondary structure of p38γ (Fig. S2), giving confidence in the correctness of the assignments. In the ^{1}H-^{13}C HMQC spectrum of MILV specifically labeled His-tagged p38γ, 162 out of 164 expected resonances (Met(ε), Ile(δ), Leu(δ1/δ2), Val(γ1/γ2) were observed (Fig. S1b). Assignments for 121 or 73% of the methyl resonances were made via a tripartite method that combined triple resonance experiments correlating the assigned backbone chemical shift to the methyl resonances, ^{13}C NOESY spectra to establish through-space connectivities, and single site mutagenesis (Fig. S2). All 13 Met ε-methyls, as well as all 13 Ile δ-methyls were assigned. One or both δ and γ methyls were assigned for 32 out of 41 leucines and 24 out of 28 valines, respectively. Stereospecific assignment for 90% of the Leu and Val methyl assignments was accomplished. Assignments were transferred to the spectra of inactive ATP bound, inactive BIRB796 bound, and activated apo states.

Changes in highly connected residues

Most residues with a high number of network connections (in the top third) in inactive apo p38γ surround the active site and docking site (Fig. S6). To maintain signaling homeostasis, inactive p38γ must remain catalytically inert yet exist in a state that is primed for activation by phosphorylation. This is clearly reflected in the network; the active site is highly connected and is sensitive to interactions throughout the protein. The docking site is also highly connected, consistent with its role in recruiting upstream kinases and facilitation of the conformational rearrangement of the activation loop necessary for phosphorylation [7]. Other regions of high connectivity correspond to elements involved in non-canonical activation, such as the MAPK insert and the αC-helix [8-10].

The overall network structure and flow suggests that the C-lobe in the inactive ATP bound and activated states has regulatory significance, as indicated by an increase in the number of highly connected residues in the C-lobe and a decrease in the N-lobe, relative to the inactive apo state (Fig. S6). The binding of ATP to inactive p38γ likely stabilizes a closed conformation, similar to that observed in activated kinases [11,12]. However, activation leads to proper packing of active site residues and further consolidates highly connected residues around the R-spine (Fig. S6). Residues in the C-lobe with high connectivity in the compact forms of p38γ (activated and ATP-bound) surround the αF-helix, GHI subdomain, αEF-helix, and P+1 loop (Fig. S7). These regulatory motifs are members of the architectural core of eukaryotic kinases and anchor the allosteric spines, interface with the catalytic loop, and also bind substrate.

Contacts in the C-lobe

Highly connected residues identified from the network analysis of the compact forms of p38γ surround Y191 in the P+1 loop, which forms an electrostatic and hydrophobic junction between P+1, αF-helix, and hydrophobic core (Fig. S7a). The P+1 loop interacts with substrate residues immediately adjacent to the substrate

phospho-acceptor site. This region, including Y191, has been shown to play a role in coupling the catalytically relevant open to closed domain movement in PKA [13]. Residues with high connectivity also suggest the GHI subdomain has an important role in the network. The GHI subdomain participates in various regulatory and allosteric interactions in eukaryotic kinases and is connected through hydrophobic interactions to the α-EF and F-helices, which indirectly access the active site [14]. Examples of regulation through GHI subdomain interactions include myristate binding in c-Abl and allosteric inhibitors for Bcr-Abl, which access a pocket above the GHI subdomain, between the H, E, and F-helices [15]. This binding pocket is adjacent to the highly connected residues in the compact forms of p38γ (Fig. S7b). Although myristate and derived inhibitors are thought to regulate c-Abl by αI-helix disruption and subsequent stabilization of SH2/SH3 domain docking [15,16], it is possible that myristate binding also perturbs the network in this highly connected region. The myristate pocket in c-Abl does not exist in p38γ, suggesting different kinases may exploit this critical node in different ways.

Pathways of docking site mediated auto-activation

The pathways described in Fig. 7 may be involved in docking site mediated auto-activation. A pivot around the hinge between the N and C-lobes facilitates the opening and closing of the kinase. Along with this movement, change in the orientation of M112 in the hinge has been suggested to allow ATP binding [17]. The influence that docking site residues have on the hinge, as revealed by the mediation analysis, may thus have consequences on stabilization of the open (inactive) or closed (active) states as well as the propensity for ATP binding. Indeed, cooperativity between docking site interactions and nucleotide binding has been observed experimentally in activated p38α [11]. Here, we also observe that the docking site is indirectly connected to the αC-helix and L16 loop. The position of the αC-helix is thought to influence the alignment of catalytic residues in kinases [8] and it has been proposed that the αC-helix position is stabilized by the L16 loop in MAPKs [18-20]. Taken together, the docking site's influence on the hinge and its effect on active-site residues revealed by the network analysis corroborate a mechanism

proposed for docking site induced auto-activation of p38α by TGFβ-activated kinase 1 binding protein (TAB1), whereby TAB1 docking peptide binding increases the p38α affinity for ATP and facilitates closure of N and C-lobes and rearrangement of the αC-helix, leading to auto-phosphorylation [21]. The critical flow demonstrates that overlapping and redundant pathways connect the docking site to the activation loop and catalytic residues.

Reducing local-effects of mutation on correlations

Spearman ranked correlation coefficients were used in the analysis and identify monotonic relationships, whereas Pearson's correlation coefficient (unranked) will only identify linear relationships. Methyls near mutation sites undergo large chemical shift perturbations relative to distant methyls. Thus, these large chemical shifts near sites of mutation may bias pairwise linear correlations (e.g. correctly identifying strong correlations between residues that are in similar proximity to multiple mutation sites; but giving weak or failing to find linear correlations between residues that are not near the same mutation sites). Ranking, thus allows non-linear but monotonic relationships to be properly identified for use in the network analysis described within. As described in the main text, negative correlations were discarded for simplicity of analysis. We could not rationalize an interpretation of negative correlations of chemical shift differences in the context of our network analysis utilizing information flow. However, important information about the network may be included in negative correlations and their incorporation into the described network analysis may be an area of future study.

Minimum number of mutations required for network analysis

The network analysis only reveals relationships that are perturbed by the mutations. Thus, to thoroughly describe a protein's network structure mutations need to be dispersed throughout the protein. Obviously, the most comprehensive network structure will result from 1) the greatest number of mutants possible (and number of observed resonance perturbations) and 2) when mutants are chosen to sample the entire protein. The enrichment of 2-point correlation p-values can be

used to determine if an adequate number of mutants are being examined for the network analysis of a system.

To determine the minimum number of mutations required for accurate description of the networks of p38γ, datasets for individual mutations within the apo inactive state of p38γ were randomly removed from the analysis until the original network structure was no longer reproduced. With the 78 observed methyl perturbations at least 11 mutations were necessary to reproduce the principal features of the network structure described in the main text (which used 20 mutants). See Figure S9 for details of the network structure derived from 11 mutations (L77V, M109L, M112L, M120L, M137L, L159V, L174V, L198V, M216L, I238L, M291L) -see main text Figure 3 for comparison. Within the set of 11 mutations that led to successful identification of the network, individual mutant datasets were replaced one at a time from the total set of 20 mutant datasets. Each of the combinations results in a network structure that is similar to the original network (derived with 20 mutants) and gives similar enhancements in one-tailed p-values for two point correlations. The enhancement of one-tailed p-values for two point correlations predicts the performance of the network analysis.

Supplementary Figures

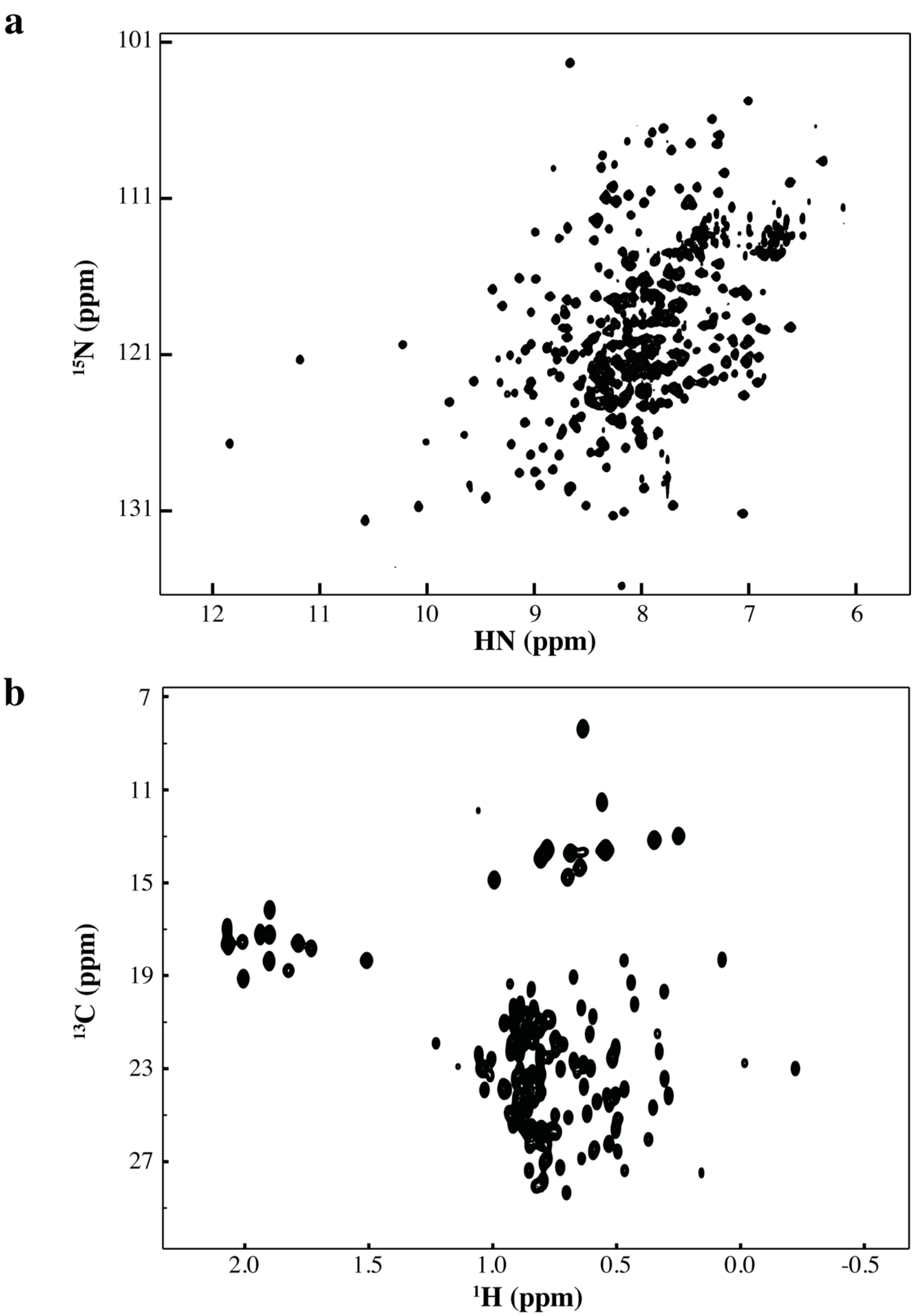

Figure S1. NMR spectra of His-tagged apo p38γ. a) ^1H-^{15}N TROSY of uniformly 15N-labeled and b) ^1H-^{13}C HMQC of MILV specifically labeled apo p38γ.

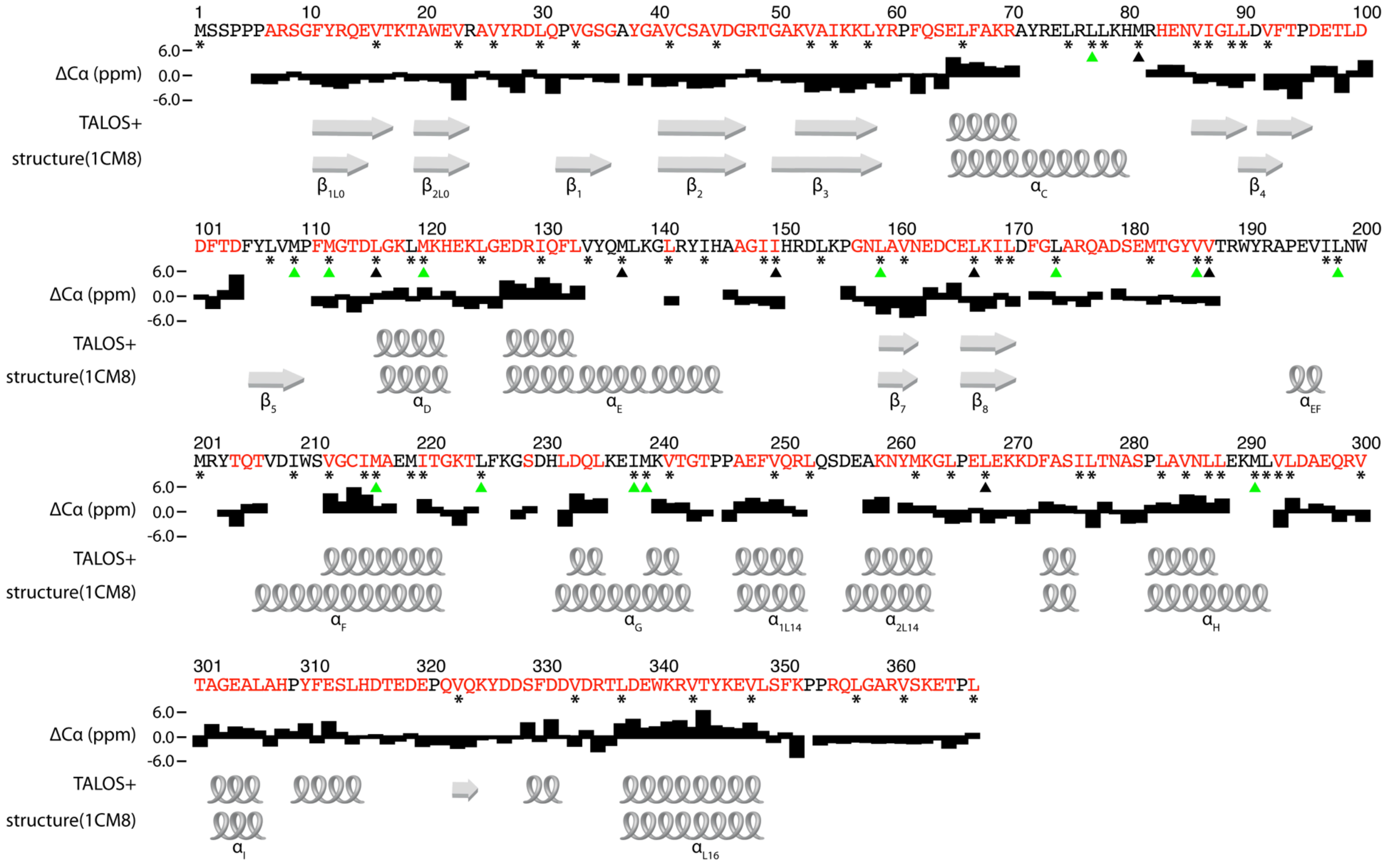
1 10 20 30 40 50 60 70 80 90 100
MSSPPPARSGFYRQEVTKTAWEVRAVYRDLQPVGSGAYGAVCSAVDGRTGAKVAIKKLYRPFQSELFAKRAYRELRLLKHMRHENVIGLLDVFTPDETLD
101 110 120 130 140 150 160 170 180 190 200
DFTDFYLVMPFMGTDLGKLMKHEKLGEDRIQFLVYQMLKGLRYIHAAGIIHRDLKPGNLAVNEDCELKILDFGLARQADSEMTGYVVTRWYRAPEVILNW
201 210 220 230 240 250 260 270 280 290 300
MRYTQTVDIWSVGCIMAEMITGKTLFKGSDHLDQLKEIMKVTGTPPAEFVQRLQSDEAKNYMKGLPELEKKDFASILTNASPLAVNLLEKMLVLDAEQRV
301 310 320 330 340 350 360
TAGEALAHPYFESLHDTEDEPQVQKYDDSFDDVDRTLDEWKRVTYKEVLSFKPPRQLGARVSKETPL
ΔCα (ppm)
6.0
0.0
-6.0
TALOS+
structure(1CM8)
β1L0 β2L0 β1 β2 β3 αC β4
β5 αD αE β7 β8 αEF
αF αG α1L14 α2L14 αH
αI αL16

Figure S2. NMR assignments and secondary structure of p38γ. Residues for which backbone assignments could be made are shown in red, and side chain methyl assignments are indicated by asterisks. Deviations of $^{13}C\alpha$ chemical shifts from random coil values are shown as black bars. The 20 methyl containing side chains subjected to conservative mutagenesis are indicated by triangles (green and black). The 13 mutations used in the inactive ATP bound, BIRB796 bound, and activated states are shown as green triangles.

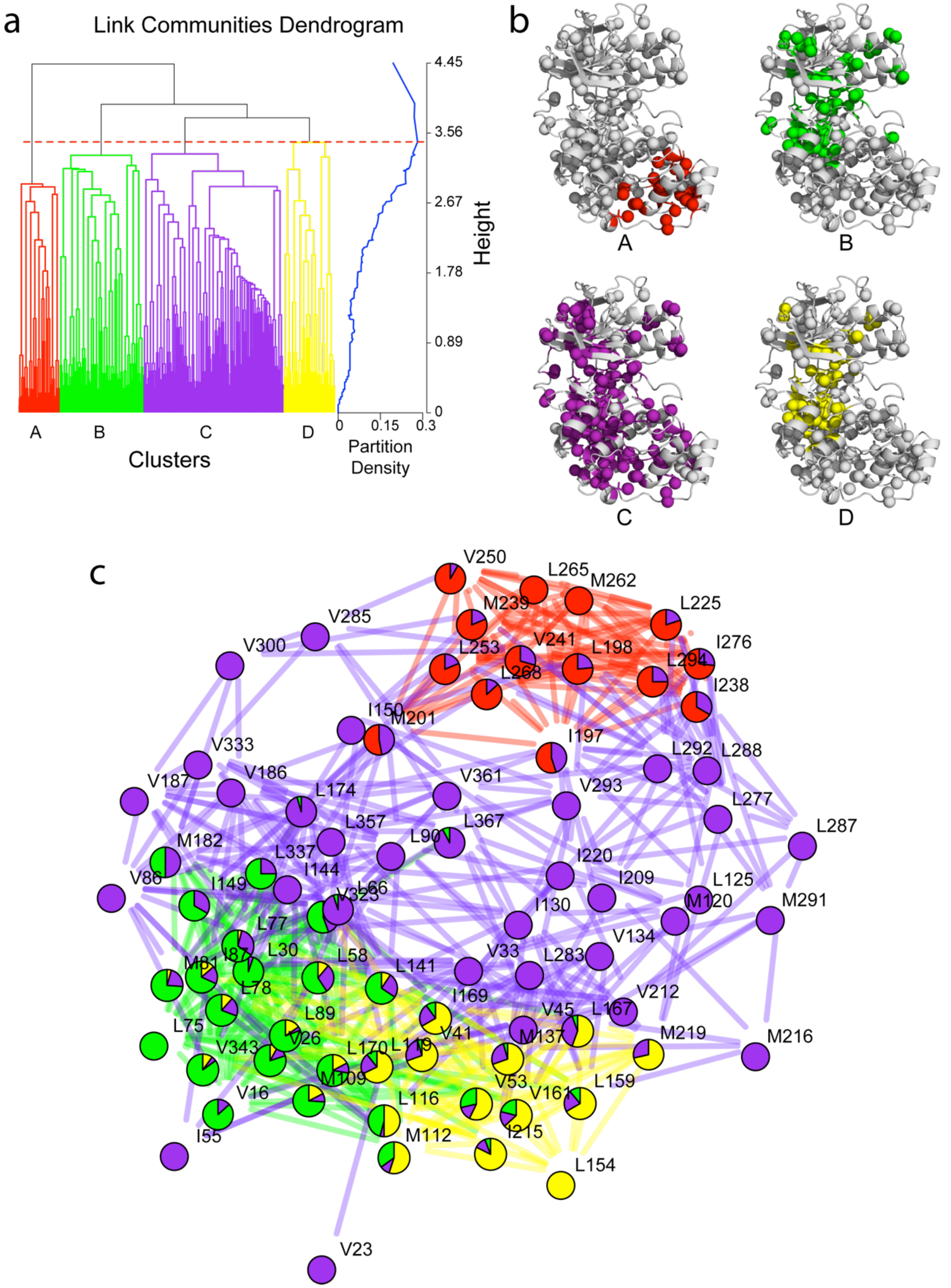

Figure S3. Link communities of inactive apo p38γ. Overlapping communities and hierarchy were identified by the link community method [22] [23] from two-point correlations of chemical shift perturbations resulting from conservative

mutagenesis. a) Communities are grouped links (paired residues). The partition density function described in [22] was used to determine a cut-off (red dashed line) to obtain the 4 communities. b) Link communities in a) plotted by color on the structure of p38γ. The communities are similar to those in Fig. 3 and correspond to the MAPK-insert (red), N-lobe (green), entire protein (purple), and the active-site/docking-site (yellow). Some communities identified in Fig. 3 are not identified here, such as the R and C-spines, and the C-lobe, instead they are included in a single community encompassing the entire protein (purple), which was not identified in the results presented in Fig. 3. c) Network graph of link communities, demonstrating residue (node) connections and overlap. Nodes (circles) represent residues in p38γ. The community membership of each residue is indicated by pie-chart color corresponding to communities in a).

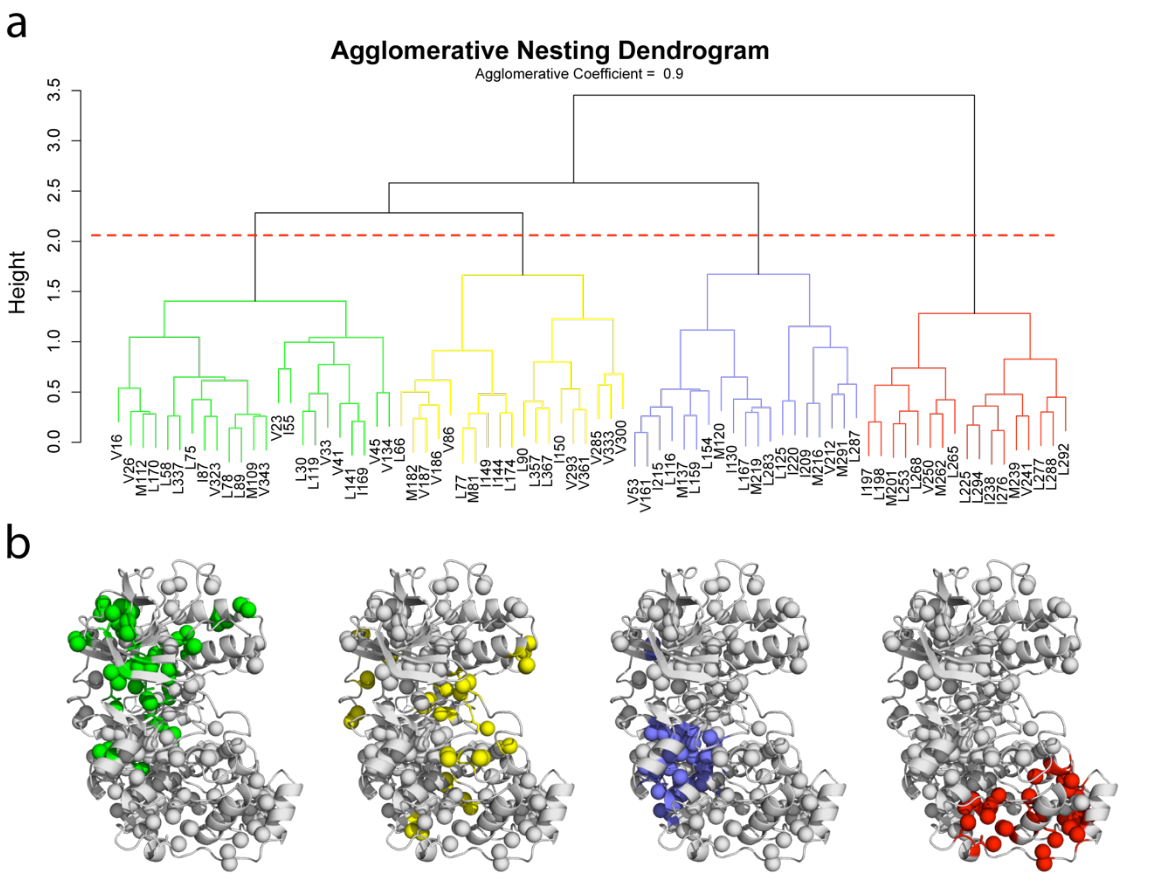

Figure S4. Agglomerative hierarchical clustering of inactive apo p38γ. a) Non-overlapping communities and hierarchy were identified by agglomerative clustering

of two-point correlations of chemical shift perturbations caused by conservative mutagenesis using Ward's method within the agnes function in R [24]. A threshold (red dashed line) was chosen to give 4 communities. b) Communities in a) plotted by color on the structure of p38γ. The communities correspond to regulatory elements, similar to those in Fig. 3: N-lobe/C-spine (green), R-spine (yellow), docking-site (blue), MAPK-insert (red). Some of the differences in results from Fig. 3, such as the docking-site having a distinct community and the absence of a C-lobe community, is likely due to the inability of the agglomerative clustering method to identify overlapping communities.

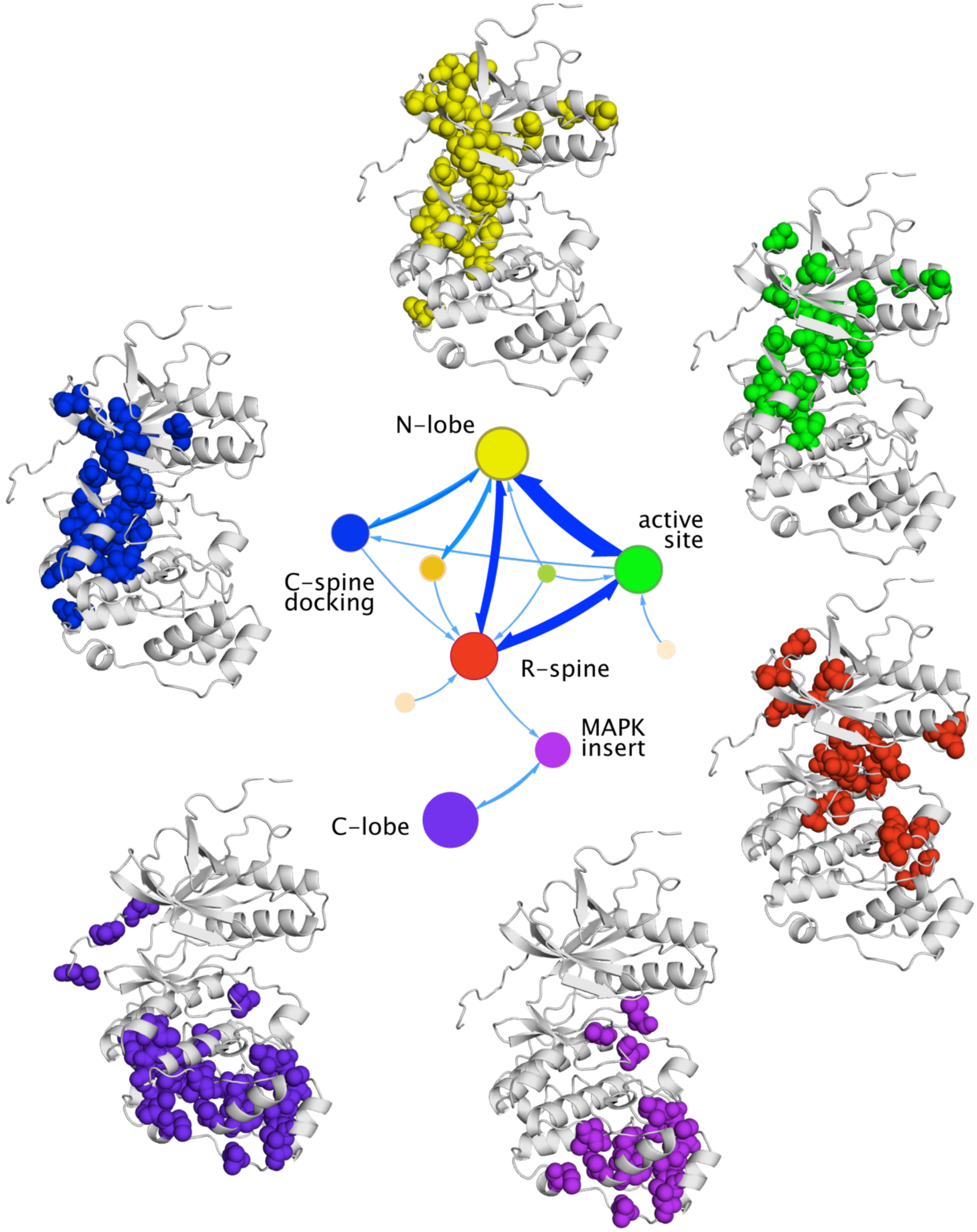

Figure S5. Network structure and flow for inactive apo p38γ derived from a limited set of mutants. Identified communities from chemical shift perturbation networks are colored based on tertiary structure and regulatory element: N-lobe, yellow; C-lobe, purple; active-site, green; C-spine, blue; R-spine, red; MAPK-insert, lavender. The size of the modules represents the amount of flux and connections between residues within the module. Thickness of arrows between communities represents

the amount of flux between communities. Using a common set of 13 mutants of inactive apo p38γ (used also for ATP bound, BIRB796 bound, and activated states) in the network analysis yields clustered communities similar to the analysis with 20 mutants described in the main text (see Fig. 3). One noticeable difference is that the module most closely corresponding to the 'R-spine' residues is linked to the C-lobe and MAPK insert in this analysis, whereas the results from 20 mutants has the active site community linked to the C-lobe and MAPK insert. The R-spine and active site communities are overlapped so that this does not reflect a major change in the network structure. The remainder of the network connections are very similar for the analysis performed using data from 13 or 20 mutants. The relative flow between communities is also similar.

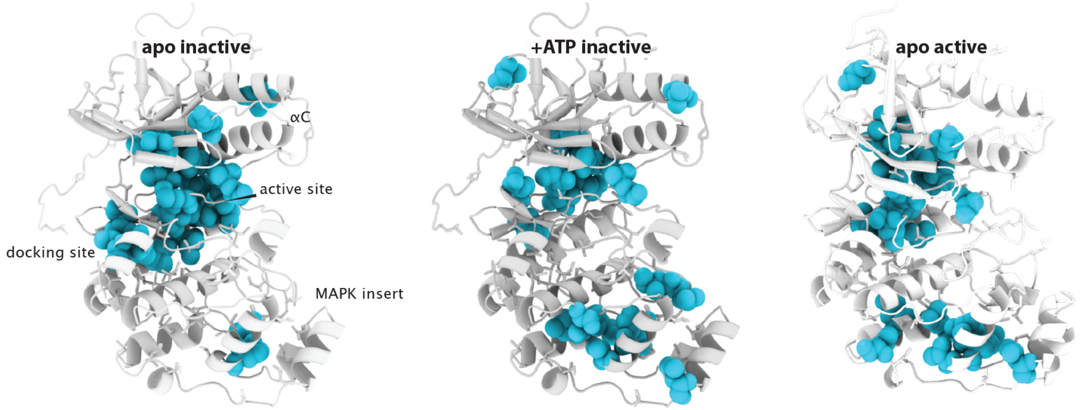

Figure S6. Critical network nodes reveal regulatory sites and pathways. Residues with a large number of connections to the network (cyan spheres) highlight regions that are important in the function and regulation of p38γ. Changes in state of p38γ cause reorganization of the network, illustrated by redistribution of highly connected residues. An increase in connected residues in a regulatory region indicates the region has greater influence on the network.

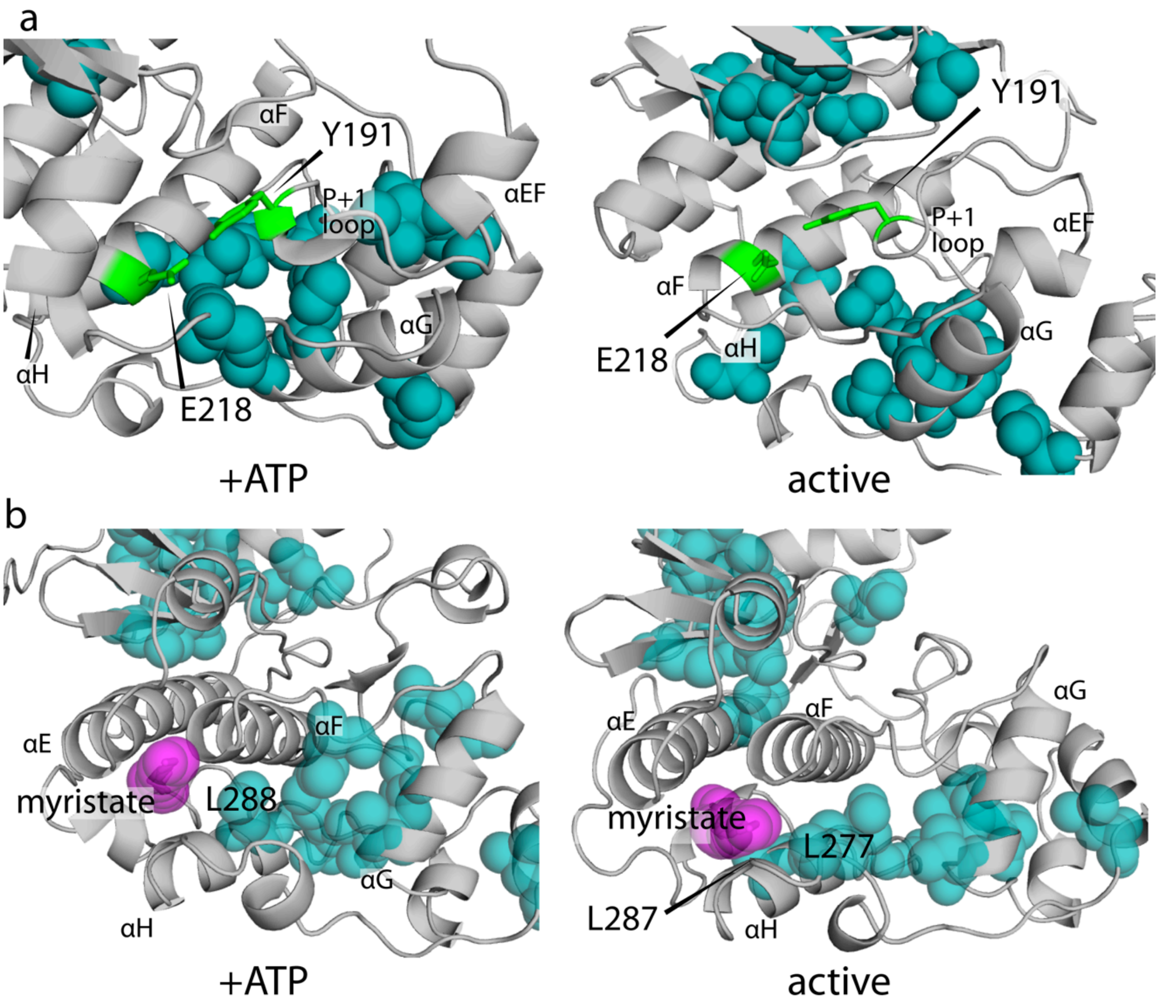

Figure S7. Residues with high network connectivity in the C-lobe of compact forms of p38γ surround the architectural core of eukaryotic kinases. a) Y191 in the P+1 loop and E218 in the F-helix constitute an electrostatic and hydrophobic node coupling the N and C-lobes and active site [13]. Residues with high connectivity (cyan spheres) surround Y191 and the P+1 loop in the inactive ATP bound and activated forms of p38γ. b) An abundance of highly connected residues are found in the GHI subdomain in inactive ATP bound and activated forms of p38γ. An example of regulation via interaction with the GHI subdomain is myristate (purple spheres) binding to c-Abl. The c-Abl myristate binding site's proximity to the clusters of highly connected residues in p38γ (cyan spheres) is illustrated.

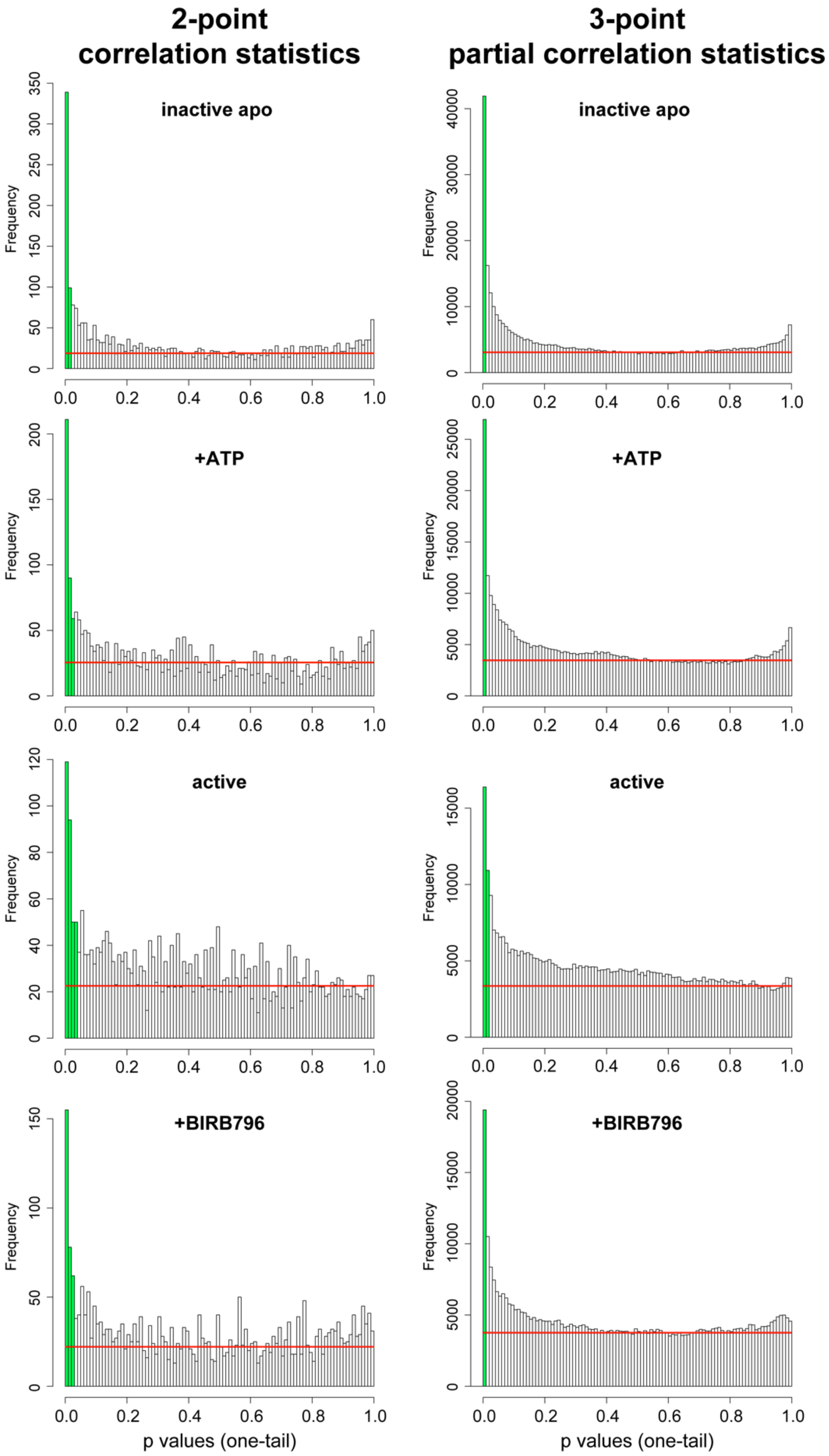
2-point
correlation statistics
3-point
partial correlation statistics
inactive apo
+ATP
active
+BIRB796
Frequency
0
50
100
150
200
250
300
350
0
10000
20000
30000
40000
5000
15000
25000
20
40
60
80
120
0.0
0.2
0.4
0.6
0.8
1.0
p values (one-tail)

Figure S8. Two point correlation and multiple regression statistics. One tailed p-value histograms from t-tests of two-point ranked correlations (left) and three-point multiple regression partial correlations (right) are shown. Critical p-values were chosen (green) for enrichment of the alternative hypothesis over the uniformly distributed null p-values (approximated by red line). Uncorrected p-values <= 0.01 (0.005) were considered significant for 2 (3) point correlations of inactive apo p38γ. Uncorrected p-values <= 0.03 (0.015) were considered significant for 2(3) point correlations of inactive p38γ complexed with ATP or BIRB796. In the active apo state uncorrected p-values <= 0.04 (0.02) were considered significant for 2(3) point correlations. A soft-threshold was used on two-point correlation coefficients for probabilities of teleportation in the MapEquation analysis, as described in the main text. Three point multiple regression partial correlation coefficients were used as described in the main text in the calculation of three-residue flow for MapEquation.

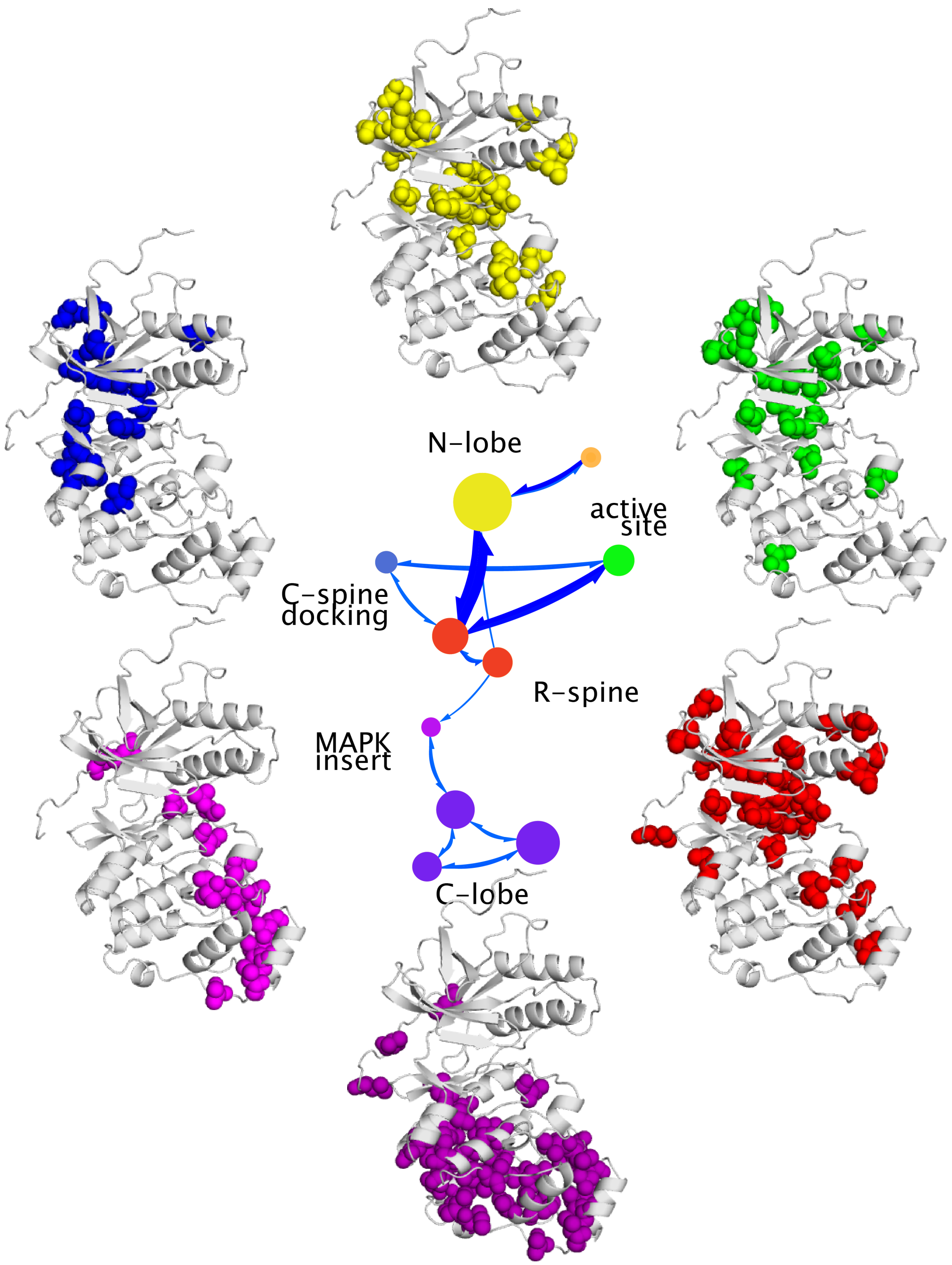

Figure S9. Representative network structure and flow for inactive apo p38γ derived from a minimal set of mutants. Identified communities from chemical shift perturbation networks are colored based on tertiary structure and regulatory element: N-lobe, yellow; C-lobe, purple; active-site, green; C-spine, blue; R-spine, red; MAPK-insert, lavender. The size of the modules represents the amount of flux

and connections between residues within the module. Thickness of arrows between communities represents the amount of flux between communities. Using a minimal set of 11 mutants of inactive apo p38γ in the network analysis yields clustered communities similar to the analysis with 20 mutants (and 13 mutants) described in the text (see Fig. 3 and Fig. S5). One noticeable difference is that the module most closely corresponding to the 'R-spine' residues is linked to the C-lobe and MAPK insert in this analysis, whereas the results from 20 mutants has the active site community linked to the C-lobe and MAPK insert. The R-spine and active site communities are overlapped so that this does not reflect a major change in the network structure. The overall network structure is the same but some communities are split (i.e. using a greater number of mutants the C-lobe is a single community, here it is represented by 3 highly overlapped communities). The relative flow between communities is also similar.

Table S1. ^{1}H-^{13}C average chemical shift perturbations from wildtype inactive apo p38γ (Hz)

Residue	L77V	M81L	M109L	M112L	L116V	M120L	M137L	I150L	L159V	L167V	L174V	V186A	V187A	L198V	M216L	L225V	I238L	M239L	L268V	M291L
**16**	6.1	3.3	7.1	12.5	8.8	7.2	3.5	6.0	4.1	4.9	7.8	2.1	7.6	5.0	1.9	4.0	1.8	4.3	0.8	3.0
**23**	4.0	2.6	6.7	5.2	5.1	3.5	2.2	4.1	4.9	4.2	3.3	3.8	3.7	4.2	1.4	8.1	1.7	1.3	4.1	4.6
**26**	7.5	10.8	11.9	5.3	3.4	5.3	3.2	2.8	1.9	5.1	6.1	1.3	3.2	1.8	1.6	1.8	1.3	1.7	3.0	1.8
**30**	5.4	8.2	10.6	4.1	7.0	0.6	3.4	7.2	4.7	5.3	6.6	1.5	6.9	10.7	3.5	3.4	4.4	0.0	1.1	3.2
**33**	3.0	7.5	5.1	14.2	4.8	4.3	4.2	5.7	0.8	7.2	4.7	0.9	3.0	6.2	5.8	3.6	1.1	0.0	0.8	4.8
**41**	5.5	7.4	9.1	18.7	8.6	0.4	9.7	7.3	8.4	4.2	9.2	4.1	7.4	5.2	10.0	4.2	3.4	0.0	3.9	10.1
**45**	8.7	2.6	5.2	17.9	6.9	1.1	6.9	5.1	10.3	11.2	10.3	5.7	11.4	6.8	6.2	7.0	5.7	3.0	3.7	4.1
**53**	1.7	7.8	8.7	31.1	10.4	8.5	9.6	5.7	7.6	8.9	3.3	1.2	4.6	5.0	7.6	2.8	1.8	0.0	1.2	8.2
**55**	1.4	15.4	17.0	4.8	3.4	0.0	1.5	4.8	3.8	4.7	4.8	3.3	5.3	1.2	5.3	3.5	3.1	0.0	5.6	8.6
**58**	8.7	12.8	8.7	3.0	2.5	0.9	3.3	5.1	3.6	2.1	14.4	1.1	5.9	2.6	3.5	1.6	1.5	0.0	1.3	3.2
**66**	14.0	19.5	7.4	2.4	5.6	5.8	3.9	19.8	7.7	11.7	12.5	10.1	7.5	3.5	4.4	8.9	0.0	3.8	1.5	2.9
**75**	8.3	4.3	10.1	4.0	2.8	1.3	4.2	5.2	2.5	0.6	1.1	2.0	4.3	4.2	1.4	1.5	0.9	0.0	0.6	2.4
**77**	141.4	144.5	7.9	18.8	11.7	10.7	42.2	48.1	19.7	4.5	93.0	15.0	15.8	9.9	7.7	5.5	4.5	8.1	1.8	9.0
**78**	100.9	54.8	25.0	13.9	13.8	8.1	13.8	11.6	12.8	4.8	39.4	7.6	9.8	5.1	4.7	4.2	5.0	7.9	0.0	6.9
**81**	22.3	383.9	8.4	17.5	15.6	10.9	36.4	53.3	24.7	4.0	134.8	13.9	5.5	6.6	5.3	4.1	4.5	2.3	5.3	2.4
**86**	14.8	13.8	0.0	12.6	3.8	4.9	2.4	3.1	1.9	3.3	26.7	3.6	11.9	1.8	0.8	4.5	2.7	0.0	1.7	3.4
**87**	25.3	33.9	31.5	39.0	4.8	1.5	3.0	8.4	13.7	1.4	20.4	3.8	5.3	7.7	4.6	6.8	7.6	0.0	2.6	7.5
**89**	42.4	17.6	117.0	16.1	15.7	5.1	11.8	10.7	12.1	9.0	15.8	6.0	6.0	5.6	5.9	4.1	6.7	5.7	3.4	10.7
**90**	6.5	17.9	0.0	1.1	1.9	2.7	8.4	5.0	5.3	8.9	18.5	5.3	6.6	9.4	4.4	4.0	3.1	4.2	1.9	4.1
**109**	17.2	30.9	117.0	13.2	7.4	3.8	9.7	5.3	13.8	1.2	12.1	1.3	0.9	0.8	3.8	1.5	1.4	0.0	4.9	4.7
**112**	12.7	9.0	23.2	60.6	10.6	11.3	10.6	7.9	18.6	55.5	8.8	3.8	4.8	5.5	6.8	5.7	0.6	8.2	1.8	1.5
**116**	12.3	13.3	15.6	13.4	177.4	24.4	27.0	14.2	49.9	12.9	14.0	3.3	10.3	12.6	4.9	9.9	5.3	7.5	4.1	14.9
**119**	3.2	4.8	7.9	12.1	3.4	0.0	4.9	5.6	7.7	16.0	6.4	0.2	5.3	6.1	2.3	2.9	2.0	0.0	0.5	3.1
**120**	4.7	5.3	0.8	3.0	71.3	29.5	3.8	5.5	1.2	5.3	5.3	1.9	5.3	11.3	9.1	10.5	3.8	0.0	0.8	2.0
**125**	4.7	10.2	9.4	11.6	7.6	29.5	19.8	20.7	4.8	5.5	3.2	4.9	8.3	12.0	10.3	8.3	4.9	6.6	10.8	25.0
**130**	7.0	9.8	0.0	5.8	12.0	6.0	12.9	18.6	17.4	6.8	8.0	3.6	7.2	5.4	16.0	10.1	4.2	7.1	3.4	5.9
**134**	2.9	1.4	0.0	6.0	1.6	1.5	12.8	3.3	14.5	13.6	1.1	2.9	7.8	4.1	3.1	1.5	2.4	0.0	1.5	18.7
**137**	32.0	40.5	11.4	11.5	41.5	10.3	98.0	6.7	119.0	42.1	19.2	1.9	6.0	23.9	21.3	3.0	5.1	8.8	7.5	34.0
**141**	21.9	53.1	10.6	23.1	20.5	5.3	28.8	15.0	29.4	18.9	30.3	12.0	6.1	11.5	13.0	7.1	6.1	7.2	2.1	13.7
**144**	58.0	56.9	6.0	14.0	13.0	6.5	40.8	59.3	39.2	15.4	81.4	7.9	8.2	11.4	11.2	6.8	11.9	14.7	8.9	9.5
**149**	141.4	383.9	7.6	25.2	18.4	5.5	53.4	37.1	3.6	5.4	164.6	13.3	9.1	22.6	2.4	4.0	5.5	11.3	1.5	6.1
**150**	14.3	17.0	7.8	6.2	2.4	7.9	4.2	85.5	4.5	4.8	19.9	2.3	7.7	22.9	18.6	6.0	8.7	13.9	5.5	20.3
**154**	3.8	19.2	16.2	12.6	64.2	19.0	50.4	23.2	14.8	42.1	1.9	6.7	9.2	13.0	9.5	4.7	9.3	18.4	6.2	6.0
**159**	3.6	18.9	9.3	5.6	78.8	11.7	45.3	13.8	119.0	115.5	4.8	4.2	11.3	5.6	12.3	1.9	1.8	8.6	2.3	29.3
**161**	2.4	6.7	11.1	38.1	100.2	9.6	20.4	6.0	60.8	84.3	5.5	2.2	7.7	3.7	10.2	3.5	3.8	0.5	3.1	6.6
**167**	11.5	30.0	8.7	8.3	87.6	16.1	53.7	12.1	73.6	115.5	13.0	10.2	7.7	11.6	17.0	12.5	7.6	9.1	9.8	9.9
**169**	7.7	24.1	10.4	18.8	17.7	4.9	90.7	46.3	54.3	11.5	18.3	3.4	7.6	9.3	19.4	5.8	8.6	7.1	2.7	14.2
**170**	11.3	34.7	33.8	35.8	16.1	9.2	20.7	31.5	15.5	22.2	14.0	8.6	4.4	3.0	4.7	1.6	4.0	1.9	4.1	5.5
**174**	112.8	46.9	3.9	18.7	4.9	6.5	26.1	85.5	23.3	6.1	164.6	9.4	25.9	11.9	3.9	5.3	11.1	8.3	10.1	11.7
**182**	27.2	22.1	6.6	3.3	5.5	4.4	4.7	8.4	11.6	6.2	30.2	27.2	13.0	4.4	1.5	2.7	8.8	5.1	1.7	1.7
**186**	14.6	11.9	1.9	6.7	1.2	3.8	2.8	17.5	6.5	4.2	8.0	38.3	45.5	8.7	3.6	4.8	6.1	3.8	3.3	4.4
**187**	9.5	20.3	2.8	5.0	3.2	0.8	4.4	20.4	5.4	4.8	17.1	22.5	70.3	1.9	3.4	4.3	8.4	6.8	1.0	3.0
**197**	6.4	12.1	2.9	10.0	5.6	6.6	28.5	44.2	14.1	8.4	8.7	6.3	22.7	51.8	12.9	15.0	31.6	9.8	16.3	19.0
**198**	5.5	12.8	3.6	6.8	5.6	11.4	13.0	30.9	14.2	8.2	15.5	6.8	15.8	51.8	16.6	8.0	29.0	18.1	21.8	15.3
**201**	14.2	30.4	11.7	12.2	4.1	10.8	12.0	16.8	12.7	8.6	21.4	6.0	15.9	17.5	7.8	8.2	15.6	28.8	14.4	12.8
**209**	22.8	7.6	2.1	6.8	12.3	7.0	82.4	6.8	9.6	6.8	15.3	4.0	10.4	49.9	40.3	5.3	1.5	20.0	10.8	22.7
**212**	7.5	14.5	6.1	4.6	4.0	5.2	20.0	3.7	19.1	5.1	6.9	4.3	2.8	7.0	15.9	3.4	8.3	2.4	4.5	13.8
**215**	5.3	19.9	17.9	15.9	22.8	14.1	20.8	10.7	109.1	14.1	10.1	3.5	13.6	10.0	11.7	18.0	11.4	3.6	3.9	11.3
**216**	6.0	3.0	0.8	3.8	11.3	11.7	4.0	0.8	8.2	17.7	11.7	2.1	4.7	5.2	115.3	3.7	1.9	12.0	3.8	31.9
**219**	4.6	3.5	7.6	2.4	95.7	14.6	36.4	13.7	21.2	15.2	3.5	1.8	6.6	13.4	33.1	16.6	1.3	8.3	1.3	4.5
**220**	6.6	15.6	2.9	7.6	6.0	9.0	9.8	12.0	4.4	2.3	7.2	2.0	3.4	7.3	115.3	9.3	6.2	1.9	5.5	16.2
**225**	2.5	5.3	4.9	5.1	12.7	25.2	13.2	28.6	7.3	4.7	6.5	3.0	5.2	13.3	9.1	531.4	91.7	48.7	15.6	33.4
**238**	3.3	6.6	0.0	0.0	1.5	3.0	9.4	4.0	9.0	2.4	4.6	2.6	5.1	7.9	12.7	39.9	140.7	30.1	15.0	11.7
**239**	1.3	13.7	6.4	2.7	1.5	8.2	2.6	33.5	5.3	3.7	6.0	3.2	12.2	17.7	6.7	7.3	27.5	86.2	37.7	25.2
**241**	5.1	12.5	8.6	2.1	5.6	5.3	2.1	9.2	4.4	3.2	6.6	3.5	2.9	12.6	6.3	20.9	33.4	28.4	11.1	18.5
**250**	16.0	5.5	7.4	9.5	6.9	3.8	5.5	16.7	8.1	6.8	15.1	7.6	5.0	18.1	6.6	10.0	22.1	16.0	28.5	18.6
**253**	13.4	13.5	10.6	4.2	3.9	9.5	14.4	18.2	11.2	5.5	14.2	5.4	12.0	19.4	9.1	8.6	28.9	86.2	21.1	11.8
**262**	2.1	5.3	1.4	3.8	2.0	0.0	2.3	9.5	0.8	0.8	3.4	7.3	1.5	12.5	1.9	1.6	19.2	82.4	11.0	6.1
**265**	3.0	8.2	0.0	8.2	5.7	0.0	7.0	4.8	6.9	7.0	8.2	4.8	8.1	11.1	4.0	10.6	32.5	64.8	58.7	9.4
**268**	7.7	11.7	5.7	10.3	2.9	4.0	10.0	12.8	12.2	6.4	7.6	3.8	5.9	17.7	3.9	9.7	11.4	47.1	58.7	9.0
**276**	3.3	7.0	0.0	2.0	1.7	7.1	6.6	3.5	6.0	2.0	2.3	3.1	3.2	11.8	13.5	42.7	9.2	23.3	19.5	10.6
**277**	5.8	5.4	6.2	2.0	11.9	11.9	15.4	7.3	12.9	5.1	5.8	1.9	2.5	7.6	50.8	61.4	23.5	6.7	11.3	25.9
**283**	6.7	9.3	9.7	4.5	6.6	6.6	26.5	10.1	15.1	28.5	8.4	0.8	5.7	9.7	19.9	9.4	3.8	3.9	4.8	8.0
**285**	4.3	3.5	5.6	2.7	5.0	0.0	1.9	9.9	3.1	6.1	9.9	3.2	4.6	4.5	4.6	9.2	8.8	3.5	6.0	3.7
**287**	7.6	6.9	4.3	7.9	3.8	14.3	13.2	5.9	9.6	7.2	8.8	3.8	9.0	2.7	48.5	12.3	9.9	4.1	7.3	60.8
**288**	8.4	5.8	4.7	4.0	11.0	6.8	14.0	11.3	5.0	6.5	6.9	3.9	7.9	14.6	52.1	133.1	7.7	6.8	15.3	54.7
**291**	5.3	2.3	0.4	1.4	8.8	3.3	17.3	0.9	14.1	6.2	5.4	6.2	1.7	5.3	64.3	6.0	11.1	3.3	3.6	81.7
**292**	14.3	10.4	7.4	2.0	5.8	9.9	32.5	23.9	17.2	10.5	12.4	5.6	12.4	11.3	25.4	16.7	19.4	26.7	12.7	23.6
**293**	6.7	8.0	0.2	2.7	0.8	3.4	12.7	8.6	6.0	7.1	8.1	2.1	6.1	13.1	12.5	13.0	6.0	6.4	4.7	20.6
**294**	4.1	7.8	4.5	7.9	5.1	10.3	13.2	14.1	5.9	2.8	5.3	5.9	4.7	22.5	9.7	9.8	98.0	54.7	42.3	16.1
**300**	6.8	7.2	7.1	1.8	3.3	4.2	2.3	32.0	4.5	4.3	3.9	11.1	11.6	3.9	3.7	8.8	12.2	5.8	5.0	4.7
**323**	26.4	63.9	6.3	11.2	6.6	0.6	9.8	13.7	3.8	3.6	28.8	3.8	5.7	10.5	3.2	8.7	4.5	1.7	5.1	10.5
**333**	13.7	7.6	0.0	3.3	4.2	2.4	3.1	7.6	2.5	6.1	7.4	4.2	6.1	3.4	4.8	3.0	9.3	0.0	6.2	2.2
**337**	14.5	8.1	8.3	2.4	3.2	4.4	4.4	4.5	3.2	2.2	15.9	1.6	6.7	3.6	6.7	1.7	2.8	4.2	3.0	3.6
**343**	10.4	7.5	11.6	9.8	6.2	5.6	6.9	6.0	6.6	2.5	10.6	2.3	4.9	4.7	3.3	6.4	5.5	6.1	3.7	3.9
**357**	4.2	2.6	0.0	2.1	1.1	0.0	1.0	2.8	1.9	4.6	3.8	2.0	3.7	2.3	1.2	4.2	1.0	0.6	1.5	2.1
**361**	4.8	1.6	1.2	0.6	0.4	2.6	3.1	5.2	0.8	2.9	11.5	0.8	9.6	7.7	2.8	7.2	1.3	1.2	1.3	1.6
**367**	2.6	1.7	0.0	0.0	1.1	0.0	0.6	3.9	2.6	4.1	3.5	1.8	4.7	6.8	6.2	2.4	1.2	0.0	0.9	1.4

Table S2. ^{1}H-^{13}C average chemical shift perturbations from wildtype p38γ + ATP (Hz)

Residue	L77V	M109L	M112L	M120L	L159V	L174V	V186A	L198V	M216L	L225V	I238L	M239L	M291L
**16**	5.7	6.9	5.9	5.5	4.4	5.8	2.5	3.0	2.8	1.9	2.7	2.4	5.8
**23**	10.2	4.9	4.6	4.2	5.7	5.4	1.3	3.3	9.2	12.0	8.3	3.8	7.2
**26**	9.3	13.2	4.7	1.7	3.3	4.7	1.6	3.2	4.1	0.8	1.8	0.4	1.6
**30**	1.3	15.8	2.9	7.2	4.6	7.8	1.1	9.3	7.9	2.0	2.2	0.4	4.4
**33**	5.4	27.4	22.7	10.3	5.8	12.7	1.4	9.8	4.4	1.4	2.3	10.5	6.9
**41**	3.8	11.2	11.0	12.6	7.7	4.2	5.1	5.8	6.3	3.9	5.9	4.9	6.9
**45**	10.0	5.2	17.5	5.5	6.6	3.8	6.3	5.9	9.3	10.8	7.8	3.6	6.0
**53**	6.1	17.3	30.2	7.4	29.2	8.8	4.9	5.3	9.9	8.9	2.8	4.0	4.7
**55**	8.4	14.8	2.4	7.5	21.3	10.4	7.7	6.4	5.8	11.7	4.8	11.9	12.0
**58**	5.3	11.2	2.2	5.8	4.6	8.7	5.2	5.9	4.7	2.0	1.4	4.8	2.7
**66**	6.0	8.4	0.2	1.5	6.2	15.2	6.2	10.3	8.1	11.8	9.3	10.9	10.4
**75**	5.1	9.1	4.6	1.6	3.4	1.9	3.0	4.1	4.9	2.2	1.3	1.9	4.0
**77**	134.8	19.7	13.2	13.6	14.0	50.1	7.8	10.7	14.0	9.2	9.2	6.6	10.9
**78**	26.7	21.1	12.1	10.4	11.1	23.3	3.0	6.0	13.3	2.7	4.2	7.3	15.8
**81**	38.2	42.5	8.0	11.2	18.7	137.0	7.3	7.3	18.1	8.0	6.0	8.9	9.6
**86**	15.5	14.7	11.9	6.2	23.6	18.0	2.0	1.5	11.0	9.8	7.6	0.0	2.6
**87**	28.4	48.9	52.2	9.3	29.0	15.1	2.1	4.9	20.2	5.1	9.5	6.5	8.8
**89**	33.7	33.9	20.0	13.1	16.0	13.9	2.1	6.0	12.9	6.6	2.6	2.7	11.7
**90**	9.1	5.1	9.3	0.8	2.0	8.4	2.6	6.6	12.2	4.1	2.6	10.0	9.3
**109**	9.2	48.9	21.6	7.7	11.5	12.7	8.8	8.2	7.0	7.7	7.2	9.0	8.8
**112**	13.1	29.4	55.8	7.9	44.0	8.5	7.4	4.8	8.4	4.9	0.6	6.8	6.2
**116**	8.8	9.2	10.0	18.6	62.0	9.2	2.6	1.7	14.6	5.4	1.7	4.4	3.9
**119**	2.1	10.7	7.8	9.3	5.9	1.8	3.9	7.9	1.5	2.2	2.2	2.1	3.3
**120**	7.9	2.0	0.8	56.0	13.4	0.0	0.0	6.5	15.5	5.0	10.2	0.0	1.5
**125**	6.6	5.3	10.7	56.0	18.0	7.1	5.7	7.7	11.3	6.1	3.7	8.9	11.3
**130**	4.2	3.9	1.7	9.5	13.5	6.6	3.7	5.5	13.2	10.5	6.9	8.7	6.1
**134**	6.4	4.0	1.9	3.1	1.2	5.8	0.0	2.3	18.6	3.8	7.0	4.0	15.1
**137**	24.0	2.9	10.7	11.9	114.8	3.2	10.0	12.2	21.3	10.3	9.9	6.8	32.9
**141**	46.4	10.6	11.9	6.4	23.5	14.0	6.6	8.5	12.1	9.7	12.5	13.7	16.7
**144**	78.1	19.3	12.4	13.3	30.0	68.1	6.1	14.3	15.2	10.1	13.0	12.8	26.0
**149**	134.8	29.2	15.2	11.7	22.0	123.2	4.6	12.9	8.0	11.1	12.1	9.6	3.9
**150**	15.5	10.0	13.0	13.4	23.7	26.7	7.9	18.2	10.9	15.6	15.3	19.7	9.1
**154**	2.7	6.2	4.1	12.2	20.2	16.7	7.0	9.4	33.3	13.6	8.8	2.7	14.4
**159**	18.8	11.8	5.1	13.7	118.1	3.8	9.9	7.7	12.5	9.5	11.8	12.5	16.6
**161**	10.2	6.6	53.2	8.0	46.8	10.5	7.3	6.3	18.6	9.3	1.9	1.4	4.9
**167**	20.6	8.3	13.2	9.7	77.4	10.5	5.4	5.8	42.0	4.9	5.3	9.4	20.2
**169**	11.4	9.2	17.1	6.1	57.3	13.9	10.6	10.1	13.4	9.2	8.9	4.6	12.0
**170**	13.4	13.3	12.1	2.5	6.8	8.4	4.3	6.6	6.8	4.8	7.1	3.2	4.7
**174**	130.8	20.9	24.9	17.2	23.8	137.0	9.8	10.7	13.1	10.2	8.9	10.2	10.7
**182**	11.3	1.5	1.7	2.3	3.1	18.3	13.4	1.1	2.8	4.7	6.1	4.5	1.7
**186**	8.3	2.0	2.7	2.3	2.1	10.6	51.0	5.6	12.4	3.8	3.6	8.7	2.2
**187**	10.7	1.6	4.6	0.8	2.9	16.6	29.4	4.8	5.6	4.0	8.9	5.1	1.4
**197**	4.2	9.1	3.9	9.7	11.6	11.3	5.2	37.7	10.4	14.3	42.6	22.0	16.2
**198**	2.7	5.6	9.4	11.3	11.8	7.5	5.3	52.8	6.2	13.5	16.6	71.5	15.3
**201**	12.4	7.5	2.3	3.4	5.0	10.2	5.2	21.9	1.9	3.3	4.7	35.9	15.6
**209**	3.6	2.3	10.5	10.6	14.4	13.6	3.4	52.8	32.7	5.4	4.7	10.7	16.8
**212**	3.8	7.4	7.3	3.4	21.0	3.3	2.2	7.6	13.3	5.6	5.5	4.3	11.8
**215**	11.3	5.4	12.4	11.5	89.2	21.7	5.4	16.0	11.0	8.5	11.4	9.0	30.7
**216**	1.1	1.9	3.8	11.8	10.6	6.8	0.8	1.5	118.6	7.8	0.4	9.6	28.8
**219**	2.9	4.5	11.9	11.3	27.8	6.0	0.8	2.6	44.9	12.1	0.0	0.8	0.8
**220**	7.2	2.1	1.5	1.5	13.9	7.9	2.1	10.4	118.6	7.9	6.7	4.0	15.1
**225**	2.6	5.8	1.7	31.3	6.6	10.0	7.4	19.5	19.1	236.9	93.9	45.1	41.6
**238**	4.5	7.3	1.1	11.5	8.9	1.5	4.1	5.4	10.4	47.3	138.7	18.2	9.9
**239**	10.7	1.7	2.4	8.5	4.6	1.5	3.2	34.0	6.4	14.1	27.6	76.7	34.8
**241**	1.1	8.5	0.5	7.7	3.6	6.2	1.7	18.5	6.9	27.6	40.7	32.1	19.3
**250**	7.0	12.3	5.1	1.6	8.9	5.8	3.4	21.9	12.2	6.0	21.3	16.2	16.4
**253**	11.7	9.5	6.1	11.4	14.1	9.6	10.3	14.3	10.9	9.5	28.2	29.4	27.9
**262**	0.4	0.0	0.9	0.6	1.1	1.2	0.8	18.1	0.8	0.8	17.3	72.0	0.9
**265**	0.0	0.0	2.3	3.8	4.5	4.2	0.0	15.2	2.2	7.6	37.2	73.5	9.7
**268**	1.5	3.8	4.0	2.2	8.1	5.1	4.5	24.0	2.6	9.6	53.3	51.5	7.0
**276**	9.7	6.2	3.4	6.8	6.8	7.1	1.7	3.8	12.2	60.5	18.7	27.1	9.4
**277**	6.5	2.3	1.3	15.0	11.7	6.4	2.4	3.5	58.1	57.3	20.2	14.0	32.2
**283**	6.5	3.9	7.4	3.2	11.9	10.7	6.9	9.7	18.5	12.7	8.0	5.1	19.5
**285**	5.6	9.9	2.8	1.3	3.2	6.7	0.8	0.9	12.6	2.1	3.9	6.6	7.6
**287**	4.4	12.4	3.4	8.7	11.2	3.7	6.4	7.1	57.7	5.2	11.4	12.7	63.7
**288**	1.7	5.4	4.3	8.2	9.7	6.6	3.6	10.8	40.3	135.1	12.8	10.0	44.3
**291**	1.4	4.1	0.8	1.1	11.1	6.2	0.6	9.8	60.0	6.0	6.8	0.8	116.8
**292**	2.9	10.9	5.7	11.9	6.6	11.1	5.2	15.8	16.2	23.7	21.1	23.4	60.5
**293**	1.3	1.9	2.3	3.9	3.9	3.5	3.8	13.4	10.3	8.0	8.5	8.3	18.9
**294**	9.9	6.3	3.1	10.5	2.2	7.0	3.5	30.2	7.9	12.2	113.9	58.0	22.3
**300**	7.2	7.5	11.5	1.7	5.1	6.0	10.3	6.4	14.0	10.3	15.9	4.5	15.0
**323**	31.8	18.4	8.6	2.9	2.0	29.9	2.8	3.5	6.2	13.4	7.1	10.1	9.4
**333**	5.3	1.9	2.5	1.2	0.4	7.9	2.6	2.3	5.9	3.9	1.7	1.5	1.4
**337**	9.9	10.4	5.6	0.9	4.7	13.6	2.6	3.7	6.4	0.5	3.7	3.6	5.4
**343**	1.3	7.4	6.0	7.1	12.4	11.6	4.9	3.2	5.1	6.1	8.7	10.0	7.3
**357**	0.4	1.3	0.7	0.8	1.8	1.8	1.3	2.2	1.5	0.4	0.7	1.5	3.4
**361**	1.0	0.4	1.1	1.2	1.3	0.8	0.8	0.4	1.8	0.8	0.9	0.4	3.0
**367**	0.8	1.1	0.9	1.9	0.6	1.1	0.9	1.5	3.8	1.4	1.4	1.4	4.6

Table S3. ^{1}H-^{13}C average chemical shift perturbations from activated wildtype p38γ (Hz)

Residue	L77V	M109L	M112L	M120L	L159V	L174V	V186A	L198V	M216L	L225V	I238L	M239L	M291L
**16**	7.4	5.4	11.2	5.0	11.1	4.3	8.0	6.5	7.6	3.9	6.1	5.4	6.2
**23**	4.9	11.5	11.5	8.0	3.2	11.3	8.3	11.2	2.8	5.4	12.0	10.5	13.5
**26**	10.9	7.6	4.0	2.7	3.4	8.5	1.7	2.4	3.5	8.6	3.4	6.9	4.7
**30**	2.6	11.2	6.2	1.7	5.7	1.8	2.6	2.5	1.2	3.4	3.0	4.0	6.8
**33**	7.4	7.9	23.0	1.5	2.4	5.4	5.9	3.5	5.3	7.9	1.5	3.3	6.2
**41**	1.8	11.5	16.3	3.1	4.3	7.2	3.5	3.4	2.1	6.4	9.1	5.7	3.3
**45**	9.5	6.7	7.5	2.5	1.4	2.0	4.1	4.2	5.0	5.0	1.6	4.2	8.2
**53**	6.5	9.8	27.7	9.4	26.5	4.6	1.9	4.4	11.0	7.0	1.8	3.6	7.2
**55**	7.7	14.0	13.3	5.9	10.8	7.3	5.0	6.1	5.6	8.3	8.8	12.3	12.6
**58**	13.9	9.0	4.4	2.4	5.8	8.7	2.6	8.2	5.6	4.3	3.1	6.4	3.3
**66**	10.2	6.4	6.8	8.1	11.4	7.9	4.5	4.7	2.7	7.5	5.0	5.4	5.0
**75**	8.5	9.1	0.8	0.7	2.8	3.7	5.3	7.6	2.9	2.4	3.7	7.4	2.8
**77**	145.3	21.0	22.0	8.1	16.2	65.0	11.8	8.4	14.0	9.2	15.5	27.4	13.6
**78**	98.7	30.5	17.0	4.4	11.6	35.0	2.6	4.4	12.8	16.3	13.6	6.5	26.8
**81**	22.0	23.0	29.3	2.7	26.4	168.0	7.0	8.9	9.8	7.7	15.2	10.5	10.3
**86**	21.0	4.2	18.5	3.2	1.5	14.2	0.8	4.2	5.4	10.7	7.7	9.5	12.7
**87**	22.8	34.0	49.5	6.0	13.1	11.3	8.1	12.8	13.0	11.3	13.6	14.4	14.8
**89**	34.8	44.0	13.2	7.6	15.7	11.3	1.5	6.6	3.9	15.8	13.7	5.4	14.7
**90**	22.3	1.7	1.1	1.1	4.6	2.3	5.5	0.6	5.3	1.5	3.1	2.0	2.6
**109**	12.1	44.0	9.8	0.8	19.9	11.6	7.7	5.5	2.4	2.5	9.0	8.6	11.4
**112**	7.4	30.3	72.0	5.6	30.3	6.9	5.0	9.2	5.8	9.8	10.4	10.3	8.1
**116**	13.3	16.6	10.6	19.5	79.8	14.9	6.7	12.7	16.4	6.3	8.4	7.1	15.3
**119**	1.6	9.0	9.9	2.0	1.3	1.6	0.7	3.3	1.6	2.7	4.5	3.4	4.7
**120**	0.8	2.4	5.8	41.7	2.9	3.0	1.5	4.5	15.3	9.4	5.4	4.1	6.8
**125**	9.5	15.3	6.5	35.6	19.9	13.9	6.3	9.0	8.4	4.8	5.7	6.9	17.0
**130**	5.3	18.3	13.5	5.3	14.3	3.8	7.5	3.4	27.7	17.9	16.3	13.0	15.9
**134**	0.8	3.3	17.7	3.9	4.9	6.6	5.7	1.9	13.8	7.1	0.8	2.7	1.1
**137**	26.9	8.4	13.9	11.5	116.2	29.7	0.2	21.0	24.8	16.1	2.6	3.4	26.0
**141**	57.6	23.1	72.0	15.3	18.4	74.2	19.4	16.0	19.9	46.7	12.1	12.0	25.2
**144**	37.9	15.0	39.3	13.9	20.3	79.5	14.0	12.4	10.4	11.3	13.2	15.6	13.8
**149**	145.3	20.2	43.3	12.6	6.6	158.3	8.7	33.2	14.5	5.5	12.0	11.6	17.7
**150**	12.4	14.8	8.7	3.6	17.5	27.6	14.7	21.1	15.9	10.0	16.3	28.1	22.1
**154**	14.6	12.6	16.4	16.2	39.3	13.6	17.3	13.9	12.9	24.3	4.0	18.1	15.2
**159**	19.3	18.7	6.3	17.7	128.6	12.6	1.6	1.6	13.9	13.1	10.1	14.1	18.0
**161**	5.7	9.2	37.1	3.8	52.9	7.1	5.7	10.5	10.3	12.0	6.9	6.9	9.8
**167**	5.8	14.7	11.9	3.9	16.6	8.6	3.3	2.6	7.0	10.8	1.9	8.5	21.7
**169**	20.7	14.4	15.8	14.9	34.8	16.4	4.1	10.1	11.3	15.0	8.4	12.4	16.2
**170**	17.6	12.8	11.7	8.5	16.1	18.9	2.4	1.8	8.2	10.5	8.2	6.7	7.9
**174**	29.2	25.4	15.0	1.9	22.8	168.0	14.6	6.5	16.0	6.8	9.0	10.9	11.5
**182**	14.7	7.7	5.0	1.2	5.4	14.6	11.3	6.8	2.0	1.1	7.6	8.1	1.6
**186**	2.4	2.9	1.8	3.1	3.2	10.1	26.6	2.0	4.4	2.0	1.9	2.1	1.8
**187**	3.8	1.9	2.4	3.1	2.3	6.5	21.4	3.2	4.0	1.9	3.6	4.9	3.2
**197**	11.8	14.5	13.3	3.9	10.4	10.9	11.7	14.7	8.1	18.8	13.8	20.5	15.4
**198**	9.7	9.3	9.5	10.3	6.6	31.2	13.1	64.4	27.1	8.5	17.5	41.5	30.9
**201**	24.2	11.7	6.7	5.0	8.3	25.3	17.0	8.2	9.8	4.5	15.7	7.2	2.3
**209**	12.3	9.8	14.1	9.8	26.4	19.6	8.6	64.4	19.3	8.4	22.0	9.8	19.5
**212**	5.0	3.8	8.3	5.3	11.4	4.0	1.9	4.8	6.0	7.6	6.6	1.5	15.5
**215**	13.0	18.4	10.0	17.4	128.6	11.1	12.2	9.9	16.9	20.0	17.0	9.8	19.8
**216**	5.4	12.3	5.3	11.1	11.9	5.6	3.9	3.0	131.9	3.2	6.8	15.0	32.6
**219**	9.7	12.2	4.9	14.8	17.6	10.6	7.0	0.6	40.8	6.9	2.3	3.1	11.6
**220**	8.5	18.8	10.0	8.3	13.9	2.3	7.7	3.8	131.9	20.5	19.7	20.1	6.6
**225**	12.3	3.5	7.9	27.4	13.8	22.5	14.6	23.5	30.1	131.3	78.2	45.1	52.0
**238**	4.2	4.9	2.4	4.6	7.7	2.4	2.4	7.1	16.5	28.8	105.9	23.1	13.7
**239**	7.4	16.4	8.1	7.1	22.4	33.5	2.4	24.4	18.8	21.6	38.5	97.7	34.8
**241**	9.6	3.9	10.3	7.6	4.7	10.8	3.8	10.5	16.9	14.9	20.1	20.5	21.9
**250**	13.8	13.6	3.3	7.6	4.0	18.3	2.4	20.9	7.3	8.5	10.7	29.7	10.8
**253**	25.9	10.9	6.0	10.7	13.4	24.8	8.4	44.5	14.9	9.9	23.8	36.9	24.5
**262**	5.7	4.6	4.6	5.3	0.8	7.3	6.1	23.3	12.4	1.9	20.9	82.2	6.2
**265**	3.6	2.9	10.9	4.6	3.8	7.2	0.2	11.7	8.4	12.3	28.7	68.7	8.5
**268**	6.7	6.2	4.1	2.2	17.5	6.8	4.0	9.5	7.4	15.4	17.0	18.1	5.8
**276**	5.2	7.6	4.1	7.5	9.8	8.5	8.5	6.8	17.7	49.0	4.1	21.5	9.9
**277**	5.5	5.5	9.3	12.0	14.6	2.5	3.5	9.1	63.1	66.5	24.0	12.9	36.2
**283**	13.8	12.7	15.1	9.3	26.5	11.8	10.2	18.8	19.3	12.6	12.0	13.7	53.7
**285**	4.7	8.5	5.4	1.7	3.1	4.0	2.6	1.3	4.9	3.5	13.1	2.6	3.2
**287**	11.8	11.2	11.5	6.1	11.7	9.6	4.3	6.3	46.9	17.2	4.8	7.8	87.3
**288**	11.1	12.8	9.5	7.8	7.2	20.9	7.1	21.6	34.4	89.1	29.3	13.3	43.7
**291**	7.7	8.2	2.4	3.1	15.2	5.3	7.0	10.4	57.2	6.1	12.2	9.2	97.0
**292**	9.3	12.0	8.8	12.5	12.6	16.1	4.1	14.0	20.2	23.4	15.0	15.7	38.0
**293**	1.1	2.3	1.2	1.5	1.6	1.8	5.5	12.8	2.8	11.8	11.2	9.9	13.4
**294**	12.8	11.5	22.3	14.9	16.7	24.1	23.1	41.6	51.9	55.7	73.6	67.7	26.6
**300**	9.3	5.3	3.8	9.3	3.5	3.7	10.1	2.9	10.6	4.1	3.4	4.1	13.2
**323**	28.1	5.6	5.2	4.1	4.1	23.6	1.9	2.5	5.5	11.0	3.4	8.0	6.1
**333**	6.3	2.5	1.9	0.9	3.1	2.1	3.2	3.8	1.0	3.1	2.1	1.9	0.9
**337**	8.6	10.2	2.8	3.7	4.4	8.2	5.1	5.7	1.1	1.7	5.4	3.1	6.9
**343**	7.9	3.3	7.0	1.5	6.3	2.8	4.6	2.6	6.5	5.2	1.5	3.6	10.9
**357**	1.7	1.2	1.6	3.1	3.6	1.7	3.0	1.5	1.8	1.0	3.5	2.6	2.0
**361**	0.5	0.8	2.6	1.0	1.6	1.3	1.0	0.5	0.4	0.8	0.6	1.4	1.2
**367**	1.0	1.5	1.0	1.6	0.8	1.7	1.0	1.2	1.7	0.8	0.3	0.8	1.1

Table S4. ^{1}H-^{13}C average chemical shift perturbations from wildtype p38γ + BIRB796 (Hz)

Residue	L77V	M109L	M112L	M120L	L159V	L174V	V186A	L198V	M216L	L225V	M239L	M291L
**16**	8.3	21.3	9.8	2.8	3.4	12.0	2.4	7.0	0.4	9.7	4.5	9.1
**23**	2.4	43.1	15.1	4.0	5.8	30.7	4.2	2.1	5.7	5.4	3.8	3.6
**26**	1.2	30.3	3.9	2.1	5.4	21.1	4.5	3.0	2.6	3.8	2.7	4.1
**30**	0.8	12.0	12.8	3.0	6.3	18.2	3.4	2.7	1.4	7.9	2.5	4.3
**33**	1.7	15.3	9.3	4.3	0.8	4.2	2.4	4.3	0.2	5.7	2.4	3.6
**41**	7.1	12.3	7.4	4.1	8.0	11.1	2.4	2.5	9.3	5.9	2.0	5.3
**45**	5.8	3.7	4.0	3.7	5.2	9.1	0.0	2.4	2.6	6.0	5.6	3.1
**53**	6.8	10.4	17.6	8.4	11.8	8.1	2.5	7.3	6.2	6.1	6.1	6.2
**55**	5.4	20.7	12.8	0.2	1.7	12.1	3.6	1.7	6.4	9.2	3.5	3.6
**58**	12.6	45.3	5.3	4.0	3.3	44.2	10.7	2.5	2.4	3.1	5.7	5.8
**66**	5.3	7.2	9.8	3.5	7.0	44.7	9.0	2.2	3.4	6.6	4.8	4.1
**75**	0.0	18.8	1.0	0.0	1.6	2.6	2.6	1.1	1.1	3.1	1.0	0.8
**77**	86.6	86.3	3.8	3.2	11.6	44.3	8.9	2.7	8.5	4.1	3.7	6.6
**78**	11.5	59.9	10.6	4.1	5.7	12.1	2.4	3.1	4.1	6.2	7.9	7.5
**81**	76.5	11.4	1.5	0.0	1.5	5.3	1.1	3.2	0.8	0.8	1.7	4.2
**86**	5.9	7.5	2.3	2.3	6.1	27.7	9.9	2.7	4.5	4.3	6.8	6.4
**87**	3.6	197.6	78.6	6.6	15.8	44.6	5.3	9.8	11.6	9.9	7.6	10.7
**89**	3.3	11.5	7.6	2.3	6.8	73.6	6.0	0.0	1.9	4.5	5.9	5.7
**90**	7.5	6.0	14.1	3.0	11.5	37.7	10.1	1.1	0.4	12.7	2.4	1.7
**109**	8.4	253.6	65.8	7.1	6.8	5.3	0.9	3.5	0.9	3.1	1.4	3.1
**112**	7.0	74.3	78.6	9.6	8.9	12.6	5.0	5.9	6.8	2.6	1.2	6.9
**116**	12.1	11.5	17.9	29.2	77.8	8.5	3.9	9.9	8.9	8.9	10.0	6.0
**119**	3.4	8.4	29.2	7.2	19.4	2.9	8.5	2.0	6.4	1.9	5.7	5.8
**120**	1.1	1.1	1.9	97.0	10.7	3.5	0.6	4.6	12.3	3.8	0.4	1.1
**125**	0.0	1.5	6.1	24.0	21.6	3.3	5.3	4.2	11.0	0.0	10.7	8.6
**130**	7.7	9.4	5.3	4.9	8.9	3.8	14.7	5.9	14.3	8.9	5.0	15.9
**134**	10.8	1.1	2.3	7.4	14.6	7.2	7.7	5.7	16.6	5.4	7.7	15.8
**137**	11.1	4.8	12.3	6.1	106.9	7.1	0.6	12.4	24.8	11.5	10.3	34.0
**141**	13.4	12.0	12.4	24.4	17.7	10.8	6.6	58.2	13.6	11.7	29.4	42.1
**144**	26.0	12.3	11.9	12.1	31.9	11.7	3.9	12.8	12.8	9.9	14.1	10.7
**149**	86.6	36.9	3.8	5.3	9.4	34.6	7.1	2.7	7.1	4.8	3.3	2.4
**150**	12.6	6.1	2.3	5.6	11.6	7.9	1.5	17.4	1.3	9.7	14.0	13.3
**154**	4.4	9.0	18.4	25.9	37.7	11.5	0.2	11.5	2.3	3.8	1.9	17.0
**159**	13.4	23.1	11.4	12.6	227.8	5.5	7.0	6.2	13.4	8.1	9.7	7.5
**161**	7.0	38.0	38.4	12.2	32.6	7.6	2.9	10.2	40.2	3.9	11.2	3.5
**167**	4.4	26.2	4.9	16.2	204.9	8.1	7.5	11.6	17.4	8.8	14.0	7.6
**169**	66.1	26.7	6.0	27.4	19.9	9.3	4.5	23.4	6.5	9.3	13.3	17.1
**170**	7.7	16.2	23.5	51.9	27.2	11.3	3.4	6.3	7.0	4.3	2.9	10.5
**174**	9.3	18.0	8.9	6.4	11.9	97.2	10.8	5.3	10.7	9.1	4.6	10.1
**182**	1.4	4.5	2.4	1.4	1.4	8.1	5.3	5.6	1.4	1.1	1.4	1.4
**186**	0.5	3.0	0.8	0.5	3.3	6.8	22.4	3.7	3.7	2.4	1.5	2.3
**187**	1.7	2.5	4.7	0.6	3.7	6.9	18.0	7.2	6.8	1.1	4.5	6.5
**197**	6.1	10.8	4.5	4.6	9.2	2.7	4.1	23.1	6.6	4.6	40.5	13.1
**198**	4.7	8.8	4.5	9.5	9.9	3.6	2.4	58.2	6.1	10.4	16.5	21.8
**201**	2.0	6.2	3.8	11.3	9.1	3.2	2.3	43.9	0.0	5.9	13.4	11.4
**209**	9.1	15.7	13.4	0.0	24.6	0.0	2.4	30.5	27.8	7.9	15.9	17.8
**212**	5.7	4.7	1.8	2.7	7.9	5.0	2.2	6.8	6.3	7.2	6.8	12.6
**215**	0.8	12.1	11.9	10.5	123.8	2.1	8.6	12.4	15.0	9.4	14.0	13.5
**216**	1.5	7.1	4.8	10.9	6.8	1.6	0.2	5.3	131.5	12.1	11.1	28.9
**219**	3.1	0.8	3.1	14.4	14.7	3.0	1.5	2.5	29.4	7.8	0.2	8.1
**220**	3.5	7.8	7.1	5.2	6.6	6.6	15.8	0.0	131.5	7.7	7.0	11.0
**225**	3.0	7.3	4.4	29.4	5.0	12.6	20.5	19.1	9.0	242.0	31.1	40.3
**238**	1.9	2.1	0.0	8.1	10.0	7.2	1.5	12.0	12.1	55.7	24.3	11.8
**239**	1.5	2.3	3.4	7.7	9.8	9.5	3.8	55.8	9.8	15.2	82.9	33.1
**241**	0.8	0.0	1.0	3.1	5.7	6.4	3.5	12.2	8.6	48.3	28.8	15.0
**250**	4.2	8.7	5.4	5.3	4.5	4.0	3.9	13.4	1.7	12.7	15.1	18.6
**253**	2.3	8.4	9.7	11.0	8.4	7.4	10.2	24.9	2.3	5.6	62.4	24.0
**262**	0.0	0.0	0.0	1.3	6.2	3.5	0.0	19.7	4.2	1.7	82.9	7.0
**265**	4.6	8.1	6.7	3.0	9.0	4.8	7.1	29.2	0.8	15.8	61.6	14.6
**268**	9.5	5.7	3.8	6.6	10.7	9.6	4.9	11.9	5.6	7.4	38.8	12.0
**276**	1.9	1.1	0.0	6.8	7.5	2.9	0.8	5.4	17.6	42.3	19.8	15.5
**277**	5.4	9.7	7.7	10.2	9.6	4.4	6.4	6.6	49.4	23.4	14.1	36.7
**283**	1.7	5.6	6.6	7.2	11.4	1.9	2.4	7.3	24.2	6.2	5.1	11.4
**285**	8.3	0.1	1.6	2.5	5.3	1.6	0.7	0.1	2.0	9.4	0.6	7.4
**287**	7.9	5.9	7.8	4.5	2.6	3.1	6.1	9.6	49.9	4.7	13.4	75.0
**288**	3.8	5.8	5.3	6.8	5.5	6.4	10.3	12.4	44.1	139.4	9.5	42.9
**291**	5.9	3.2	3.9	4.0	11.9	2.4	3.4	8.0	54.4	5.9	5.7	80.0
**292**	3.7	10.1	9.7	9.8	15.2	7.5	3.5	6.8	24.2	18.2	27.5	26.3
**293**	2.5	4.1	1.1	0.8	4.2	2.2	1.2	9.2	5.9	11.4	7.8	17.1
**294**	3.7	6.4	6.6	12.5	10.2	6.9	11.7	20.8	7.3	12.0	54.9	21.3
**300**	4.7	9.4	3.7	1.6	3.9	5.0	13.0	5.3	5.4	7.1	4.0	10.0
**323**	6.2	7.4	9.4	6.0	10.9	15.6	6.2	14.1	6.0	6.8	8.5	5.3
**333**	13.0	8.9	3.6	3.4	1.6	7.4	8.0	5.7	5.9	6.7	1.2	4.1
**337**	2.0	22.3	2.7	6.5	0.7	6.0	1.4	5.4	5.2	4.4	2.4	10.2
**343**	14.4	25.6	5.3	2.7	7.3	22.6	1.7	7.7	2.8	0.9	5.2	5.4
**357**	0.0	0.0	0.7	0.0	0.0	3.0	0.8	3.0	0.0	5.7	0.8	2.1
**361**	0.0	1.5	0.6	0.0	0.5	0.4	0.5	0.0	0.4	3.3	0.0	5.8
**367**	1.9	3.5	2.0	0.0	0.9	0.0	0.0	1.1	0.0	6.5	1.3	3.0

Table S5. ^{1}H, ^{13}C methyl chemical shift assignments for wildtype p38γ + ATP

Res	ppm	Res	ppm	Res	ppm	Res	ppm	Res	ppm	Res	ppm
16.hg11	0.607	16.cg1	21.595	167.hd11	0.815	167.cd1	28.066	300.hg11	0.859	300.cg1	20.535
16.hg21	0.312	16.cg2	19.787	169.hd11	0.813	169.cd1	13.804	300.hg21	0.788	300.cg2	20.901
23.hg11	1.014	23.cg1	22.625	170.hd21	0.89	170.cd2	25.737	323.hg21	1.053	323.cg2	23.167
23.hg21	0.671	23.cg2	19.096	170.hd11	0.906	170.cd1	24.104	323.hg11	0.481	323.cg1	19.438
26.hg11	0.447	26.cg1	20.331	174.hd11	0.474	174.cd1	26.446	333.hg21	0.882	333.cg2	20.839
26.hg21	0.109	26.cg2	18.493	182.he1	1.964	182.ce	17.016	333.hg11	0.834	333.cg1	21.127
30.hd11	0.638	30.cd1	25.115	186.hg11	0.801	186.cg1	20.995	337.hd21	0.919	337.cd2	23.437
30.hd21	0.527	30.cd2	24.333	186.hg21	0.889	186.cg2	20.949	337.hd11	0.979	337.cd1	23.927
33.hg11	0.959	33.cg1	21.689	187.hg11	0.791	187.cg1	20.881	343.hg21	0.867	343.cg2	19.696
41.hg21	0.865	41.cg2	21.864	187.hg21	0.889	187.cg2	21.024	343.hg11	0.908	343.cg1	22.186
41.hg11	0.646	41.cg1	20.452	197.hd11	0.688	197.cd1	13.739	348.hg21	0.537	348.cg2	22.794
45.hg11	0.912	45.cg1	21.942	198.hd11	0.738	198.cd1	24.661	357.hd11	0.877	357.cd1	24.831
45.hg21	0.857	45.cg2	21.648	209.hd11	0.662	209.cd1	8.453	357.hd21	0.826	357.cd2	23.382
53.hg21	0.493	53.cg2	18.403	212.hg21	1.13	212.cg2	23.064	361.hg11	0.93	361.cg1	21.1
53.hg11	0.806	53.cg1	22.223	212.hg11	1.19	212.cg1	21.889	361.hg21	0.935	361.cg2	20.462
55.hd11	0.356	55.cd1	13.21	215.hd11	0.254	215.cd1	12.969	367.hd21	0.867	367.cd2	23.513
58.hd11	0.465	58.cd1	24.15	216.he1	1.531	216.ce	18.242	367.hd11	0.91	367.cd1	25.184
58.hd21	0.348	58.cd2	23.483	219.he1	1.994	219.ce	19.064				
66.hd21	0.865	66.cd2	24.332	220.hd11	0.716	220.cd1	14.804				
75.hd21	0.528	75.cd2	22.23	225.hd11	0.353	225.cd1	22.316				
75.hd11	0.302	75.cd1	24.227	225.hd21	0.18	225.cd2	27.666				
77.hd11	-0.091	77.cd1	22.68	238.hd11	0.575	238.cd1	13.726				
78.hd11	0.535	78.cd1	22.757	239.he1	2.001	239.ce	17.493				
81.he1	1.76	81.ce	18.492	241.hg11	0.751	241.cg1	21.792				
86.hg21	0.838	86.cg2	20.963	241.hg21	1.059	241.cg2	23.065				
87.hd11	1.047	87.cd1	15.374	250.hg21	1.027	250.cg2	23.359				
89.hd21	0.726	89.cd2	25.134	250.hg11	0.934	250.cg1	21.94				
90.hd11	0.915	90.cd1	24.65	253.hd11	0.683	253.cd1	23.14				
92.hg11	0.905	92.cg1	20.409	262.he1	2.064	262.ce	17.547				
109.he1	1.923	109.ce	17.239	265.hd21	0.692	265.cd2	22.796				
112.he1	1.694	112.ce	17.04	268.hd21	0.752	268.cd2	25.798				
116.hd11	0.943	116.cd1	24.996	276.hd11	0.585	276.cd1	13.768				
116.hd21	0.759	116.cd2	24.674	277.hd11	0.745	277.cd1	27.441				
119.hd11	0.641	119.cd1	25.069	277.hd21	0.654	277.cd2	23.97				
119.hd21	0.528	119.cd2	24.25	283.hd21	0.359	283.cd2	24.901				
120.he1	1.891	120.ce	16.014	285.hg21	0.971	285.cg2	23.994				
125.hd11	0.796	125.cd1	26.367	285.hg11	0.959	285.cg1	22.35				
130.hd11	0.707	130.cd1	13.778	287.hd11	0.728	287.cd1	22.002				
134.hg21	0.865	134.cg2	19.918	287.hd21	0.493	287.cd2	27.596				
137.he1	1.779	137.ce	17.303	288.hd11	0.387	288.cd1	26.187				
141.hd11	0.604	141.cd1	25.138	288.hd21	-0.205	288.cd2	23.093				
144.hd11	0.709	144.cd1	14.412	291.he1	1.907	291.ce	18.247				
149.hd11	0.551	149.cd1	11.814	292.hd21	0.738	292.cd2	21.909				
154.hd11	0.782	154.cd1	26.89	292.hd11	0.657	292.cd1	26.69				
159.hd11	0.792	159.cd1	28.067	293.hg11	1.076	293.cg1	22.432				
161.hg11	0.814	161.cg1	22.477	293.hg21	0.901	293.cg2	20.168				
161.hg21	0.628	161.cg2	20.796	294.hd11	0.598	294.cd1	24.564				
167.hd21	0.512	167.cd2	24.475	294.hd21	0.363	294.cd2	21.573				

Table S6. ^{1}H, ^{13}C methyl chemical shift assignments for activated wildtype p38γ

Res	ppm	Res	ppm	Res	ppm	Res	ppm	Res	ppm	Res	ppm
**16.hg11**	0.622	**16.cg1**	21.416	**167.hd11**	0.825	**167.cd1**	27.88	**300.hg11**	0.863	**300.cg1**	20.341
**16.hg21**	0.333	**16.cg2**	19.724	**169.hd11**	0.833	**169.cd1**	13.843	**300.hg21**	0.804	**300.cg2**	20.929
**23.hg11**	1.029	**23.cg1**	22.578	**170.hd21**	0.881	**170.cd2**	25.634	**323.hg21**	1.067	**323.cg2**	23.072
**23.hg21**	0.697	**23.cg2**	18.953	**170.hd11**	0.855	**170.cd1**	23.718	**323.hg11**	0.466	**323.cg1**	19.246
**26.hg11**	0.473	**26.cg1**	20.304	**174.hd11**	0.533	**174.cd1**	26.55	**333.hg21**	0.889	**333.cg2**	20.78
**26.hg21**	0.103	**26.cg2**	18.295	**182.he1**	1.979	**182.ce**	16.911	**333.hg11**	0.845	**333.cg1**	21.024
**30.hd11**	0.657	**30.cd1**	25.012	**186.hg11**	0.815	**186.cg1**	20.962	**337.hd21**	0.921	**337.cd2**	23.239
**30.hd21**	0.54	**30.cd2**	24.239	**186.hg21**	0.897	**186.cg2**	20.844	**337.hd11**	0.983	**337.cd1**	23.838
**33.hg11**	0.982	**33.cg1**	21.026	**187.hg11**	0.798	**187.cg1**	20.838	**343.hg21**	0.875	**343.cg2**	19.761
**41.hg21**	0.887	**41.cg2**	21.656	**187.hg21**	0.901	**187.cg2**	20.896	**343.hg11**	0.907	**343.cg1**	21.75
**41.hg11**	0.673	**41.cg1**	20.387	**197.hd11**	0.704	**197.cd1**	13.676	**348.hg21**	0.541	**348.cg2**	22.376
**45.hg11**	0.919	**45.cg1**	21.767	**198.hd11**	0.725	**198.cd1**	24.382	**357.hd11**	0.889	**357.cd1**	24.723
**45.hg21**	0.893	**45.cg2**	21.437	**209.hd11**	0.669	**209.cd1**	8.269	**357.hd21**	0.836	**357.cd2**	23.285
**53.hg21**	0.496	**53.cg2**	18.318	**212.hg21**	1.151	**212.cg2**	22.951	**361.hg11**	0.937	**361.cg1**	20.98
**53.hg11**	0.835	**53.cg1**	22.366	**212.hg11**	1.199	**212.cg1**	21.732	**361.hg21**	0.944	**361.cg2**	20.338
**55.hd11**	0.388	**55.cd1**	13.09	**215.hd11**	0.29	**215.cd1**	13.087	**367.hd21**	0.879	**367.cd2**	23.425
**58.hd11**	0.482	**58.cd1**	23.931	**216.he1**	1.532	**216.ce**	18.146	**367.hd11**	0.92	**367.cd1**	25.016
**58.hd21**	0.346	**58.cd2**	23.486	**219.he1**	2.017	**219.ce**	18.906				
**66.hd21**	0.869	**66.cd2**	24.262	**220.hd11**	0.731	**220.cd1**	14.641				
**75.hd21**	0.537	**75.cd2**	22.082	**225.hd11**	0.366	**225.cd1**	22.353				
**75.hd11**	0.32	**75.cd1**	24.177	**225.hd21**	0.192	**225.cd2**	27.319				
**77.hd11**	0.018	**77.cd1**	22.707	**238.hd11**	0.586	**238.cd1**	13.575				
**78.hd11**	0.546	**78.cd1**	22.461	**239.he1**	2.018	**239.ce**	17.339				
**81.he1**	1.844	**81.ce**	18.536	**241.hg11**	0.77	**241.cg1**	21.664				
**86.hg21**	0.834	**86.cg2**	21.061	**241.hg21**	1.06	**241.cg2**	22.98				
**87.hd11**	1.025	**87.cd1**	14.907	**250.hg21**	1.033	**250.cg2**	23.294				
**89.hd21**	0.724	**89.cd2**	25.111	**250.hg11**	0.943	**250.cg1**	21.82				
**90.hd11**	0.925	**90.cd1**	24.445	**253.hd11**	0.693	**253.cd1**	23.119				
**92.hg11**	0.908	**92.cg1**	20.318	**262.he1**	2.079	**262.ce**	17.448				
**109.he1**	1.915	**109.ce**	17.037	**265.hd21**	0.708	**265.cd2**	22.72				
**112.he1**	1.742	**112.ce**	17.608	**268.hd21**	0.774	**268.cd2**	25.675				
**116.hd11**	0.96	**116.cd1**	24.914	**276.hd11**	0.604	**276.cd1**	13.53				
**116.hd21**	0.771	**116.cd2**	25.011	**277.hd11**	0.759	**277.cd1**	27.256				
**119.hd11**	0.654	**119.cd1**	24.873	**277.hd21**	0.666	**277.cd2**	23.863				
**119.hd21**	0.542	**119.cd2**	24.152	**283.hd21**	0.386	**283.cd2**	24.643				
**120.he1**	1.914	**120.ce**	15.936	**285.hg21**	0.98	**285.cg2**	23.906				
**125.hd11**	0.817	**125.cd1**	26.298	**285.hg11**	0.954	**285.cg1**	22.3				
**130.hd11**	0.721	**130.cd1**	13.704	**287.hd11**	0.743	**287.cd1**	21.965				
**134.hg21**	0.877	**134.cg2**	19.971	**287.hd21**	0.501	**287.cd2**	27.413				
**137.he1**	1.798	**137.ce**	17.39	**288.hd11**	0.401	**288.cd1**	26.088				
**141.hd11**	0.529	**141.cd1**	25.225	**288.hd21**	-0.188	**288.cd2**	22.998				
**144.hd11**	0.665	**144.cd1**	14.371	**291.he1**	1.915	**291.ce**	18.19				
**149.hd11**	0.592	**149.cd1**	11.514	**292.hd21**	0.758	**292.cd2**	21.841				
**154.hd11**	0.805	**154.cd1**	26.715	**292.hd11**	0.664	**292.cd1**	26.815				
**159.hd11**	0.819	**159.cd1**	27.719	**293.hg11**	1.085	**293.cg1**	22.336				
**161.hg11**	0.837	**161.cg1**	22.49	**293.hg21**	0.901	**293.cg2**	20.102				
**161.hg21**	0.623	**161.cg2**	20.772	**294.hd11**	0.606	**294.cd1**	24.415				
**167.hd21**	0.557	**167.cd2**	24.391	**294.hd21**	0.377	**294.cd2**	21.653				

*Underline indicates methyls that are not stereospecifically assigned.

Table S7. ^{1}H, ^{13}C methyl chemical shift assignments for wildtype p38γ + BIRB796

Res	ppm	Res	ppm	Res	ppm	Res	ppm	Res	ppm	Res	ppm
**16.hg11**	0.647	**16.cg1**	21.321	**170.hd21**	0.815	**170.cd2**	25.297	**333.hg21**	0.881	**333.cg2**	20.757
**16.hg21**	0.386	**16.cg2**	19.948	**170.hd11**	0.831	**170.cd1**	23.686	**333.hg11**	0.849	**333.cg1**	20.957
**23.hg11**	1.049	**23.cg1**	21.834	**174.hd11**	0.579	**174.cd1**	26.009	**337.hd21**	0.928	**337.cd2**	23.421
**23.hg21**	0.724	**23.cg2**	18.854	**182.he1**	2.032	**182.ce**	16.857	**337.hd11**	0.966	**337.cd1**	23.841
**26.hg11**	0.535	**26.cg1**	20.555	**186.hg11**	0.755	**186.cg1**	20.911	**343.hg21**	0.893	**343.cg2**	19.425
**26.hg21**	0.028	**26.cg2**	17.799	**186.hg21**	0.874	**186.cg2**	20.74	**343.hg11**	0.904	**343.cg1**	21.931
**30.hd11**	0.653	**30.cd1**	25.132	**187.hg11**	0.748	**187.cg1**	21.022	**348.hg21**	0.563	**348.cg2**	22.502
**30.hd21**	0.535	**30.cd2**	24.161	**187.hg21**	0.866	**187.cg2**	20.709	**357.hd11**	0.88	**357.cd1**	24.724
**33.hg11**	0.999	**33.cg1**	21.309	**197.hd11**	0.697	**197.cd1**	13.657	**357.hd21**	0.826	**357.cd2**	23.262
**41.hg21**	0.853	**41.cg2**	21.573	**198.hd11**	0.724	**198.cd1**	24.536	**361.hg11**	0.93	**361.cg1**	21.101
**41.hg11**	0.657	**41.cg1**	20.352	**209.hd11**	0.662	**209.cd1**	8.192	**361.hg21**	0.929	**361.cg2**	20.363
**45.hg11**	0.906	**45.cg1**	21.805	**212.hg21**	1.154	**212.cg2**	22.902	**367.hd21**	0.86	**367.cd2**	23.373
**45.hg21**	0.875	**45.cg2**	21.665	**212.hg11**	1.19	**212.cg1**	21.788	**367.hd11**	0.907	**367.cd1**	25.045
**53.hg21**	0.498	**53.cg2**	18.426	**215.hd11**	0.291	**215.cd1**	13.11				
**53.hg11**	0.858	**53.cg1**	22.188	**216.he1**	1.514	**216.ce**	18.146				
**55.hd11**	0.379	**55.cd1**	12.872	**219.he1**	2.009	**219.ce**	18.966				
**58.hd11**	0.452	**58.cd1**	24.133	**220.hd11**	0.724	**220.cd1**	14.736				
**58.hd21**	0.297	**58.cd2**	23.56	**225.hd11**	0.348	**225.cd1**	22.357				
**66.hd21**	0.838	**66.cd2**	24.557	**225.hd21**	0.187	**225.cd2**	27.4				
**75.hd21**	0.52	**75.cd2**	21.878	**238.hd11**	0.57	**238.cd1**	13.613				
**75.hd11**	0.303	**75.cd1**	24.31	**239.he1**	2.045	**239.ce**	17.328				
**77.hd11**	0.364	**77.cd1**	24.271	**241.hg11**	0.756	**241.cg1**	21.651				
**78.hd11**	0.501	**78.cd1**	23.158	**241.hg21**	1.05	**241.cg2**	22.923				
**81.he1**	2.08	**81.ce**	19.084	**250.hg21**	1.005	**250.cg2**	23.329				
**86.hg21**	0.817	**86.cg2**	20.974	**250.hg11**	0.935	**250.cg1**	21.881				
**87.hd11**	1.194	**87.cd1**	15.333	**253.hd11**	0.668	**253.cd1**	23.252				
**89.hd21**	0.724	**89.cd2**	24.954	**262.he1**	2.064	**262.ce**	17.437				
**90.hd11**	0.92	**90.cd1**	24.245	**265.hd21**	0.691	**265.cd2**	22.622				
**92.hg11**	0.904	**92.cg1**	20.321	**268.hd21**	0.772	**268.cd2**	25.765				
**109.he1**	1.618	**109.ce**	14.923	**276.hd11**	0.566	**276.cd1**	13.589				
**112.he1**	1.712	**112.ce**	17.005	**277.hd11**	0.749	**277.cd1**	27.349				
**116.hd11**	0.955	**116.cd1**	24.697	**277.hd21**	0.657	**277.cd2**	23.9				
**116.hd21**	0.767	**116.cd2**	24.792	**283.hd21**	0.364	**283.cd2**	24.79				
**119.hd11**	0.646	**119.cd1**	24.922	**285.hg21**	0.966	**285.cg2**	23.961				
**119.hd21**	0.551	**119.cd2**	24.303	**285.hg11**	0.974	**285.cg1**	21.785				
**120.he1**	1.907	**120.ce**	15.917	**287.hd11**	0.738	**287.cd1**	21.883				
**125.hd11**	0.808	**125.cd1**	26.234	**287.hd21**	0.489	**287.cd2**	27.368				
**130.hd11**	0.721	**130.cd1**	13.664	**288.hd11**	0.4	**288.cd1**	26.079				
**134.hg21**	0.883	**134.cg2**	19.576	**288.hd21**	-0.204	**288.cd2**	22.971				
**137.he1**	1.797	**137.ce**	17.384	**291.he1**	1.901	**291.ce**	18.183				
**141.hd11**	0.45	**141.cd1**	25.099	**292.hd21**	0.747	**292.cd2**	21.785				
**144.hd11**	0.632	**144.cd1**	13.736	**292.hd11**	0.673	**292.cd1**	26.557				
**149.hd11**	0.625	**149.cd1**	13.064	**293.hg11**	1.068	**293.cg1**	22.421				
**154.hd11**	0.771	**154.cd1**	26.641	**293.hg21**	0.903	**293.cg2**	20.096				
**159.hd11**	0.798	**159.cd1**	27.793	**294.hd11**	0.588	**294.cd1**	24.429				
**161.hg11**	0.743	**161.cg1**	22.261	**294.hd21**	0.333	**294.cd2**	21.366				
**161.hg21**	0.672	**161.cg2**	21.21	**300.hg11**	0.868	**300.cg1**	20.246				
**167.hd21**	0.582	**167.cd2**	24.869	**300.hg21**	0.793	**300.cg2**	20.97				
**167.hd11**	0.821	**167.cd1**	27.868	**323.hg21**	1.045	**323.cg2**	22.956				
**169.hd11**	0.758	**169.cd1**	13.656	**323.hg11**	0.546	**323.cg1**	19.454				

*Underline indicates methyls that are not stereospecifically assigned.

***For Tables S8, S10, S12, S13: Pairwise flow is given as X X Y (pairwise between X and Y residues), three-residue flow is given as X Y Z.**

Table S8. Methyl 3-residue flow for apo inactive p38γ

First Res	Second Res	Third Res	Scaled Flow
V16	V16	V26	171.4
V16	V16	L78	134.3
V16	V16	M112	181.9
V16	V16	L116	60.2
V16	V16	L119	40.6
V16	V16	V343	50.1
V16	V26	L78	3552.0
V16	L78	V26	3333.8
V16	L78	V343	4521.1
V16	V343	L78	3591.7
V23	V23	L119	26.7
V26	V26	V16	171.4
V26	V26	V53	29.5
V26	V26	L58	87.9
V26	V26	L78	787.4
V26	V26	M81	67.5
V26	V26	L89	323.1
V26	V26	M109	416.8
V26	V26	M112	542.7
V26	V26	L116	67.5
V26	V26	L119	30.3
V26	V26	I149	22.3
V26	V26	L170	591.5
V26	V26	L337	75.4
V26	V26	V343	289.0
V26	V53	L116	2982.6
V26	V53	L170	2229.5
V26	L58	L89	2931.3
V26	L58	L337	3493.2
V26	L78	M81	5908.9
V26	L78	L89	5954.4
V26	L78	I149	6804.4
V26	L78	V343	5334.6
V26	M81	L78	2865.0
V26	M81	M109	2501.7
V26	M81	I149	3250.3
V26	L89	L58	4154.1
V26	L89	L78	4349.0
V26	L89	M109	4010.6
V26	L89	L170	3602.3
V26	M109	M81	4088.1
V26	M109	L89	4350.7
V26	M109	V343	3798.1
V26	L116	V53	3556.2
V26	I149	L78	2618.6
V26	I149	M81	2579.8
V26	L170	V53	4908.4
V26	L170	L89	4415.5
V26	L337	L58	3370.7
V26	V343	L78	3766.0
V26	V343	M109	3384.2
L30	L30	V33	74.0
L30	L30	L58	612.1
L30	L30	L75	471.9
L30	L30	L78	22.3
L30	L30	I87	268.0
L30	L30	L89	86.1
L30	L30	L119	521.2
L30	L30	V323	78.8
L30	L58	L78	4755.5
L30	L58	L89	4751.3
L30	L78	L58	2018.5
L30	L78	L89	3581.8
L30	I87	L89	3816.5
L30	I87	V323	4226.8
L30	L89	L58	2687.1
L30	L89	L78	4772.4
L30	L89	I87	2834.9
L30	V323	I87	3076.3
V33	L30	L119	2960.1
V33	V33	L30	74.0
V33	V33	V41	34.7
V33	V33	V53	603.5
V33	V33	L119	100.7

First Res	Second Res	Third Res	Scaled Flow
V33	V33	M120	46.3
V33	V33	V161	46.8
V33	V33	I169	37.8
V33	V33	L170	109.3
V33	V33	I220	32.2
V33	V41	I169	3011.9
V33	V53	V161	7568.1
V33	L119	L30	3184.6
V33	V161	V53	3755.5
V33	I169	V41	3065.2
V41	V33	V53	2638.8
V41	V41	V33	34.7
V41	V41	V53	181.9
V41	V41	I55	67.1
V41	V41	L58	347.8
V41	V41	I87	39.5
V41	V41	L89	164.6
V41	V41	M109	87.9
V41	V41	L116	22.3
V41	V41	L119	79.9
V41	V41	M137	25.3
V41	V41	L141	171.4
V41	V41	V161	89.9
V41	V41	I169	633.3
V41	V41	L170	37.7
V41	V41	V323	31.0
V41	V53	V33	3881.9
V41	V53	L116	4308.2
V41	V53	V161	5420.8
V41	V53	L170	3750.5
V41	L58	I87	4559.8
V41	L58	L89	3869.1
V41	L58	V323	4030.6
V41	I87	L58	2634.9
V41	I87	L89	2730.7
V41	I87	M109	2710.8
V41	I87	V323	3339.3
V41	L89	L58	3119.5
V41	L89	I87	3810.0
V41	L89	M109	4301.6
V41	L89	L141	3570.3

First Res	Second Res	Third Res	Scaled Flow
V41	L89	L170	4457.9
V41	M109	I87	3235.3
V41	M109	L89	3679.4
V41	M109	L170	3500.4
V41	L116	V53	2679.1
V41	L116	V161	2561.4
V41	M137	L141	2567.1
V41	L141	L89	3608.5
V41	L141	M137	3961.8
V41	L141	I169	3439.7
V41	L141	L170	3584.4
V41	V161	V53	4537.8
V41	V161	L116	3447.9
V41	I169	L141	5186.8
V41	L170	V53	2591.1
V41	L170	L89	3163.1
V41	L170	M109	2903.7
V41	L170	L141	2516.4
V41	V323	L58	2215.3
V41	V323	I87	3176.1
V45	V45	L119	273.9
V45	V45	V134	42.6
V45	V45	L357	63.2
V53	V26	M112	2558.7
V53	V26	L170	2313.7
V53	V41	I169	3335.9
V53	V53	V26	29.5
V53	V53	V33	603.5
V53	V53	V41	181.9
V53	V53	M112	161.3
V53	V53	L116	827.3
V53	V53	L119	72.4
V53	V53	M137	164.6
V53	V53	L154	168.0
V53	V53	L159	480.3
V53	V53	V161	4391.7
V53	V53	I169	155.0
V53	V53	L170	533.4
V53	V53	I215	854.9
V53	M112	V26	3771.2
V53	M137	L159	3024.5

First Res	Second Res	Third Res	Scaled Flow
V53	L159	M137	4172.5
V53	I169	V41	3196.8
V53	L170	V26	4906.4
I55	I55	V41	67.1
I55	I55	L58	80.2
L58	V26	L78	2858.3
L58	V26	L170	3311.5
L58	L58	V26	87.9
L58	L58	L30	612.1
L58	L58	V41	347.8
L58	L58	I55	80.2
L58	L58	L66	39.5
L58	L58	L75	402.1
L58	L58	L77	221.3
L58	L58	L78	394.9
L58	L58	M81	164.6
L58	L58	I87	749.2
L58	L58	L89	787.4
L58	L58	M109	374.1
L58	L58	L119	203.4
L58	L58	L141	200.8
L58	L58	I149	26.7
L58	L58	I169	193.0
L58	L58	L170	52.5
L58	L58	L174	64.5
L58	L58	V323	394.9
L58	L58	L337	883.2
L58	L58	V343	106.7
L58	L77	L78	4275.9
L58	L77	M81	4983.9
L58	L77	L141	3384.4
L58	L77	I149	5231.8
L58	L77	L174	4563.2
L58	L78	V26	4317.0
L58	L78	L77	5095.1
L58	L78	M81	4479.5
L58	L78	L89	4490.0
L58	L78	I149	5549.8
L58	L78	V343	5222.6
L58	M81	L77	4597.0
L58	M81	L78	3467.4

First Res	Second Res	Third Res	Scaled Flow
L58	M81	I149	3705.9
L58	L89	L78	5768.1
L58	L89	M109	4794.9
L58	L89	L141	4585.9
L58	L89	L170	6165.0
L58	M109	L89	3667.5
L58	M109	L170	4382.7
L58	M109	V343	4325.4
L58	L141	L77	3293.6
L58	L141	L89	2915.4
L58	L141	I169	4096.9
L58	L141	L170	3555.8
L58	I149	L77	3192.2
L58	I149	L78	2841.7
L58	I149	M81	2451.5
L58	I149	L174	2488.3
L58	I149	V323	2517.1
L58	I169	L141	4052.9
L58	L170	V26	2947.0
L58	L170	L89	2827.7
L58	L170	M109	2628.2
L58	L170	L141	2565.4
L58	L174	L77	3352.8
L58	L174	I149	2996.5
L58	V323	I149	4915.7
L58	V343	L78	3621.7
L58	V343	M109	3052.6
L66	L66	L58	39.5
L66	L66	L77	82.4
L66	L66	M81	46.7
L66	L66	L90	58.9
L66	L66	M182	300.0
L66	L66	V186	28.0
L66	L66	V187	80.6
L66	L66	L357	120.2
L66	L77	M81	4587.7
L66	M81	L77	4042.9
L66	M182	V187	4539.2
L66	V186	V187	2964.4
L66	V187	M182	3210.5
L66	V187	V186	3718.3

First Res	Second Res	Third Res	Scaled Flow
L75	L58	L89	4100.7
L75	L75	L30	471.9
L75	L75	L58	402.1
L75	L75	L77	148.8
L75	L75	L78	221.3
L75	L75	M81	64.5
L75	L75	I87	289.0
L75	L75	L89	131.6
L75	L75	M109	37.7
L75	L75	I149	58.9
L75	L75	L170	26.0
L75	L75	V323	126.2
L75	L77	L78	4273.5
L75	L77	M81	4995.4
L75	L77	I149	4563.4
L75	L78	L77	4757.4
L75	L78	M81	4456.7
L75	L78	L89	5026.9
L75	L78	M109	4187.4
L75	L78	I149	4570.5
L75	L78	L170	3918.5
L75	M81	L77	4086.1
L75	M81	L78	3274.6
L75	M81	M109	3181.3
L75	M81	I149	2990.1
L75	M81	L170	3024.8
L75	I87	L89	3655.7
L75	I87	M109	3967.2
L75	I87	V323	4054.7
L75	L89	L58	2977.8
L75	L89	L78	4375.4
L75	L89	I87	2944.4
L75	L89	M109	4424.7
L75	L89	L170	4394.6
L75	M109	L78	2740.5
L75	M109	M81	2833.7
L75	M109	I87	2402.6
L75	M109	L89	3327.0
L75	M109	L170	3161.2
L75	I149	L77	3658.0
L75	I149	L78	3291.0

First Res	Second Res	Third Res	Scaled Flow
L75	I149	M81	2930.3
L75	I149	V323	3069.2
L75	L170	L78	2379.0
L75	L170	M81	2499.3
L75	L170	L89	3065.2
L75	L170	M109	2932.4
L75	V323	I87	3231.8
L75	V323	I149	3671.5
L77	L58	I87	3960.8
L77	L58	L89	3553.2
L77	L58	L337	4207.2
L77	L77	L58	221.3
L77	L77	L66	82.4
L77	L77	L75	148.8
L77	L77	L78	2422.5
L77	L77	M81	3124.4
L77	L77	V86	127.9
L77	L77	I87	66.0
L77	L77	L89	181.9
L77	L77	L90	35.0
L77	L77	M109	60.2
L77	L77	L141	689.1
L77	L77	I144	221.3
L77	L77	I149	1918.7
L77	L77	I169	37.7
L77	L77	L170	52.5
L77	L77	L174	1397.8
L77	L77	M182	181.9
L77	L77	V186	64.5
L77	L77	V187	89.9
L77	L77	V323	243.7
L77	L77	L337	52.5
L77	L77	V343	95.9
L77	L78	L89	9852.7
L77	L78	M109	7892.5
L77	L78	L170	6965.0
L77	L78	V343	9640.2
L77	M81	M109	8115.1
L77	I87	L58	2924.9
L77	I87	L89	2872.6
L77	I87	M109	2993.4

First Res	Second Res	Third Res	Scaled Flow
L77	I87	V323	3040.9
L77	L89	L58	3366.0
L77	L89	L78	3362.2
L77	L89	I87	3685.0
L77	L89	M109	4526.5
L77	L89	L141	2960.2
L77	L89	L170	4420.4
L77	L89	V343	3493.5
L77	M109	L78	2057.5
L77	M109	M81	1732.9
L77	M109	I87	2933.5
L77	M109	L89	3457.9
L77	M109	L170	3222.6
L77	M109	V343	3263.8
L77	L141	L89	4536.8
L77	L141	I144	4687.8
L77	L141	I169	6380.7
L77	I144	L141	3228.8
L77	I169	L141	2876.3
L77	L170	L78	1761.6
L77	L170	L89	3276.3
L77	L170	M109	3126.6
L77	M182	V187	4095.7
L77	V186	V187	3290.5
L77	V187	M182	3428.6
L77	V187	V186	3551.4
L77	V323	I87	4232.4
L77	L337	L58	2954.0
L77	V343	L78	2796.5
L77	V343	L89	2969.8
L77	V343	M109	3632.0
L78	L30	L58	2346.8
L78	L30	L75	2341.8
L78	L58	L30	4746.8
L78	L58	L337	3972.1
L78	L75	L30	3975.2
L78	L77	M81	6132.9
L78	L77	L174	9326.3
L78	L78	V16	134.3
L78	L78	V26	787.4
L78	L78	L30	22.3
L78	L78	L58	394.9
L78	L78	L75	221.3
L78	L78	L77	2422.5
L78	L78	M81	1254.2
L78	L78	V86	23.0
L78	L78	I87	225.6
L78	L78	L89	2954.7
L78	L78	M109	749.2
L78	L78	M112	128.9
L78	L78	L116	89.9
L78	L78	L141	394.9
L78	L78	I144	39.5
L78	L78	I149	1355.4
L78	L78	L170	471.9
L78	L78	L174	69.0
L78	L78	M182	185.6
L78	L78	V323	118.6
L78	L78	L337	196.9
L78	L78	V343	1890.5
L78	M81	L77	4163.3
L78	I87	V323	3916.1
L78	M112	L170	2797.0
L78	L141	I144	5175.1
L78	I144	L141	2871.0
L78	I144	L174	2937.0
L78	I149	V323	5523.8
L78	L170	M112	4085.6
L78	L174	L77	2506.2
L78	L174	I144	3314.3
L78	L174	V323	2777.8
L78	V323	I87	3302.8
L78	V323	I149	2390.8
L78	V323	L174	3157.7
L78	L337	L58	3226.5
M81	V26	L78	2362.6
M81	V26	L170	2617.8
M81	L58	I87	3656.5
M81	L58	L89	3497.2
M81	L77	L78	5844.2
M81	L77	I149	7648.3
M81	L77	L174	7813.7

First Res	Second Res	Third Res	Scaled Flow
M81	L78	V26	5998.5
M81	L78	L77	3249.8
M81	L78	L89	7695.5
M81	L78	I149	4806.7
M81	L78	V343	6962.0
M81	M81	V26	67.5
M81	M81	L58	164.6
M81	M81	L66	46.7
M81	M81	L75	64.5
M81	M81	L77	3124.4
M81	M81	L78	1254.2
M81	M81	I87	87.9
M81	M81	L89	145.8
M81	M81	M109	533.4
M81	M81	L116	21.7
M81	M81	L141	463.7
M81	M81	I144	95.9
M81	M81	I149	471.9
M81	M81	I169	73.8
M81	M81	L170	360.7
M81	M81	L174	283.6
M81	M81	M182	69.0
M81	M81	V323	128.9
M81	M81	V343	126.2
M81	I87	L58	3127.7
M81	I87	L89	3047.5
M81	I87	V323	3363.3
M81	L89	L58	3387.9
M81	L89	L78	3648.8
M81	L89	I87	3451.5
M81	L89	M109	3467.5
M81	L89	L141	3083.3
M81	L89	L170	3494.3
M81	L89	V343	3274.6
M81	M109	L89	5122.7
M81	M109	V343	4537.8
M81	L141	L89	4338.0
M81	L141	I144	4865.9
M81	L141	I169	5366.2
M81	I144	L141	3117.0
M81	I144	L174	2849.0

First Res	Second Res	Third Res	Scaled Flow
M81	I149	L77	2854.3
M81	I149	L78	3226.0
M81	I149	V323	4220.2
M81	I169	L141	3233.1
M81	L170	V26	4081.8
M81	L170	L89	4525.8
M81	L174	L77	2477.7
M81	L174	I144	3801.7
M81	V323	I87	3691.0
M81	V323	I149	2889.1
M81	V343	L78	3182.1
M81	V343	L89	3156.6
M81	V343	M109	2960.9
V86	L77	L78	4948.0
V86	L77	I149	4493.9
V86	L77	L174	4339.9
V86	L78	L77	3391.7
V86	L78	I149	3219.6
V86	V86	L77	127.9
V86	V86	L78	23.0
V86	V86	I149	50.2
V86	V86	L174	34.3
V86	V86	V186	63.2
V86	V86	V323	57.7
V86	V86	L357	119.9
V86	I149	L77	3616.6
V86	I149	L78	3780.1
V86	I149	L174	2836.2
V86	I149	V323	3202.5
V86	L174	L77	3223.2
V86	L174	I149	2617.3
V86	L174	V186	2549.8
V86	L174	V323	2715.4
V86	V186	L174	2905.9
V86	V323	I149	3300.8
V86	V323	L174	3032.7
I87	L30	L119	3703.2
I87	L77	L78	3772.2
I87	L77	M81	4250.0
I87	L77	L141	3378.3
I87	L77	I149	4221.9

First Res	Second Res	Third Res	Scaled Flow
I87	L77	L174	3980.1
I87	L78	L77	5136.1
I87	L78	M81	4339.8
I87	L78	L89	4246.2
I87	L78	L141	3665.7
I87	L78	I149	4977.7
I87	L78	V343	4095.9
I87	M81	L77	4540.6
I87	M81	L78	3405.3
I87	M81	L141	3256.5
I87	M81	I149	3394.6
I87	I87	L30	268.0
I87	I87	V41	39.5
I87	I87	L58	749.2
I87	I87	L75	289.0
I87	I87	L77	66.0
I87	I87	L78	225.6
I87	I87	M81	87.9
I87	I87	L89	633.3
I87	I87	M109	471.9
I87	I87	L119	74.0
I87	I87	L141	35.0
I87	I87	I149	21.7
I87	I87	L174	22.3
I87	I87	V323	927.3
I87	I87	V343	347.8
I87	L89	L78	5936.6
I87	L89	M109	4390.7
I87	L89	L141	5592.7
I87	M109	L89	3950.4
I87	M109	V343	3742.9
I87	L119	L30	2658.2
I87	L141	L77	2949.3
I87	L141	L78	2350.4
I87	L141	M81	2661.0
I87	L141	L89	2564.9
I87	I149	L77	3351.1
I87	I149	L78	2901.8
I87	I149	M81	2522.0
I87	I149	L174	2610.7
I87	I149	V323	2258.7
I87	L174	L77	3175.1
I87	L174	I149	2623.8
I87	L174	V323	1967.8
I87	V323	I149	6317.6
I87	V323	L174	5476.5
I87	V343	L78	4660.2
I87	V343	M109	3385.6
L89	L30	L58	2409.3
L89	L30	L75	2869.1
L89	L30	L119	2895.2
L89	V41	I169	3289.7
L89	L58	L30	4698.5
L89	L75	L30	3181.5
L89	L77	L78	3038.4
L89	L77	M81	4859.9
L89	L77	I149	4413.9
L89	L78	L77	10368.1
L89	L78	M81	8669.9
L89	L78	I149	9126.6
L89	L78	V343	6789.7
L89	M81	L77	4584.0
L89	M81	L78	2396.5
L89	I87	V323	4699.6
L89	L89	V26	323.1
L89	L89	L30	86.1
L89	L89	V41	164.6
L89	L89	L58	787.4
L89	L89	L75	131.6
L89	L89	L77	181.9
L89	L89	L78	2954.7
L89	L89	M81	145.8
L89	L89	I87	633.3
L89	L89	M109	1555.6
L89	L89	M112	118.6
L89	L89	L116	64.5
L89	L89	L119	149.2
L89	L89	M137	37.7
L89	L89	L141	927.3
L89	L89	I144	35.0
L89	L89	I149	131.6
L89	L89	I169	155.0

First Res	Second Res	Third Res	Scaled Flow
L89	L89	L170	1314.1
L89	L89	M182	55.0
L89	L89	V323	131.6
L89	L89	V343	601.7
L89	L119	L30	3318.3
L89	M137	L141	2220.0
L89	L141	M137	5561.3
L89	L141	I144	6588.9
L89	L141	I169	5797.0
L89	I144	L141	2591.0
L89	I149	L77	4055.5
L89	I149	L78	2457.4
L89	I149	V323	3411.2
L89	I169	V41	3237.8
L89	I169	L141	3209.8
L89	V323	I87	2909.9
L89	V323	I149	3411.2
L89	V343	L78	2896.8
L90	L77	L141	3026.7
L90	L77	L174	3177.5
L90	L90	L66	58.9
L90	L90	L77	35.0
L90	L90	L141	45.6
L90	L90	I144	123.6
L90	L90	L174	161.3
L90	L90	M182	51.3
L90	L90	V186	51.3
L90	L90	V187	33.4
L90	L90	V293	95.9
L90	L90	L357	111.6
L90	L90	V361	188.0
L90	L90	L367	368.4
L90	L141	L77	3198.3
L90	L141	I144	3234.0
L90	I144	L141	4067.7
L90	I144	L174	3149.4
L90	L174	L77	4522.0
L90	L174	I144	3372.1
L90	M182	V187	3604.3
L90	V186	V187	3423.8
L90	V187	M182	3293.1

First Res	Second Res	Third Res	Scaled Flow
L90	V187	V186	3128.2
M109	V26	M112	4180.9
M109	V41	I169	3313.1
M109	L77	L78	3335.0
M109	L77	M81	3649.2
M109	L77	L141	2873.6
M109	L78	L77	6914.4
M109	L78	M81	4095.8
M109	L78	L89	4329.9
M109	L78	V343	4183.7
M109	M81	L77	6656.5
M109	M81	L78	3603.5
M109	I87	V323	4668.0
M109	L89	L78	6081.2
M109	L89	L141	5493.1
M109	M109	V26	416.8
M109	M109	V41	87.9
M109	M109	L58	374.1
M109	M109	L75	37.7
M109	M109	L77	60.2
M109	M109	L78	749.2
M109	M109	M81	533.4
M109	M109	I87	471.9
M109	M109	L89	1555.6
M109	M109	M112	58.9
M109	M109	L116	56.2
M109	M109	L141	196.9
M109	M109	I169	61.6
M109	M109	L170	677.6
M109	M109	V323	87.9
M109	M109	V343	912.4
M109	M112	V26	2482.1
M109	M112	L170	2474.0
M109	L141	L77	3843.4
M109	L141	L89	2523.2
M109	L141	I169	4570.2
M109	I169	V41	3054.3
M109	I169	L141	3434.2
M109	L170	M112	4956.1
M109	V323	I87	2911.9
M109	V343	L78	4539.7

First Res	Second Res	Third Res	Scaled Flow
M112	V26	L78	4358.5
M112	V53	L116	3603.7
M112	V53	V161	5005.0
M112	V53	I215	3956.3
M112	L78	V26	2841.2
M112	L78	L89	4597.7
M112	L78	M109	3657.5
M112	L78	V343	4220.2
M112	L89	L78	4502.9
M112	L89	M109	4202.2
M112	L89	L141	3813.8
M112	L89	L170	3127.3
M112	L89	V343	3234.2
M112	M109	L78	3041.5
M112	M109	L89	3568.1
M112	M109	L170	2457.3
M112	M109	V343	3219.0
M112	M112	V16	181.9
M112	M112	V26	542.7
M112	M112	V53	161.3
M112	M112	L78	128.9
M112	M112	L89	118.6
M112	M112	M109	58.9
M112	M112	L116	121.1
M112	M112	L119	105.9
M112	M112	M137	72.1
M112	M112	L141	52.5
M112	M112	V161	185.6
M112	M112	L170	689.1
M112	M112	I215	51.3
M112	M112	V343	109.0
M112	L116	V53	3348.1
M112	M137	L141	3239.5
M112	L141	L89	3157.7
M112	L141	M137	3018.2
M112	V161	V53	5195.6
M112	V161	I215	4793.0
M112	L170	L89	5354.2
M112	L170	M109	4954.8
M112	I215	V53	3012.5
M112	I215	V161	3515.7

First Res	Second Res	Third Res	Scaled Flow
M112	V343	L78	4048.3
M112	V343	L89	3167.8
M112	V343	M109	3713.2
L116	V26	L78	3232.4
L116	V26	M109	2941.8
L116	V26	M112	2899.6
L116	V26	L170	2841.3
L116	V41	I169	2590.1
L116	V53	V161	6284.0
L116	L78	V26	3453.7
L116	L78	M81	4117.7
L116	L78	L89	4515.9
L116	L78	M109	3471.0
L116	L78	L141	3185.5
L116	L78	V343	4262.6
L116	M81	L78	3043.6
L116	M81	M109	2667.2
L116	M81	L141	2695.3
L116	M81	L170	2247.5
L116	L89	L78	4184.1
L116	L89	M109	3817.5
L116	L89	L141	3591.6
L116	L89	L170	3373.5
L116	L89	V343	3229.8
L116	M109	V26	2825.2
L116	M109	L78	3120.0
L116	M109	M81	3243.5
L116	M109	L89	3703.5
L116	M109	L170	2867.8
L116	M109	V343	3437.2
L116	M112	V26	3330.1
L116	M112	L170	3168.2
L116	L116	V16	60.2
L116	L116	V26	67.5
L116	L116	V41	22.3
L116	L116	V53	827.3
L116	L116	L78	89.9
L116	L116	M81	21.7
L116	L116	L89	64.5
L116	L116	M109	56.2
L116	L116	M112	121.1

First Res	Second Res	Third Res	Scaled Flow
L116	L116	L119	26.7
L116	L116	M137	234.5
L116	L116	L141	31.8
L116	L116	L154	48.9
L116	L116	L159	463.7
L116	L116	V161	506.2
L116	L116	I169	139.9
L116	L116	L170	178.3
L116	L116	I215	347.8
L116	L116	M219	33.5
L116	L116	V343	42.5
L116	M137	L141	4115.9
L116	M137	L159	3162.4
L116	L141	L78	2538.4
L116	L141	M81	2905.7
L116	L141	L89	3088.9
L116	L141	M137	2558.5
L116	L141	I169	3227.9
L116	L141	L170	2460.8
L116	L154	L159	2408.2
L116	L159	M137	3910.8
L116	L159	L154	4377.3
L116	L159	V161	3688.9
L116	V161	V53	5213.1
L116	V161	L159	3802.7
L116	V161	I215	4709.4
L116	I169	V41	3887.5
L116	I169	L141	4516.5
L116	L170	V26	3607.0
L116	L170	M81	3612.9
L116	L170	L89	4326.3
L116	L170	M109	3790.9
L116	L170	M112	3502.2
L116	L170	L141	3669.3
L116	I215	V161	4157.3
L116	V343	L78	3607.2
L116	V343	L89	2949.9
L116	V343	M109	3235.9
L119	V26	M112	2660.6
L119	V26	L170	2512.2
L119	V33	V53	3296.9

First Res	Second Res	Third Res	Scaled Flow
L119	V41	I169	2996.8
L119	V53	V33	3049.0
L119	V53	L116	3619.9
L119	V53	V161	4457.1
L119	V53	L170	2690.0
L119	L58	I87	3855.9
L119	L58	L89	3599.2
L119	I87	L58	2992.5
L119	I87	L89	2974.0
L119	L89	L58	3313.0
L119	L89	I87	3527.3
L119	L89	L141	3720.4
L119	L89	L170	3665.4
L119	M112	V26	3505.5
L119	M112	L170	2991.9
L119	L116	V53	2930.6
L119	L116	V161	2519.7
L119	L119	V16	40.6
L119	L119	V23	26.7
L119	L119	V26	30.3
L119	L119	L30	521.2
L119	L119	V33	100.7
L119	L119	V41	79.9
L119	L119	V45	273.9
L119	L119	V53	72.4
L119	L119	L58	203.4
L119	L119	I87	74.0
L119	L119	L89	149.2
L119	L119	M112	105.9
L119	L119	L116	26.7
L119	L119	M137	22.7
L119	L119	L141	102.6
L119	L119	V161	143.3
L119	L119	I169	152.3
L119	L119	L170	249.1
L119	L119	L283	65.4
L119	M137	L141	2639.3
L119	M137	I169	2299.0
L119	L141	L89	3387.1
L119	L141	M137	3655.4
L119	L141	I169	3778.3

First Res	Second Res	Third Res	Scaled Flow
L119	V161	V53	5257.5
L119	V161	L116	3671.3
L119	I169	V41	3510.0
L119	I169	M137	3515.9
L119	I169	L141	4172.0
L119	L170	V26	4151.2
L119	L170	V53	3689.9
L119	L170	L89	4217.7
L119	L170	M112	3752.3
M120	M120	V33	46.3
M120	M120	L167	54.7
M120	M120	M219	43.6
M120	M120	I220	22.7
M120	L167	M219	3131.5
M120	M219	L167	2985.7
L125	L125	I220	144.7
L125	L125	L294	21.8
I130	I130	L141	36.8
I130	I130	I144	60.2
I130	I130	L159	61.6
I130	I130	L167	230.0
I130	I130	I169	258.0
I130	I130	M219	448.9
I130	I130	L283	53.7
I130	I130	L292	23.5
I130	L141	I144	3304.9
I130	L141	I169	3139.2
I130	I144	L141	3672.8
I130	I169	L141	5037.7
V134	V134	V45	42.6
V134	V134	L159	23.9
M137	V41	I169	2411.4
M137	V53	L116	3337.8
M137	V53	V161	4981.2
M137	V53	L170	3804.1
M137	V53	I215	4045.7
M137	L89	L141	2592.5
M137	L89	L170	3564.2
M137	M112	L170	3492.9
M137	L116	V53	3678.7
M137	M137	V41	25.3

First Res	Second Res	Third Res	Scaled Flow
M137	M137	V53	164.6
M137	M137	L89	37.7
M137	M137	M112	72.1
M137	M137	L116	234.5
M137	M137	L119	22.7
M137	M137	L141	612.1
M137	M137	I144	34.2
M137	M137	L159	841.0
M137	M137	V161	204.7
M137	M137	L167	424.3
M137	M137	I169	367.3
M137	M137	L170	26.7
M137	M137	I209	59.6
M137	M137	V212	253.2
M137	M137	I215	41.5
M137	M137	M216	35.9
M137	M137	M219	25.1
M137	M137	L283	148.8
M137	L141	L89	5498.0
M137	L141	I144	5853.0
M137	L141	I169	4551.2
M137	L141	L170	4820.1
M137	I144	L141	2705.2
M137	L159	V161	4898.7
M137	V161	V53	5284.1
M137	V161	L159	3047.5
M137	V161	I215	4967.9
M137	L167	M219	4723.1
M137	I169	V41	4646.9
M137	I169	L141	3817.0
M137	L170	V53	2516.4
M137	L170	L89	3322.9
M137	L170	M112	2828.0
M137	L170	L141	2119.0
M137	I215	V53	2928.8
M137	I215	V161	3390.2
M137	M219	L167	2335.7
M137	M219	L283	2354.1
M137	L283	M219	3504.8
L141	L58	I87	4139.2
L141	L77	L78	5279.6

First Res	Second Res	Third Res	Scaled Flow
L141	L77	M81	5534.7
L141	L77	I149	5653.2
L141	L77	L174	6042.9
L141	L78	L77	4333.1
L141	L78	M81	3814.2
L141	L78	L89	4369.0
L141	L78	M109	3790.9
L141	L78	I149	4570.0
L141	L78	V343	5839.5
L141	M81	L77	4792.4
L141	M81	L78	4024.1
L141	I87	L58	2742.1
L141	I87	L89	2228.3
L141	I87	M109	2482.3
L141	I87	V323	2995.2
L141	I87	V343	2707.2
L141	L89	L78	6011.4
L141	L89	I87	5666.7
L141	L89	M109	5599.7
L141	L89	L170	4492.3
L141	L89	V343	5628.9
L141	M109	L78	3079.4
L141	M109	I87	3726.8
L141	M109	L89	3306.0
L141	M109	V343	4318.7
L141	M112	L170	2577.9
L141	L116	L159	2816.7
L141	M137	L159	5416.1
L141	L141	V41	171.4
L141	L141	L58	200.8
L141	L141	L77	689.1
L141	L141	L78	394.9
L141	L141	M81	463.7
L141	L141	I87	35.0
L141	L141	L89	927.3
L141	L141	L90	45.6
L141	L141	M109	196.9
L141	L141	M112	52.5
L141	L141	L116	31.8
L141	L141	L119	102.6
L141	L141	I130	36.8

First Res	Second Res	Third Res	Scaled Flow
L141	L141	M137	612.1
L141	L141	I144	1159.7
L141	L141	I149	164.6
L141	L141	L159	35.0
L141	L141	L167	70.5
L141	L141	I169	1532.2
L141	L141	L170	455.5
L141	L141	L174	51.3
L141	L141	V212	22.3
L141	L141	V323	118.6
L141	L141	V343	33.4
L141	I144	L174	5743.5
L141	I149	L77	3591.5
L141	I149	L78	3537.5
L141	I149	V323	3567.1
L141	L159	L116	2873.7
L141	L159	M137	2515.8
L141	L170	L89	3424.0
L141	L170	M112	4588.1
L141	L174	L77	2907.7
L141	L174	I144	2211.4
L141	L174	V323	2680.7
L141	V323	I87	3939.7
L141	V323	I149	3279.5
L141	V323	L174	3253.9
L141	V343	L78	3127.9
L141	V343	I87	2680.3
L141	V343	L89	2191.4
L141	V343	M109	2847.9
I144	L77	L78	5323.9
I144	L77	M81	5229.4
I144	L77	I149	4514.2
I144	L77	L174	3458.5
I144	L78	L77	3519.5
I144	L78	M81	3283.6
I144	L78	L89	4088.0
I144	L78	I149	3228.2
I144	M81	L77	4211.3
I144	M81	L78	4000.1
I144	L89	L78	3986.8
I144	L89	L141	2386.1

First Res	Second Res	Third Res	Scaled Flow
I144	M137	L141	2137.8
I144	L141	L89	6715.1
I144	L141	M137	6046.6
I144	L141	I169	5574.9
I144	I144	L77	221.3
I144	I144	L78	39.5
I144	I144	M81	95.9
I144	I144	L89	35.0
I144	I144	L90	123.6
I144	I144	I130	60.2
I144	I144	M137	34.2
I144	I144	L141	1159.7
I144	I144	I149	148.8
I144	I144	I169	273.1
I144	I144	L174	633.3
I144	I144	V187	31.0
I144	I144	M201	25.3
I144	I144	V323	47.8
I144	I144	V333	27.4
I144	I149	L77	4055.0
I144	I149	L78	4386.5
I144	I149	V323	3844.0
I144	I169	L141	3265.1
I144	L174	L77	4861.7
I144	L174	M201	4742.3
I144	M201	L174	2037.8
I144	V323	I149	2944.8
I149	V26	L78	2172.1
I149	L58	L75	2580.2
I149	L58	L78	1828.1
I149	L58	I87	3095.5
I149	L58	L89	2778.4
I149	L58	V323	1982.9
I149	L75	L58	3045.0
I149	L77	L78	4905.2
I149	L77	M81	7439.9
I149	L77	L174	5950.5
I149	L78	V26	7224.1
I149	L78	L58	5870.2
I149	L78	L77	4038.8
I149	L78	L89	8029.0
I149	L78	V343	7778.5
I149	M81	L77	3954.4
I149	I87	L58	2973.8
I149	I87	L89	2594.8
I149	I87	V323	2402.3
I149	I87	V343	2411.9
I149	L89	L58	3963.5
I149	L89	L78	3566.9
I149	L89	I87	3853.1
I149	L89	L141	3472.0
I149	L89	V343	3436.0
I149	L141	L89	3679.4
I149	L141	I144	3825.3
I149	I144	L141	3725.4
I149	I149	V26	22.3
I149	I149	L58	26.7
I149	I149	L75	58.9
I149	I149	L77	1918.7
I149	I149	L78	1355.4
I149	I149	M81	471.9
I149	I149	V86	50.2
I149	I149	I87	21.7
I149	I149	L89	131.6
I149	I149	L141	164.6
I149	I149	I144	148.8
I149	I149	L174	347.8
I149	I149	M182	69.0
I149	I149	V323	774.5
I149	I149	V343	70.5
I149	L174	L77	2860.8
I149	V323	L58	4941.5
I149	V323	I87	6231.6
I149	V343	L78	2976.7
I149	V343	I87	3085.2
I149	V343	L89	2959.9
I150	I150	L198	53.7
I150	I150	M201	230.0
I150	I150	I220	44.5
I150	I150	M239	58.9
I150	I150	V241	56.2
I150	I150	L253	31.8

First Res	Second Res	Third Res	Scaled Flow
I150	I150	V293	78.8
I150	I150	L337	106.7
I150	I150	V361	31.1
I150	L198	M239	3496.3
I150	L198	L253	3448.3
I150	M201	L253	5222.9
I150	M239	L198	3567.5
I150	M239	V241	3380.9
I150	M239	L253	2949.9
I150	V241	M239	3346.9
I150	L253	L198	3088.0
I150	L253	M201	3264.6
I150	L253	M239	2588.9
I150	V293	V361	3194.2
I150	V361	V293	2615.0
L154	V53	L116	3972.7
L154	V53	V161	5134.4
L154	V53	L170	3733.1
L154	V53	I215	4068.6
L154	L116	V53	2963.0
L154	L116	V161	2690.5
L154	L116	I215	2788.8
L154	L154	V53	168.0
L154	L154	L116	48.9
L154	L154	L159	542.7
L154	L154	V161	148.8
L154	L154	L167	28.0
L154	L154	L170	34.2
L154	L154	I215	40.5
L154	L154	M219	97.2
L154	L159	V161	4684.4
L154	V161	V53	4973.7
L154	V161	L116	3494.5
L154	V161	L159	3167.9
L154	V161	I215	4678.9
L154	L167	M219	2696.7
L154	L170	V53	2582.5
L154	I215	V53	2915.1
L154	I215	L116	2679.0
L154	I215	V161	3460.6
L154	M219	L167	3538.0

First Res	Second Res	Third Res	Scaled Flow
L159	V53	V161	4922.9
L159	V53	L170	4219.3
L159	V53	I215	4324.2
L159	M137	L141	5459.2
L159	L141	M137	2237.7
L159	L141	I169	3047.8
L159	L141	L170	2714.1
L159	L159	V53	480.3
L159	L159	L116	463.7
L159	L159	I130	61.6
L159	L159	V134	23.9
L159	L159	M137	841.0
L159	L159	L141	35.0
L159	L159	L154	542.7
L159	L159	V161	1106.0
L159	L159	L167	323.1
L159	L159	I169	341.4
L159	L159	L170	66.0
L159	L159	I215	134.3
L159	L159	M219	244.4
L159	L159	L283	109.0
L159	V161	V53	6864.8
L159	V161	I215	6618.8
L159	I169	L141	5377.2
L159	L170	V53	2448.7
L159	L170	L141	3108.8
L159	I215	V53	2973.4
L159	I215	V161	3263.7
V161	V33	V53	1680.7
V161	V41	I169	2929.0
V161	V53	V33	11765.3
V161	M112	L170	3153.8
V161	M137	L167	3681.7
V161	V161	V33	46.8
V161	V161	V41	89.9
V161	V161	V53	4391.7
V161	V161	M112	185.6
V161	V161	L116	506.2
V161	V161	L119	143.3
V161	V161	M137	204.7
V161	V161	L154	148.8

First Res	Second Res	Third Res	Scaled Flow
V161	V161	L159	1106.0
V161	V161	L167	33.4
V161	V161	I169	212.9
V161	V161	L170	278.3
V161	V161	I215	1890.5
V161	V161	M219	62.5
V161	V161	L283	26.0
V161	L167	M137	2401.7
V161	L167	M219	2850.0
V161	L167	L283	2785.9
V161	I169	V41	3653.4
V161	L170	M112	3538.5
V161	M219	L167	3260.5
V161	M219	L283	3052.9
V161	L283	L167	2649.8
V161	L283	M219	2538.2
L167	M137	L141	4225.9
L167	L141	M137	2596.9
L167	L141	I169	4062.9
L167	L154	L159	2382.6
L167	L159	L154	4322.4
L167	L159	V161	4975.9
L167	V161	L159	2840.6
L167	V161	I215	3670.2
L167	L167	M120	54.7
L167	L167	I130	230.0
L167	L167	M137	424.3
L167	L167	L141	70.5
L167	L167	L154	28.0
L167	L167	L159	323.1
L167	L167	V161	33.4
L167	L167	I169	25.3
L167	L167	I215	36.8
L167	L167	M219	588.2
L167	L167	L283	380.9
L167	I169	L141	3273.3
L167	I215	V161	3744.5
I169	V33	V53	2704.1
I169	V53	V33	3745.6
I169	V53	L116	3524.2
I169	V53	V161	4911.6
I169	V53	I215	3748.5
I169	L58	L89	3554.4
I169	L77	M81	3938.3
I169	L77	L141	2131.2
I169	M81	L77	4557.9
I169	M81	M109	3114.3
I169	L89	L58	3351.6
I169	L89	M109	4387.7
I169	L89	L141	2505.9
I169	L89	L170	3645.7
I169	M109	M81	2990.6
I169	M109	L89	3515.5
I169	M109	L170	2770.8
I169	L116	V53	3432.5
I169	M137	L159	3531.0
I169	M137	L167	4239.9
I169	L141	L77	6805.4
I169	L141	L89	5768.8
I169	L141	I144	5484.8
I169	I144	L141	2788.9
I169	L159	M137	3450.2
I169	L159	V161	4073.0
I169	V161	V53	5351.7
I169	V161	L159	3542.0
I169	V161	I215	4718.9
I169	L167	M137	2200.2
I169	L167	L283	2271.3
I169	I169	V33	37.8
I169	I169	V41	633.3
I169	I169	V53	155.0
I169	I169	L58	193.0
I169	I169	L77	37.7
I169	I169	M81	73.8
I169	I169	L89	155.0
I169	I169	M109	61.6
I169	I169	L116	139.9
I169	I169	L119	152.3
I169	I169	I130	258.0
I169	I169	M137	367.3
I169	I169	L141	1532.2
I169	I169	I144	273.1

First Res	Second Res	Third Res	Scaled Flow
I169	I169	L159	341.4
I169	I169	V161	212.9
I169	I169	L167	25.3
I169	I169	L170	273.1
I169	I169	V212	95.9
I169	I169	I215	84.2
I169	I169	I220	24.1
I169	I169	L283	212.9
I169	L170	L89	4267.5
I169	L170	M109	4048.0
I169	I215	V53	3224.7
I169	I215	V161	3725.6
I169	L283	L167	3719.2
L170	V41	L58	2639.5
L170	V41	I169	2586.6
L170	V53	L116	4180.8
L170	V53	V161	6128.7
L170	V53	I215	5334.0
L170	L58	V41	2831.7
L170	L58	L75	3000.2
L170	L58	L89	2308.9
L170	L75	L58	2594.1
L170	L77	L78	3431.5
L170	L77	M81	3706.7
L170	L77	L141	2577.9
L170	L78	L77	6181.3
L170	L78	M81	4101.3
L170	L78	L89	4205.7
L170	L78	V343	6327.0
L170	M81	L77	6109.8
L170	M81	L78	3752.8
L170	L89	L58	6342.7
L170	L89	L78	6413.7
L170	L89	M109	4645.7
L170	L89	V343	6692.6
L170	M109	L89	3474.3
L170	M109	V343	5971.2
L170	L116	V53	2984.3
L170	L116	L159	3415.1
L170	M137	L141	2351.8
L170	M137	L159	2957.0

First Res	Second Res	Third Res	Scaled Flow
L170	L141	L77	4588.1
L170	L141	M137	4816.6
L170	L141	I169	4517.8
L170	L154	L159	2803.2
L170	L159	L116	2676.5
L170	L159	M137	3578.9
L170	L159	L154	3227.1
L170	L159	V161	3122.5
L170	V161	V53	4961.2
L170	V161	L159	4518.4
L170	V161	I215	5429.6
L170	I169	V41	4199.7
L170	I169	L141	3841.9
L170	L170	V26	591.5
L170	L170	V33	109.3
L170	L170	V41	37.7
L170	L170	V53	533.4
L170	L170	L58	52.5
L170	L170	L75	26.0
L170	L170	L77	52.5
L170	L170	L78	471.9
L170	L170	M81	360.7
L170	L170	L89	1314.1
L170	L170	M109	677.6
L170	L170	M112	689.1
L170	L170	L116	178.3
L170	L170	L119	249.1
L170	L170	M137	26.7
L170	L170	L141	455.5
L170	L170	L154	34.2
L170	L170	L159	66.0
L170	L170	V161	278.3
L170	L170	I169	273.1
L170	L170	I215	30.2
L170	L170	V343	21.7
L170	I215	V53	2527.9
L170	I215	V161	3178.8
L170	V343	L78	2932.3
L170	V343	L89	2033.9
L170	V343	M109	2426.5
L174	L58	L78	2836.8

First Res	Second Res	Third Res	Scaled Flow
L174	L58	I87	3529.6
L174	L77	L78	8408.4
L174	L77	M81	7320.3
L174	L77	L141	6264.7
L174	L77	I149	6031.1
L174	L78	L58	2880.2
L174	L78	L77	3151.1
L174	L78	M81	3224.4
L174	L78	L141	2941.1
L174	L78	I149	3214.2
L174	M81	L77	3951.8
L174	M81	L78	4644.9
L174	M81	L141	3800.7
L174	I87	L58	2830.0
L174	I87	V323	2545.6
L174	L141	L77	2198.6
L174	L141	L78	2754.3
L174	L141	M81	2470.8
L174	L141	I144	2807.7
L174	I144	L141	5649.7
L174	I149	L77	3466.1
L174	I149	L78	4929.1
L174	L174	L58	64.5
L174	L174	L77	1397.8
L174	L174	L78	69.0
L174	L174	M81	283.6
L174	L174	V86	34.3
L174	L174	I87	22.3
L174	L174	L90	161.3
L174	L174	L141	51.3
L174	L174	I144	633.3
L174	L174	I149	347.8
L174	L174	M182	66.0
L174	L174	V186	335.2
L174	L174	V187	123.6
L174	L174	M201	402.1
L174	L174	V323	439.7
L174	L174	V333	42.6
L174	L174	L357	22.7
L174	M182	V187	3401.9
L174	V186	V187	4123.3

First Res	Second Res	Third Res	Scaled Flow
L174	V187	M182	3947.3
L174	V187	V186	3124.1
L174	V323	I87	5335.3
M182	L77	L78	4496.0
M182	L77	M81	5165.4
M182	L77	I149	4675.8
M182	L77	L174	4390.8
M182	L78	L77	4520.2
M182	L78	M81	4287.7
M182	L78	L89	5215.5
M182	L78	I149	4360.1
M182	M81	L77	4067.6
M182	M81	L78	3358.4
M182	M81	I149	2975.4
M182	L89	L78	3883.8
M182	I149	L77	3682.0
M182	I149	L78	3415.1
M182	I149	M81	2975.4
M182	L174	L77	3422.7
M182	M182	L66	300.0
M182	M182	L77	181.9
M182	M182	L78	185.6
M182	M182	M81	69.0
M182	M182	L89	55.0
M182	M182	L90	51.3
M182	M182	I149	69.0
M182	M182	L174	66.0
M182	M182	V186	145.8
M182	M182	V187	1159.7
M182	M182	V333	27.1
M182	V186	V187	2602.1
M182	V187	V186	5283.7
V186	L77	L174	3221.9
V186	L90	L367	2750.8
V186	L174	L77	4959.3
V186	M182	V187	2969.2
V186	V186	L66	28.0
V186	V186	L77	64.5
V186	V186	V86	63.2
V186	V186	L90	51.3
V186	V186	L174	335.2

First Res	Second Res	Third Res	Scaled Flow
V186	V186	M182	145.8
V186	V186	V187	883.2
V186	V186	V300	32.5
V186	V186	V333	38.7
V186	V186	L357	261.3
V186	V186	L367	56.6
V186	V187	M182	5335.3
V186	L367	L90	2832.5
V187	L77	L174	3700.4
V187	I144	L174	2719.4
V187	L174	L77	3998.3
V187	L174	I144	3706.0
V187	V187	L66	80.6
V187	V187	L77	89.9
V187	V187	L90	33.4
V187	V187	I144	31.0
V187	V187	L174	123.6
V187	V187	M182	1159.7
V187	V187	V186	883.2
V187	V187	V300	40.5
V187	V187	V333	62.5
I197	I197	L198	1627.9
I197	I197	L225	100.1
I197	I197	I238	263.8
I197	I197	M239	82.4
I197	I197	L253	126.2
I197	I197	L265	42.2
I197	I197	L268	225.6
I197	I197	I276	72.1
I197	I197	L288	80.6
I197	I197	L292	121.1
I197	I197	V293	200.8
I197	I197	L294	278.3
I197	L198	M239	7132.3
I197	L198	L253	6017.3
I197	L225	I238	2946.1
I197	L225	I276	3539.0
I197	L225	L294	3859.1
I197	I238	L225	3811.2
I197	I238	I276	5747.8
I197	I238	L292	4231.9

First Res	Second Res	Third Res	Scaled Flow
I197	M239	L198	2564.4
I197	M239	L294	2890.8
I197	L253	L198	2398.5
I197	L253	L268	3225.6
I197	L268	L253	3765.3
I197	I276	L225	3277.8
I197	I276	I238	4115.2
I197	I276	L294	3145.7
I197	L292	I238	3427.2
I197	L294	L225	5068.4
I197	L294	M239	3975.9
I197	L294	I276	4460.6
L198	L198	I150	53.7
L198	L198	I197	1627.9
L198	L198	M201	174.8
L198	L198	L225	123.6
L198	L198	I238	279.2
L198	L198	M239	1159.7
L198	L198	L253	869.0
L198	L198	M262	91.8
L198	L198	L265	67.9
L198	L198	L268	185.6
L198	L198	I276	134.3
L198	L198	L292	118.6
L198	L198	L294	581.5
L198	M201	L253	3512.7
L198	M201	L268	3183.0
L198	L225	I238	2997.4
L198	L225	I276	3438.5
L198	L225	L294	3672.5
L198	I238	L225	3744.2
L198	I238	I276	5517.0
L198	I238	L292	4285.7
L198	L253	M201	5976.4
L198	M262	L294	2541.5
L198	L268	M201	3234.6
L198	I276	L225	3511.1
L198	I276	I238	4509.8
L198	I276	L294	3087.6
L198	L292	I238	3395.4
L198	L294	L225	5836.9

First Res	Second Res	Third Res	Scaled Flow
L198	L294	M262	4342.2
L198	L294	I276	4805.7
M201	I144	L174	2389.7
M201	L174	I144	4741.8
M201	L198	M239	3878.9
M201	L198	L253	2325.0
M201	M201	I144	25.3
M201	M201	I150	230.0
M201	M201	L174	402.1
M201	M201	L198	174.8
M201	M201	M239	142.8
M201	M201	L253	1947.2
M201	M201	M262	80.6
M201	M201	L265	42.7
M201	M201	L268	591.5
M201	M239	L198	3678.7
M201	L253	L198	5957.0
I209	I209	M137	59.6
I209	I209	M216	199.3
I209	I209	L288	43.0
I209	I209	V293	55.6
I209	L288	V293	2701.8
I209	V293	L288	2855.7
V212	M137	L141	4316.7
V212	L141	M137	2447.0
V212	L141	I169	3157.9
V212	I169	L141	4316.8
V212	V212	M137	253.2
V212	V212	L141	22.3
V212	V212	I169	95.9
V212	V212	L283	21.7
V212	V212	M291	56.2
I215	V53	V161	4885.5
I215	V53	L170	5398.4
I215	M112	L170	3286.4
I215	M137	L159	2969.7
I215	M137	L167	2860.3
I215	M137	I169	2595.0
I215	L154	L159	2698.2
I215	L159	M137	3891.4
I215	L159	L154	3553.3

First Res	Second Res	Third Res	Scaled Flow
I215	L159	V161	2526.5
I215	V161	V53	7274.7
I215	V161	L159	6809.2
I215	L167	M137	2789.5
I215	L167	M219	2776.1
I215	I169	M137	3035.0
I215	L170	V53	2132.7
I215	L170	M112	2943.1
I215	I215	V53	854.9
I215	I215	M112	51.3
I215	I215	L116	347.8
I215	I215	M137	41.5
I215	I215	L154	40.5
I215	I215	L159	134.3
I215	I215	V161	1890.5
I215	I215	L167	36.8
I215	I215	I169	84.2
I215	I215	L170	30.2
I215	I215	M219	102.6
I215	M219	L167	3491.7
M216	M216	M137	35.9
M216	M216	I209	199.3
M216	M216	M291	21.2
M219	L116	L159	2407.4
M219	L116	V161	2767.9
M219	L116	I215	2456.8
M219	M137	L159	2659.7
M219	M137	L167	2082.6
M219	L154	L159	2796.7
M219	L159	L116	3877.6
M219	L159	M137	4538.1
M219	L159	L154	3560.9
M219	L159	V161	4430.1
M219	V161	L116	3162.4
M219	V161	L159	3142.3
M219	V161	I215	3783.7
M219	L167	M137	4724.4
M219	I215	L116	3148.3
M219	I215	V161	4243.9
M219	M219	L116	33.5
M219	M219	M120	43.6

First Res	Second Res	Third Res	Scaled Flow
M219	M219	I130	448.9
M219	M219	M137	25.1
M219	M219	L154	97.2
M219	M219	L159	244.4
M219	M219	V161	62.5
M219	M219	L167	588.2
M219	M219	I215	102.6
M219	M219	L283	385.5
I220	I220	V33	32.2
I220	I220	M120	22.7
I220	I220	L125	144.7
I220	I220	I150	44.5
I220	I220	I169	24.1
I220	I220	L277	28.0
I220	I220	L287	69.0
I220	I220	L288	80.6
I220	I220	V293	208.8
I220	I220	V323	21.2
I220	L277	L288	2711.5
I220	L288	L277	3401.0
L225	I197	L198	3884.6
L225	L198	I197	4091.0
L225	L198	M239	3270.3
L225	L225	I197	100.1
L225	L225	L198	123.6
L225	L225	I238	685.4
L225	L225	M239	402.1
L225	L225	V241	601.7
L225	L225	L265	48.1
L225	L225	I276	841.0
L225	L225	L277	927.3
L225	L225	L288	139.9
L225	L225	L292	95.9
L225	L225	L294	2221.5
L225	I238	I276	5247.7
L225	I238	L292	5323.6
L225	M239	L198	4574.6
L225	I276	I238	5692.5
L225	L292	I238	2956.8
I238	I197	L198	4159.1
I238	L198	I197	4229.4

First Res	Second Res	Third Res	Scaled Flow
I238	L198	M239	4168.0
I238	L198	L253	3859.8
I238	L225	L294	4862.7
I238	I238	I197	263.8
I238	I238	L198	279.2
I238	I238	L225	685.4
I238	I238	M239	150.8
I238	I238	V241	219.8
I238	I238	L253	155.4
I238	I238	L265	259.1
I238	I238	L268	51.4
I238	I238	I276	3696.9
I238	I238	L277	318.1
I238	I238	L288	120.2
I238	I238	L292	1154.0
I238	I238	V293	55.8
I238	I238	L294	539.6
I238	M239	L198	3512.5
I238	M239	V241	3394.7
I238	M239	L294	2805.2
I238	V241	M239	3759.1
I238	L253	L198	3278.6
I238	L253	L268	3888.4
I238	L268	L253	2991.9
I238	L294	L225	4449.0
I238	L294	M239	4125.6
M239	I197	L198	2966.9
M239	L198	I197	6928.5
M239	M201	L253	3828.2
M239	M201	L268	3227.9
M239	L225	I238	3866.5
M239	L225	I276	3624.8
M239	L225	L294	4211.9
M239	I238	L225	2909.7
M239	I238	I276	4544.3
M239	M239	I150	58.9
M239	M239	I197	82.4
M239	M239	L198	1159.7
M239	M239	M201	142.8
M239	M239	L225	402.1
M239	M239	I238	150.8

First Res	Second Res	Third Res	Scaled Flow
M239	M239	V241	897.7
M239	M239	V250	24.1
M239	M239	L253	367.3
M239	M239	M262	63.0
M239	M239	L265	71.0
M239	M239	L268	134.3
M239	M239	I276	317.2
M239	M239	L294	712.6
M239	M239	V300	23.5
M239	V250	M262	2817.2
M239	L253	M201	5013.5
M239	L253	L268	4057.6
M239	M262	V250	3443.7
M239	M262	L294	2392.5
M239	L268	M201	3177.5
M239	L268	L253	3049.9
M239	I276	L225	3362.0
M239	I276	I238	5600.6
M239	I276	L294	3270.1
M239	L294	L225	5169.0
M239	L294	M262	4814.1
M239	L294	I276	4326.9
V241	L225	L277	5783.6
V241	L225	L294	5517.9
V241	I238	I276	4929.5
V241	V241	I150	56.2
V241	V241	L225	601.7
V241	V241	I238	219.8
V241	V241	M239	897.7
V241	V241	V250	217.1
V241	V241	L253	60.2
V241	V241	M262	73.8
V241	V241	L265	59.2
V241	V241	I276	268.0
V241	V241	L277	22.3
V241	V241	L294	204.7
V241	V250	M262	3824.9
V241	M262	V250	2913.0
V241	M262	L294	2851.1
V241	I276	I238	5215.1
V241	I276	L294	3917.4

First Res	Second Res	Third Res	Scaled Flow
V241	L277	L225	2470.5
V241	L294	L225	3914.7
V241	L294	M262	3683.0
V241	L294	I276	3627.3
V250	M239	V241	2710.2
V250	M239	L253	2541.2
V250	M239	L294	2814.0
V250	V241	M239	4507.1
V250	V250	M239	24.1
V250	V250	V241	217.1
V250	V250	L253	47.8
V250	V250	M262	689.1
V250	V250	L265	132.4
V250	V250	L268	181.9
V250	V250	L294	70.5
V250	L253	M239	2924.1
V250	L253	L268	2922.5
V250	M262	L294	4696.0
V250	L268	L253	4023.3
V250	L294	M239	3528.0
V250	L294	M262	2425.1
L253	I197	L198	3225.6
L253	L198	I197	5971.3
L253	L198	M239	4455.5
L253	I238	I276	5420.9
L253	I238	L292	3796.5
L253	M239	L198	3252.6
L253	M239	V241	4597.0
L253	M239	L294	3728.7
L253	V241	M239	2858.0
L253	V250	M262	2710.6
L253	L253	I150	31.8
L253	L253	I197	126.2
L253	L253	L198	869.0
L253	L253	M201	1947.2
L253	L253	I238	155.4
L253	L253	M239	367.3
L253	L253	V241	60.2
L253	L253	V250	47.8
L253	L253	M262	268.0
L253	L253	L265	43.8

First Res	Second Res	Third Res	Scaled Flow
L253	L253	L268	813.8
L253	L253	I276	35.0
L253	L253	L292	145.8
L253	L253	L294	185.6
L253	M262	V250	4162.5
L253	I276	I238	3843.9
L253	I276	L294	2986.9
L253	L292	I238	3731.1
L253	L294	M239	3050.8
L253	L294	I276	4412.5
M262	L198	M239	3776.0
M262	L198	L253	3085.9
M262	M201	L253	3648.6
M262	M201	L268	2928.0
M262	M239	L198	3463.1
M262	M239	V241	3344.2
M262	M239	L294	2545.6
M262	V241	M239	3465.0
M262	L253	L198	4091.0
M262	L253	M201	4987.8
M262	L253	L268	3740.6
M262	M262	L198	91.8
M262	M262	M201	80.6
M262	M262	M239	63.0
M262	M262	V241	73.8
M262	M262	V250	689.1
M262	M262	L253	268.0
M262	M262	L265	252.3
M262	M262	L268	164.6
M262	M262	L294	612.1
M262	L268	M201	3493.8
M262	L268	L253	3265.0
M262	L294	M239	4833.0
L265	I197	L198	3567.7
L265	L198	I197	3956.2
L265	L198	M239	3571.3
L265	L198	L253	3495.1
L265	L198	L294	2875.6
L265	M201	L253	3794.8
L265	M201	L268	2542.7
L265	L225	I238	2717.6

First Res	Second Res	Third Res	Scaled Flow
L265	L225	M239	2735.6
L265	L225	V241	3019.6
L265	L225	I276	3247.9
L265	L225	L294	3649.7
L265	I238	L225	4129.9
L265	I238	I276	5849.4
L265	M239	L198	3607.7
L265	M239	L225	2980.9
L265	M239	V241	3462.0
L265	M239	L253	2948.5
L265	M239	L294	3028.3
L265	V241	L225	3159.7
L265	V241	M239	3324.6
L265	V250	M262	3068.1
L265	L253	L198	3175.8
L265	L253	M201	3813.9
L265	L253	M239	2652.0
L265	L253	L268	2743.9
L265	M262	V250	3654.0
L265	M262	L294	3468.7
L265	L265	I197	42.2
L265	L265	L198	67.9
L265	L265	M201	42.7
L265	L265	L225	48.1
L265	L265	I238	259.1
L265	L265	M239	71.0
L265	L265	V241	59.2
L265	L265	V250	132.4
L265	L265	L253	43.8
L265	L265	M262	252.3
L265	L265	L268	313.2
L265	L265	I276	54.7
L265	L265	L294	154.3
L265	L268	M201	4190.7
L265	L268	L253	4499.6
L265	I276	L225	3339.1
L265	I276	I238	3957.1
L265	I276	L294	3210.8
L265	L294	L198	3508.6
L265	L294	L225	4803.3
L265	L294	M239	3657.7

First Res	Second Res	Third Res	Scaled Flow
L265	L294	M262	3029.8
L265	L294	I276	4110.3
L268	I197	L198	4257.1
L268	L198	I197	4032.4
L268	L198	M239	3942.6
L268	L198	L253	2819.9
L268	M201	L253	4181.8
L268	I238	I276	4383.6
L268	I238	L294	2641.7
L268	M239	L198	3622.0
L268	M239	L294	3160.8
L268	V250	M262	3381.8
L268	L253	L198	4593.8
L268	L253	M201	4734.4
L268	M262	V250	3292.7
L268	M262	L294	3119.0
L268	L268	I197	225.6
L268	L268	L198	185.6
L268	L268	M201	591.5
L268	L268	I238	51.4
L268	L268	M239	134.3
L268	L268	V250	181.9
L268	L268	L253	813.8
L268	L268	M262	164.6
L268	L268	L265	313.2
L268	L268	I276	44.5
L268	L268	L294	217.1
L268	I276	I238	4247.5
L268	I276	L294	3035.9
L268	L294	I238	3755.5
L268	L294	M239	3592.8
L268	L294	M262	3363.1
L268	L294	I276	4454.3
I276	I197	L198	3672.4
I276	L198	I197	4263.9
I276	L198	M239	3404.6
I276	L198	L253	3972.4
I276	L225	L277	4758.5
I276	L225	L294	4331.6
I276	I238	L292	8784.5
I276	M239	L198	4325.8

First Res	Second Res	Third Res	Scaled Flow
I276	M239	V241	3671.1
I276	V241	M239	3489.6
I276	L253	L198	2927.0
I276	L253	L268	3129.5
I276	L268	L253	3290.4
I276	I276	I197	72.1
I276	I276	L198	134.3
I276	I276	L225	841.0
I276	I276	I238	3696.9
I276	I276	M239	317.2
I276	I276	V241	268.0
I276	I276	L253	35.0
I276	I276	L265	54.7
I276	I276	L268	44.5
I276	I276	L277	178.3
I276	I276	L288	168.0
I276	I276	L292	208.8
I276	I276	V293	64.5
I276	I276	L294	1088.6
I276	L277	L225	2850.7
I276	L292	I238	2177.5
I276	L294	L225	4850.9
L277	L225	V241	5925.2
L277	L225	I276	4720.2
L277	L225	L294	7605.2
L277	I238	I276	5516.6
L277	I238	L292	4181.7
L277	I238	L294	4172.7
L277	V241	L225	2129.0
L277	I276	L225	2712.8
L277	I276	I238	4667.9
L277	I276	L294	4353.6
L277	L277	I220	28.0
L277	L277	L225	927.3
L277	L277	I238	318.1
L277	L277	V241	22.3
L277	L277	I276	178.3
L277	L277	L287	109.0
L277	L277	L288	561.8
L277	L277	M291	171.4
L277	L277	L292	174.8

First Res	Second Res	Third Res	Scaled Flow
L277	L277	L294	37.7
L277	L292	I238	3519.5
L277	L294	L225	3035.9
L277	L294	I238	2452.4
L277	L294	I276	3023.9
L283	M137	L159	3613.6
L283	L159	M137	3341.4
L283	L159	V161	4108.8
L283	V161	L159	3003.2
L283	L283	L119	65.4
L283	L283	I130	53.7
L283	L283	M137	148.8
L283	L283	L159	109.0
L283	L283	V161	26.0
L283	L283	L167	380.9
L283	L283	I169	212.9
L283	L283	V212	21.7
L283	L283	M219	385.5
L283	L283	V293	82.4
L287	L287	I220	69.0
L287	L287	L277	109.0
L288	L225	I238	3376.4
L288	L225	I276	3414.4
L288	L225	L277	2982.6
L288	I238	L225	3251.5
L288	I238	I276	4627.9
L288	I238	L292	3777.5
L288	I276	L225	3580.8
L288	I276	I238	5039.9
L288	L277	L225	4536.8
L288	L288	I197	80.6
L288	L288	I209	43.0
L288	L288	I220	80.6
L288	L288	L225	139.9
L288	L288	I238	120.2
L288	L288	I276	168.0
L288	L288	L277	561.8
L288	L288	L292	102.3
L288	L288	V293	367.3
L288	L288	V361	27.1
L288	L292	I238	3628.2

First Res	Second Res	Third Res	Scaled Flow
L288	V293	V361	4237.2
L288	V361	V293	2229.5
M291	M291	V212	56.2
M291	M291	M216	21.2
M291	M291	L277	171.4
L292	I197	L198	4017.3
L292	L198	I197	3996.5
L292	L198	L253	3414.2
L292	L198	L294	3549.1
L292	L225	I238	2345.9
L292	L225	I276	3166.7
L292	L225	L277	3301.8
L292	L225	L294	4472.2
L292	I238	L225	5277.1
L292	I238	I276	7549.3
L292	I238	L294	5732.3
L292	L253	L198	3597.5
L292	I276	L225	3868.5
L292	I276	I238	4099.7
L292	I276	L294	4476.7
L292	L277	L225	3842.7
L292	L277	L288	3389.4
L292	L288	L277	2957.6
L292	L292	I130	23.5
L292	L292	I197	121.1
L292	L292	L198	118.6
L292	L292	L225	95.9
L292	L292	I238	1154.0
L292	L292	L253	145.8
L292	L292	I276	208.8
L292	L292	L277	174.8
L292	L292	L288	102.3
L292	L292	V293	239.1
L292	L292	L294	38.6
L292	L294	L198	2752.9
L292	L294	L225	3652.8
L292	L294	I238	2081.3
L292	L294	I276	2993.1
V293	I238	I276	4351.8
V293	I238	L292	3134.9
V293	I276	I238	4490.7

First Res	Second Res	Third Res	Scaled Flow
V293	L292	I238	4500.4
V293	V293	L90	95.9
V293	V293	I150	78.8
V293	V293	I197	200.8
V293	V293	I209	55.6
V293	V293	I220	208.8
V293	V293	I238	55.8
V293	V293	I276	64.5
V293	V293	L283	82.4
V293	V293	L288	367.3
V293	V293	L292	239.1
V293	V293	V361	437.1
V293	V293	L367	60.7
L294	I197	L198	3772.0
L294	L198	I197	4807.2
L294	L198	L253	4283.7
L294	L225	L277	8734.3
L294	I238	I276	4784.6
L294	I238	L292	5588.6
L294	M239	V241	4428.2
L294	V241	M239	2945.6
L294	V250	M262	2547.9
L294	L253	L198	2995.8
L294	L253	L268	3412.9
L294	M262	V250	4716.4
L294	L268	L253	3563.9
L294	I276	I238	6352.8
L294	L277	L225	2186.4
L294	L292	I238	2771.0
L294	L294	L125	21.8
L294	L294	I197	278.3
L294	L294	L198	581.5
L294	L294	L225	2221.5
L294	L294	I238	539.6
L294	L294	M239	712.6
L294	L294	V241	204.7
L294	L294	V250	70.5
L294	L294	L253	185.6
L294	L294	M262	612.1
L294	L294	L265	154.3
L294	L294	L268	217.1

First Res	Second Res	Third Res	Scaled Flow
L294	L294	I276	1088.6
L294	L294	L277	37.7
L294	L294	L292	38.6
V300	V186	V187	3164.7
V300	V187	V186	3310.7
V300	V300	V186	32.5
V300	V300	V187	40.5
V300	V300	M239	23.5
V323	L30	L58	2649.9
V323	L30	L75	2851.1
V323	L58	L30	4105.7
V323	L58	L89	4087.2
V323	L75	L30	3193.2
V323	L77	L78	4951.7
V323	L77	M81	5193.7
V323	L77	L141	3686.0
V323	L77	I149	3709.6
V323	L77	L174	3705.6
V323	L78	L77	4085.4
V323	L78	M81	3755.1
V323	L78	L89	4495.5
V323	L78	M109	3475.9
V323	L78	I149	3101.2
V323	L78	V343	4206.9
V323	M81	L77	4375.3
V323	M81	L78	3834.2
V323	M81	M109	3256.6
V323	L89	L58	2985.5
V323	L89	L78	4614.2
V323	L89	M109	4139.7
V323	L89	L141	3595.3
V323	L89	V343	3328.2
V323	M109	L78	3233.9
V323	M109	M81	2967.4
V323	M109	L89	3752.4
V323	M109	V343	3444.2
V323	L141	L77	3041.1
V323	L141	L89	3502.7
V323	L141	I144	4023.9
V323	I144	L141	3265.3
V323	I144	L174	2513.5

First Res	Second Res	Third Res	Scaled Flow
V323	I149	L77	5488.0
V323	I149	L78	5560.8
V323	L174	L77	4451.5
V323	L174	I144	4509.8
V323	V323	L30	78.8
V323	V323	V41	31.0
V323	V323	L58	394.9
V323	V323	L75	126.2
V323	V323	L77	243.7
V323	V323	L78	118.6
V323	V323	M81	128.9
V323	V323	V86	57.7
V323	V323	I87	927.3
V323	V323	L89	131.6
V323	V323	M109	87.9
V323	V323	L141	118.6
V323	V323	I144	47.8
V323	V323	I149	774.5
V323	V323	L174	439.7
V323	V323	I220	21.2
V323	V323	V343	95.9
V323	V323	L357	52.0
V323	V343	L78	3995.1
V323	V343	L89	3079.3
V323	V343	M109	3515.5
V333	I144	L174	2893.7
V333	L174	I144	3166.9
V333	L174	V186	2727.1
V333	M182	V187	3159.7
V333	V186	L174	2672.9
V333	V186	V187	3150.8
V333	V187	M182	3767.0
V333	V187	V186	3493.1
V333	V333	I144	27.4
V333	V333	L174	42.6
V333	V333	M182	27.1
V333	V333	V186	38.7
V333	V333	V187	62.5
V333	V333	L357	41.5
L337	V26	L78	3044.6
L337	L77	L78	3691.8

First Res	Second Res	Third Res	Scaled Flow
L337	L78	V26	3871.1
L337	L78	L77	5089.4
L337	L78	V343	4983.3
L337	L337	V26	75.4
L337	L337	L58	883.2
L337	L337	L77	52.5
L337	L337	L78	196.9
L337	L337	I150	106.7
L337	L337	V343	35.9
L337	V343	L78	3335.9
V343	V26	L170	4412.8
V343	L58	I87	2930.3
V343	L58	L89	2747.2
V343	L58	L337	3825.1
V343	L77	L78	3121.1
V343	L77	M81	4403.1
V343	L77	L141	3595.1
V343	L77	I149	4171.7
V343	L78	L77	9139.2
V343	L78	M81	7183.3
V343	L78	L89	6846.4
V343	L78	L141	6427.2
V343	L78	I149	8148.6
V343	L78	L170	7280.6
V343	M81	L77	4709.5
V343	M81	L78	2623.9
V343	M81	L141	3460.6
V343	M81	I149	3231.1
V343	M81	L170	3406.1
V343	I87	L58	4057.0
V343	I87	V323	4340.2
V343	L89	L58	4585.3
V343	L89	L78	4004.2
V343	L89	M109	3775.2
V343	L89	L141	5547.5
V343	L89	L170	6259.6
V343	M109	L89	4456.5
V343	M109	L170	6087.1
V343	M112	L170	3792.8
V343	L141	L77	2849.4
V343	L141	L78	1739.7

First Res	Second Res	Third Res	Scaled Flow
V343	L141	M81	2564.4
V343	L141	L89	2567.4
V343	L141	L170	2916.2
V343	I149	L77	3884.1
V343	I149	L78	2590.9
V343	I149	M81	2812.6
V343	I149	V323	3222.9
V343	L170	V26	2393.5
V343	L170	L78	1809.8
V343	L170	M81	2317.9
V343	L170	L89	2660.4
V343	L170	M109	2191.5
V343	L170	M112	2676.6
V343	L170	L141	2678.1
V343	V323	I87	3055.2
V343	V323	I149	3461.6
V343	L337	L58	2999.4
V343	V343	V16	50.1
V343	V343	V26	289.0
V343	V343	L58	106.7
V343	V343	L77	95.9
V343	V343	L78	1890.5
V343	V343	M81	126.2
V343	V343	I87	347.8
V343	V343	L89	601.7
V343	V343	M109	912.4
V343	V343	M112	109.0
V343	V343	L116	42.5
V343	V343	L141	33.4
V343	V343	I149	70.5
V343	V343	L170	21.7
V343	V343	V323	95.9
V343	V343	L337	35.9
L357	L174	V323	2596.6
L357	V323	L174	3079.5
L357	L357	V45	63.2
L357	L357	L66	120.2
L357	L357	V86	119.9
L357	L357	L90	111.6
L357	L357	L174	22.7
L357	L357	V186	261.3

First Res	Second Res	Third Res	Scaled Flow
L357	L357	V323	52.0
L357	L357	V333	41.5
L357	L357	V361	125.7
L357	L357	L367	386.7
V361	V361	L90	188.0
V361	V361	I150	31.1
V361	V361	L288	27.1
V361	V361	V293	437.1
V361	V361	L357	125.7
V361	V361	L367	215.8
L367	L367	L90	368.4
L367	L367	V186	56.6
L367	L367	V293	60.7
L367	L367	L357	386.7
L367	L367	V361	215.8

Table S9. Community membership for apo inactive p38γ

module1	16, 26, 30, 33, 41, 53, 58, 75, 77, 78, 81, 87, 89, 109, 112, 116, 119, 137, 141, 144, 149, 161, 169, 170, 182, 215, 323, 337, 343
module2	125, 130, 150, 197, 198, 201, 212, 220, 225, 238, 239, 241, 250, 253, 262, 265, 268, 276, 277, 287, 288, 291, 292, 293, 294, 300
module3	16, 26, 30, 33, 41, 53, 78, 81, 89, 109, 112, 116, 119, 120, 130, 134, 137, 141, 154, 159, 161, 167, 169, 170, 215, 219, 220, 283, 343
module4	26, 58, 66, 75, 77, 78, 81, 86, 87, 89, 90, 109, 141, 144, 149, 169, 170, 174, 182, 186, 187, 323, 343, 357
module5	150, 174, 197, 198, 201, 225, 238, 239, 241, 250, 253, 262, 265, 268, 276, 288, 292, 293, 294
module6	33, 41, 53, 55, 58, 77, 78, 81, 87, 89, 90, 109, 112, 116, 119, 130, 137, 141, 144, 149, 159, 161, 167, 169, 170, 174, 187, 201, 212, 215, 220, 283, 323, 333
module7	30, 41, 58, 75, 77, 78, 81, 86, 87, 89, 109, 119, 141, 144, 149, 170, 174, 220, 323, 343, 357
module8	16, 23, 26, 30, 33, 41, 45, 53, 55, 58, 66, 75, 77, 78, 81, 87, 89, 109, 112, 116, 119, 134, 137, 141, 149, 150, 159, 161, 169, 170, 283, 323, 337, 343, 357
module9	58, 66, 77, 78, 81, 86, 89, 90, 144, 149, 174, 182, 186, 187, 239, 300, 333, 357, 367
module10	116, 119, 120, 130, 137, 141, 144, 154, 159, 161, 167, 169, 212, 215, 219, 283, 292, 293
module11	90, 150, 197, 209, 220, 238, 276, 283, 288, 292, 293, 357, 361, 367
module12	45, 66, 77, 86, 90, 141, 144, 174, 182, 186, 187, 293, 323, 357, 361, 367
module13	26, 58, 75, 81, 87, 149, 182, 343
module14	33, 120, 125, 150, 169, 220, 277, 287, 288, 294, 323
module15	137, 209, 216, 288, 291, 293
module16	78, 87
module17	58, 116, 141
module18	89, 149
module19	77, 109, 170
module20	78, 323
module21	141, 323
module22	137, 170
module23	58, 77
module24	78, 174
module25	141, 144, 149
module26	141, 174
module27	285

Table S10. Methyl 3-residue flow for +ATP inactive p38γ

First Res	Second Res	Third Res	Scaled Flow
V16	V16	V26	972.2
V16	V16	V33	605.2
V16	V16	L77	500.6
V16	V16	L78	1535.8
V16	V16	M81	122.7
V16	V16	I87	464.3
V16	V16	L89	1452.5
V16	V16	M109	191.4
V16	V16	M112	220.8
V16	V16	I144	105.2
V16	V16	L170	76.6
V16	V16	L174	847.3
V16	V16	L337	813.3
V16	V26	L77	5494.1
V16	V26	I87	5331.1
V16	V26	L170	8183.6
V16	V26	L337	5170.7
V16	L77	V26	4249.1
V16	L77	L78	4033.8
V16	L77	M81	6067.7
V16	L77	L89	4406.7
V16	L77	I144	5591.2
V16	L77	L174	4911.9
V16	L78	L77	6521
V16	L78	M81	8244.2
V16	L78	I144	8079.4
V16	L78	L337	6715.2
V16	M81	L77	4009.4
V16	M81	L78	3369.8
V16	M81	L89	2874.6
V16	M81	I144	3781
V16	M81	L174	3301.3
V16	I87	V26	4006.2
V16	I87	L89	4022.3
V16	I87	M112	4961.3
V16	L89	L77	6914.3
V16	L89	M81	6825.6
V16	L89	I87	6495.2
V16	L89	M112	7131.9
V16	L89	L174	6330
V16	M112	I87	3942.1
V16	M112	L89	3509.3
V16	M112	L174	3670.2
V16	I144	L77	3557.2
V16	I144	L78	3179.7
V16	I144	M81	3640.4
V16	L170	V26	3736.3
V16	L170	L337	3084
V16	L174	L77	6003.6
V16	L174	M81	6106.5
V16	L174	L89	4931.1
V16	L174	M112	5810.5
V16	L337	V26	4792.3
V16	L337	L78	4977.8
V16	L337	L170	6260.4
V23	V23	V45	97.3
V23	V23	V134	237
V23	V23	I276	527.7
V23	V23	L277	205.7
V23	I276	L277	4855.1
V23	L277	I276	3617
V26	V26	V16	972.2
V26	V26	L30	76.6
V26	V26	V53	142.7
V26	V26	L75	237
V26	V26	L77	2456.8
V26	V26	L78	972.2
V26	V26	M81	153.8
V26	V26	V86	916.4
V26	V26	I87	2128.9
V26	V26	L89	2128.9
V26	V26	M112	562.2
V26	V26	I144	165.5
V26	V26	I149	1225.9
V26	V26	L170	3940.5
V26	V26	L174	1275.3
V26	V26	L337	2767.5
V26	V53	M112	3324

First Res	Second Res	Third Res	Scaled Flow
V26	L77	L78	6410.9
V26	L77	M81	10309.9
V26	L77	I144	8929.4
V26	L77	L174	6603
V26	L78	L77	3816
V26	L78	M81	6814.6
V26	L78	I144	6466.2
V26	L78	L337	4386
V26	M81	L77	3321.8
V26	M81	L78	3688.7
V26	M81	L89	2717.1
V26	M81	I144	3740.2
V26	M81	L174	3176.9
V26	L89	M81	7622.7
V26	L89	M112	6682.5
V26	L89	L174	6382.2
V26	M112	V53	5031.8
V26	M112	L89	3536.8
V26	I144	L77	2933.3
V26	I144	L78	3568.7
V26	I144	M81	3813.4
V26	L174	L77	4474.3
V26	L174	M81	6681.4
V26	L174	L89	4784.4
V26	L337	L78	8036.3
L30	V26	L77	4222
L30	L30	V26	76.6
L30	L30	L77	68.6
L30	L30	M291	70.7
L30	L77	V26	4127.7
V33	V16	L89	5456.2
V33	V33	V16	605.2
V33	V33	L89	98.4
V33	V33	M109	305.1
V33	V33	L174	157.2
V33	L89	V16	3198.9
V33	L89	L174	4515.1
V33	L174	L89	5091.1
V41	V41	L119	527.7
V45	V45	V23	97.3
V45	V45	V300	132.4

First Res	Second Res	Third Res	Scaled Flow
V53	V26	L77	4451.9
V53	V26	I87	3835.1
V53	V26	L89	3515.8
V53	V26	L174	3598.3
V53	V53	V26	142.7
V53	V53	L77	133.9
V53	V53	V86	598.6
V53	V53	I87	435.2
V53	V53	L89	863.5
V53	V53	M112	1373.2
V53	V53	L116	1452.5
V53	V53	V161	2494.4
V53	V53	I169	124.1
V53	V53	L174	256.9
V53	V53	M219	3180.2
V53	L77	V26	4391.6
V53	L77	V86	3649.9
V53	L77	L89	3966.7
V53	L77	L174	4529.9
V53	V86	L77	5731
V53	I87	V26	5310.1
V53	I87	L89	4404.6
V53	L89	V26	6291.8
V53	L89	L77	7196.1
V53	L89	I87	5692.8
V53	L89	L174	6743.3
V53	M112	L174	6494.6
V53	V161	I169	8068.3
V53	I169	V161	2447.6
V53	L174	V26	4237.6
V53	L174	L77	5408
V53	L174	L89	4437.6
V53	L174	M112	3446.1
L58	L58	M81	70.7
L66	L66	I197	598.6
L66	L66	L225	237
L66	L66	V241	407.7
L66	L66	L265	173
L66	L66	L268	272.3
L66	L66	I276	70.7
L66	L66	L288	153.8

First Res	Second Res	Third Res	Scaled Flow
L66	L66	L292	495.1
L66	I197	L225	5033.2
L66	I197	V241	5291.1
L66	I197	L265	6754.8
L66	I197	L268	5690.2
L66	L225	I197	3728.1
L66	L225	V241	4790.5
L66	L225	L288	4550.9
L66	L225	L292	4740.7
L66	V241	I197	4628.4
L66	V241	L225	5657.4
L66	V241	L292	5030.4
L66	L265	I197	4637.4
L66	L265	L268	4647.2
L66	L268	I197	4389.1
L66	L268	L265	5221.3
L66	L288	L225	4043.8
L66	L288	L292	4721.8
L66	L292	L225	5979.1
L66	L292	V241	5372.4
L66	L292	L288	6702.1
L75	V26	L89	4770.6
L75	V26	L170	5627.7
L75	V26	L337	5053.8
L75	L75	V26	237
L75	L75	L89	122.7
L75	L75	L170	83.1
L75	L75	L337	122.7
L75	L89	V26	4002.1
L75	L170	V26	4296.9
L75	L170	L337	3775.6
L75	L337	V26	4239.7
L75	L337	L170	4148.3
L77	V26	L170	9463.4
L77	V53	M112	3350.8
L77	V53	L116	3739.7
L77	V53	V161	4324.6
L77	L77	V16	500.6
L77	L77	V26	2456.8
L77	L77	L30	68.6
L77	L77	V53	133.9

First Res	Second Res	Third Res	Scaled Flow
L77	L77	L78	2798.1
L77	L77	M81	3022.2
L77	L77	V86	2095.7
L77	L77	I87	644.1
L77	L77	L89	3518
L77	L77	M112	500.6
L77	L77	L116	223.3
L77	L77	I144	1986.2
L77	L77	I149	550.7
L77	L77	V161	173.6
L77	L77	L167	266
L77	L77	L170	373.3
L77	L77	L174	3647.1
L77	L77	L337	1349.8
L77	L78	L337	7244.4
L77	I87	L89	3272.1
L77	I87	M112	4649.1
L77	L89	I87	8734.5
L77	L89	M112	8842.5
L77	M112	V53	4947.6
L77	M112	I87	4242
L77	M112	L89	3022.6
L77	M112	L116	4673.6
L77	L116	V53	4288.2
L77	L116	M112	3629.5
L77	V161	V53	4627.5
L77	L170	V26	3949
L77	L337	L78	4578.8
L78	V26	I87	5400.6
L78	V26	L170	7349.9
L78	L77	V86	9764.6
L78	L77	L89	6498.4
L78	L78	V16	1535.8
L78	L78	V26	972.2
L78	L78	L77	2798.1
L78	L78	M81	2627.8
L78	L78	V86	165.5
L78	L78	I87	435.2
L78	L78	L89	1452.5
L78	L78	M112	254.1
L78	L78	L141	142.7

First Res	Second Res	Third Res	Scaled Flow
L78	L78	I144	2245.1
L78	L78	L167	637
L78	L78	L170	191.4
L78	L78	L174	2152.5
L78	L78	V285	527.7
L78	L78	L337	4139.3
L78	V86	L77	2907.8
L78	V86	I87	3660.3
L78	V86	L89	2943.1
L78	I87	V26	3970.4
L78	I87	V86	4875.9
L78	I87	L89	3988.8
L78	I87	M112	4803.8
L78	L89	L77	4254.9
L78	L89	V86	6471.1
L78	L89	I87	6583.9
L78	L89	M112	6954.6
L78	L89	L174	4867.4
L78	M112	I87	4061.6
L78	M112	L89	3562.5
L78	M112	L174	3133.5
L78	L170	V26	4218.2
L78	L170	L337	2432.5
L78	L174	L89	6152.4
L78	L174	M112	7732
L78	L337	L170	11336.1
M81	V16	L78	2620.6
M81	V16	L89	2874.6
M81	V26	L77	3001
M81	V26	I87	4263.9
M81	V26	L89	3278.4
M81	V26	L337	3978
M81	L77	V26	10941.9
M81	L77	L89	6704
M81	L78	V16	9028.2
M81	L78	L337	8952.6
M81	M81	V16	122.7
M81	M81	V26	153.8
M81	M81	L58	70.7
M81	M81	L77	3022.2
M81	M81	L78	2627.8

First Res	Second Res	Third Res	Scaled Flow
M81	M81	V86	598.6
M81	M81	I87	178.1
M81	M81	L89	1452.5
M81	M81	M112	598.6
M81	M81	L116	407.7
M81	M81	I144	1157.6
M81	M81	L167	272.3
M81	M81	L174	1782
M81	M81	V285	205.7
M81	M81	L337	598.6
M81	V86	I87	5171.8
M81	I87	V26	4432.9
M81	I87	V86	3538.9
M81	I87	L89	3587.7
M81	I87	M112	3695.8
M81	L89	V16	6825.6
M81	L89	V26	7349.5
M81	L89	L77	4122
M81	L89	I87	7736.3
M81	L89	M112	5802.2
M81	L89	L174	5177.7
M81	M112	I87	5401.2
M81	M112	L89	3932.4
M81	L174	L89	5818.6
M81	L337	V26	6044
M81	L337	L78	4182
V86	V26	L170	5739.8
V86	V26	L337	6248.1
V86	V53	V161	4439.1
V86	V53	M219	6315.8
V86	L77	L78	9174
V86	L77	M81	7197.1
V86	L77	L89	5890.8
V86	L77	I144	8263.1
V86	L77	L174	6766.7
V86	L78	L77	3359
V86	L78	M81	3968.4
V86	L78	L89	2943.1
V86	L78	I144	4398.4
V86	L78	L174	3517.3
V86	L78	L337	4799

First Res	Second Res	Third Res	Scaled Flow
V86	M81	L77	3926.9
V86	M81	L78	5913.7
V86	V86	V26	916.4
V86	V86	V53	598.6
V86	V86	L77	2095.7
V86	V86	L78	165.5
V86	V86	M81	598.6
V86	V86	I87	1623.2
V86	V86	L89	1452.5
V86	V86	M112	813.3
V86	V86	L116	334
V86	V86	I144	165.5
V86	V86	I149	765.6
V86	V86	V161	916.4
V86	V86	I169	248.1
V86	V86	L170	813.3
V86	V86	L174	982.9
V86	V86	M219	139
V86	V86	L337	272.3
V86	L89	L77	4742.5
V86	L89	L78	6471.1
V86	L89	L174	6108.4
V86	L116	M219	5011.9
V86	I144	L77	3025.5
V86	I144	L78	4398.4
V86	V161	V53	5260.4
V86	V161	M219	6252.3
V86	L170	V26	5458.4
V86	L170	L337	5190
V86	L174	L77	4523.6
V86	L174	L78	6421.9
V86	L174	L89	5072.3
V86	M219	V53	4061.1
V86	M219	L116	3926.7
V86	M219	V161	3392.6
V86	L337	V26	4067
V86	L337	L78	5516.2
V86	L337	L170	3552.4
I87	V26	L77	6520.3
I87	V26	L170	7047.1
I87	V26	L337	7222.4

First Res	Second Res	Third Res	Scaled Flow
I87	V53	V161	5561.3
I87	L77	V26	3639.3
I87	L77	L78	5344.5
I87	L77	M81	6188.8
I87	L77	L89	3911.7
I87	L77	L174	5263.7
I87	L78	L77	4635.6
I87	L78	M81	5444.9
I87	L78	L174	4178.3
I87	L78	L337	5321
I87	M81	L77	4108.9
I87	M81	L78	4167.9
I87	M81	L89	2580.4
I87	M81	L174	3506.7
I87	I87	V16	464.3
I87	I87	V26	2128.9
I87	I87	V53	435.2
I87	I87	L77	644.1
I87	I87	L78	435.2
I87	I87	M81	178.1
I87	I87	V86	1623.2
I87	I87	L89	2767.5
I87	I87	M112	1912.2
I87	I87	L116	527.7
I87	I87	I149	237
I87	I87	V161	132.4
I87	I87	L167	165.5
I87	I87	L170	972.2
I87	I87	L174	751
I87	I87	L337	527.7
I87	L89	L77	8455.6
I87	L89	M81	8401.4
I87	L89	L174	8348.5
I87	V161	V53	3940.3
I87	L170	V26	4640.4
I87	L174	L77	5587.7
I87	L174	L78	5113.8
I87	L174	M81	5606.8
I87	L174	L89	4099.8
I87	L337	V26	3736.9
I87	L337	L78	5686.8

First Res	Second Res	Third Res	Scaled Flow
L89	V26	L170	8908.9
L89	V26	L337	7338.8
L89	V53	V161	6426.8
L89	V53	M219	7312.2
L89	L77	I144	10590.7
L89	L78	I144	7107.7
L89	L78	L337	7309.2
L89	L89	V16	1452.5
L89	L89	V26	2128.9
L89	L89	V33	98.4
L89	L89	V53	863.5
L89	L89	L75	122.7
L89	L89	L77	3518
L89	L89	L78	1452.5
L89	L89	M81	1452.5
L89	L89	V86	1452.5
L89	L89	I87	2767.5
L89	L89	M109	220.8
L89	L89	M112	2366.9
L89	L89	L116	637
L89	L89	I144	205.7
L89	L89	I149	637
L89	L89	V161	165.5
L89	L89	L167	142.7
L89	L89	L170	357.2
L89	L89	L174	3791.5
L89	L89	M219	94.6
L89	L89	L337	495.1
L89	L116	M219	6069.9
L89	I144	L77	2739.9
L89	I144	L78	3428.6
L89	V161	V53	3732.7
L89	V161	M219	4474.9
L89	L170	V26	4043.9
L89	L170	L337	4010.8
L89	M219	V53	3701
L89	M219	L116	3468
L89	M219	V161	3899.6
L89	L337	V26	3712.9
L89	L337	L78	4627.8
L89	L337	L170	4470.4

First Res	Second Res	Third Res	Scaled Flow
L90	L90	V285	133.9
M109	V16	L89	3952.9
M109	L89	V16	4112.7
M109	L89	M112	3957.4
M109	L89	L174	4991.3
M109	M109	V16	191.4
M109	M109	V33	305.1
M109	M109	L89	220.8
M109	M109	M112	677.6
M109	M109	I149	178.1
M109	M109	L174	248.1
M109	M109	L337	83.1
M109	M112	L89	5712
M109	M112	L174	5385.8
M109	L174	L89	5174.4
M109	L174	M112	3868.3
M112	V16	L78	4030.3
M112	V26	L77	4877.8
M112	V26	I149	5299.5
M112	V26	L337	6003.3
M112	V53	V161	5223.1
M112	V53	M219	8665.5
M112	L77	V26	4693.6
M112	L77	L78	5466
M112	L77	M81	4931.8
M112	L77	V86	4181.5
M112	L77	L89	3962.3
M112	L77	L174	4146.4
M112	L77	L337	4890.3
M112	L78	V16	4195.6
M112	L78	L77	4393.1
M112	L78	M81	4225.8
M112	L78	L174	3227.8
M112	L78	L337	5512.8
M112	M81	L77	5244.5
M112	M81	L78	5591.1
M112	V86	L77	5011
M112	L89	L77	8359.1
M112	L89	L174	5546.1
M112	M112	V16	220.8
M112	M112	V26	562.2

First Res	Second Res	Third Res	Scaled Flow
M112	M112	V53	1373.2
M112	M112	L77	500.6
M112	M112	L78	254.1
M112	M112	M81	598.6
M112	M112	V86	813.3
M112	M112	I87	1912.2
M112	M112	L89	2366.9
M112	M112	M109	677.6
M112	M112	L116	1452.5
M112	M112	I149	76.6
M112	M112	V161	916.4
M112	M112	L167	334
M112	M112	I169	167.3
M112	M112	L174	2040.3
M112	M112	M219	87.4
M112	M112	L337	142.7
M112	L116	M219	7957.9
M112	I149	V26	3010.3
M112	V161	V53	4319.4
M112	V161	M219	6655.6
M112	L174	L77	7943
M112	L174	L78	7693.3
M112	L174	L89	5036
M112	M219	V53	3470.7
M112	M219	L116	3095.4
M112	M219	V161	3223.4
M112	L337	V26	3965.8
M112	L337	L77	3357.4
M112	L337	L78	4709
L116	V53	V161	5471.8
L116	V53	M219	4516.8
L116	L77	M81	4509.3
L116	L77	V86	4222.4
L116	L77	L89	4434.1
L116	L77	L174	4826.1
L116	M81	L77	5401.2
L116	M81	L174	4485.8
L116	V86	L77	4745.2
L116	V86	I87	3929.4
L116	I87	V86	4574.2
L116	I87	L89	4799

First Res	Second Res	Third Res	Scaled Flow
L116	L89	L77	6214.6
L116	L89	I87	5141.2
L116	L89	M112	3963.4
L116	L89	L174	6026.9
L116	M112	L89	5713.6
L116	M112	L174	6305.9
L116	L116	V53	1452.5
L116	L116	L77	223.3
L116	L116	M81	407.7
L116	L116	V86	334
L116	L116	I87	527.7
L116	L116	L89	637
L116	L116	M112	1452.5
L116	L116	L125	165.5
L116	L116	V161	813.3
L116	L116	L167	291.7
L116	L116	L174	326.6
L116	L116	M216	105.2
L116	L116	M219	2095.7
L116	L125	M216	4480.1
L116	V161	V53	4179.1
L116	L174	L77	5400.3
L116	L174	M81	4190.6
L116	L174	L89	4811.8
L116	L174	M112	3492.4
L116	M216	L125	3994.3
L116	M219	V53	5610.5
L119	L119	V41	527.7
M120	M120	I130	94
M120	M120	M137	68
M120	M120	L159	68
M120	M120	V361	68.8
M120	M137	V361	3748.1
M120	V361	M137	3767.7
L125	L116	M219	4659.4
L125	L125	L116	165.5
L125	L125	M137	254.1
L125	L125	L167	562.2
L125	L125	I209	527.7
L125	L125	I215	110.6
L125	L125	M216	2018

First Res	Second Res	Third Res	Scaled Flow
L125	L125	M219	71.5
L125	L125	V361	239.6
L125	M137	V361	4101.3
L125	M219	L116	3803.6
L125	V361	M137	4043.2
I130	I130	M120	94
I130	I130	L154	272.3
I130	I130	M216	237
I130	I130	I238	495.1
I130	I130	I276	237
I130	I130	L277	1373.2
I130	I130	L288	272.3
V134	V134	V23	237
V134	V134	I276	220.8
V134	V134	L277	113.7
V134	V134	V285	1623.2
V134	I276	L277	4409.4
V134	L277	I276	3703
M137	M137	M120	68
M137	M137	L125	254.1
M137	M137	L159	220.8
M137	M137	L167	272.3
M137	M137	I215	71.5
M137	M137	V361	1468.6
L141	L78	I144	3797.4
L141	L78	L337	5202.4
L141	L141	L78	142.7
L141	L141	I144	527.7
L141	L141	L167	237
L141	L141	V285	122.7
L141	L141	L337	70.7
L141	I144	L78	5619.4
L141	L337	L78	4391
I144	V16	L78	2678.2
I144	V16	L89	3642.2
I144	V26	L77	3261.5
I144	V26	L89	4254.9
I144	V26	I149	4079.8
I144	V26	L337	3835.4
I144	L77	V26	8607.9
I144	L77	M81	5834.7
I144	L77	V86	8201.6
I144	L77	L89	9224.4
I144	L77	L174	7800.4
I144	L78	V16	8563.5
I144	L78	L89	7325
I144	L78	L174	6710.6
I144	L78	L337	7580.6
I144	M81	L77	4338.8
I144	V86	L77	3107.6
I144	V86	L89	3895.7
I144	L89	V16	4332.8
I144	L89	V26	4512.8
I144	L89	L77	3707
I144	L89	L78	2725.3
I144	L89	V86	4131.9
I144	L89	L174	4558.9
I144	I144	V16	105.2
I144	I144	V26	165.5
I144	I144	L77	1986.2
I144	I144	L78	2245.1
I144	I144	M81	1157.6
I144	I144	V86	165.5
I144	I144	L89	205.7
I144	I144	L141	527.7
I144	I144	I149	89.9
I144	I144	L167	407.7
I144	I144	L174	516.8
I144	I144	V285	97.3
I144	I144	L337	863.5
I144	I149	V26	3503.3
I144	L174	L77	4188.4
I144	L174	L78	3335.9
I144	L174	L89	6091.3
I144	L337	V26	6603.5
I144	L337	L78	4578.3
I149	V26	L77	5713.5
I149	V26	I87	6450.1
I149	V26	L170	5860.8
I149	V26	L337	7684.3
I149	L77	V26	4105.2
I149	L77	V86	4295.6

First Res	Second Res	Third Res	Scaled Flow
I149	L77	L89	5194.5
I149	L77	I144	5823.9
I149	L77	L174	4999.1
I149	L77	L337	5096.4
I149	V86	L77	4855.6
I149	V86	I87	5239.5
I149	I87	V26	3529
I149	I87	V86	3529.6
I149	I87	L89	4202.4
I149	I87	M112	4870.2
I149	L89	L77	5465.7
I149	L89	I87	5806.9
I149	L89	M112	6404.2
I149	L89	L174	5194.8
I149	M112	I87	3650
I149	M112	L89	3473.5
I149	M112	L174	3242.2
I149	I144	L77	3449.9
I149	I149	V26	1225.9
I149	I149	L77	550.7
I149	I149	V86	765.6
I149	I149	I87	237
I149	I149	L89	637
I149	I149	M109	178.1
I149	I149	M112	76.6
I149	I149	I144	89.9
I149	I149	L170	1030.8
I149	I149	L174	847.3
I149	I149	L337	122.7
I149	L170	V26	5403.2
I149	L170	L337	6295.2
I149	L174	L77	5906.9
I149	L174	L89	5833.7
I149	L174	M112	6713
I149	L337	V26	3527
I149	L337	L77	3255.6
I149	L337	L170	3134.1
I150	I150	M262	71.5
L154	L154	I130	272.3
L154	L154	I209	178.1
L154	L154	M216	132.4

First Res	Second Res	Third Res	Scaled Flow
L154	L154	I220	527.7
L154	L154	L283	916.4
L154	L154	L288	70.7
L154	L154	M291	1092.6
L154	L154	V361	68.6
L154	I220	L283	4714.7
L154	I220	M291	4343.8
L154	L283	I220	5847.8
L154	M291	I220	5827.9
L159	M137	V361	4578
L159	L159	M120	68
L159	L159	M137	220.8
L159	L159	L167	113.7
L159	L159	L287	76.6
L159	L159	V361	68.6
L159	V361	M137	3422.2
V161	L77	V86	3557.8
V161	L77	L89	4894.1
V161	L77	L174	4496.9
V161	V86	L77	6186.6
V161	V86	I87	6014.1
V161	V86	L89	5631.3
V161	I87	V86	3214.4
V161	I87	L89	4432
V161	I87	M112	3359.7
V161	L89	L77	4819.1
V161	L89	V86	3188.9
V161	L89	I87	4695.8
V161	L89	M112	3650.7
V161	L89	L174	4496.6
V161	M112	I87	6286
V161	M112	L89	6446.8
V161	M112	L174	5267.6
V161	V161	V53	2494.4
V161	V161	L77	173.6
V161	V161	V86	916.4
V161	V161	I87	132.4
V161	V161	L89	165.5
V161	V161	M112	916.4
V161	V161	L116	813.3
V161	V161	L167	105.2

First Res	Second Res	Third Res	Scaled Flow
V161	V161	I169	1204.5
V161	V161	L174	425.9
V161	V161	M219	1933.3
V161	L174	L77	5872.4
V161	L174	L89	5963.4
V161	L174	M112	3956
L167	L77	L78	4295.3
L167	L77	M81	4868
L167	L77	L89	5379.6
L167	L77	I144	4174.5
L167	L77	L174	4738.1
L167	L77	L337	4508.8
L167	L78	L77	5723.7
L167	L78	M81	5617.7
L167	L78	L89	5256.1
L167	L78	I144	5050.1
L167	L78	L174	4805.4
L167	L78	L337	7098.7
L167	M81	L77	4888.5
L167	M81	L78	4233.5
L167	M81	L89	4411.8
L167	M81	L174	3941.6
L167	I87	L89	4665.1
L167	I87	M112	3937.1
L167	L89	L77	4521.7
L167	L89	L78	3315.4
L167	L89	M81	3692.7
L167	L89	I87	4488.2
L167	L89	M112	4049.9
L167	L89	L174	4320
L167	M112	I87	4815.1
L167	M112	L89	5148.2
L167	M112	L116	4173.1
L167	M112	L174	4221.4
L167	L116	M112	4003.3
L167	L125	M216	5919.9
L167	M137	V361	4129.8
L167	I144	L77	4754
L167	I144	L78	4316
L167	V161	I169	3171.4
L167	L167	L77	266

First Res	Second Res	Third Res	Scaled Flow
L167	L167	L78	637
L167	L167	M81	272.3
L167	L167	I87	165.5
L167	L167	L89	142.7
L167	L167	M112	334
L167	L167	L116	291.7
L167	L167	L125	562.2
L167	L167	M137	272.3
L167	L167	L141	237
L167	L167	I144	407.7
L167	L167	L159	113.7
L167	L167	V161	105.2
L167	L167	I169	469.4
L167	L167	L174	500.6
L167	L167	M216	83.1
L167	L167	L337	97.3
L167	L167	V361	248.1
L167	I169	V161	4875.1
L167	L174	L77	5800.8
L167	L174	L78	4415
L167	L174	M81	4805.4
L167	L174	L89	6292.5
L167	L174	M112	4837
L167	M216	L125	3425.9
L167	L337	L77	3446.5
L167	L337	L78	4072.1
L167	V361	M137	4029.8
I169	V53	M112	3761.1
I169	V53	V161	3444.6
I169	M112	V53	4062.4
I169	M112	L174	4345.4
I169	V161	V53	7420.5
I169	I169	V53	124.1
I169	I169	V86	248.1
I169	I169	M112	167.3
I169	I169	V161	1204.5
I169	I169	L167	469.4
I169	I169	L174	151.4
I169	I169	I215	76.8
I169	I169	L283	87.4
I169	I169	L337	110.6

First Res	Second Res	Third Res	Scaled Flow
I169	L174	M112	4244.5
L170	V16	V26	2032.3
L170	V16	L78	3549.5
L170	V16	L89	3299.2
L170	V26	V16	11273.9
L170	V26	L77	10652.9
L170	V26	L89	10169
L170	V26	L174	11075.9
L170	L77	V26	2948.9
L170	L77	L78	5312.7
L170	L77	V86	4004.8
L170	L77	L89	5217.6
L170	L77	L174	5897.5
L170	L78	V16	4461.8
L170	L78	L77	4349.1
L170	L78	L89	3725.1
L170	L78	L174	4586.7
L170	L78	L337	3978.7
L170	V86	L77	5294.3
L170	I87	L89	6073.9
L170	L89	V16	4981.3
L170	L89	V26	2767.8
L170	L89	L77	5130.3
L170	L89	L78	4474.2
L170	L89	I87	4186.9
L170	L89	L174	5881.4
L170	L170	V16	76.6
L170	L170	V26	3940.5
L170	L170	L75	83.1
L170	L170	L77	373.3
L170	L170	L78	191.4
L170	L170	V86	813.3
L170	L170	I87	972.2
L170	L170	L89	357.2
L170	L170	I149	1030.8
L170	L170	L174	124.1
L170	L170	L337	1623.2
L170	L174	V26	2247.3
L170	L174	L77	4322.8
L170	L174	L78	4106.9
L170	L174	L89	4384.3

First Res	Second Res	Third Res	Scaled Flow
L170	L337	L78	8936.3
L174	V26	L170	8520
L174	V26	L337	5411.6
L174	V53	L116	3937.7
L174	V53	V161	4370.5
L174	L78	I144	6724
L174	L78	L337	7431.6
L174	I87	L89	3199.6
L174	L89	I87	8670.3
L174	L116	V53	4232.1
L174	I144	L78	3428.6
L174	V161	V53	5115.6
L174	L170	V26	3845.8
L174	L170	L337	3215
L174	L174	V16	847.3
L174	L174	V26	1275.3
L174	L174	V33	157.2
L174	L174	V53	256.9
L174	L174	L77	3647.1
L174	L174	L78	2152.5
L174	L174	M81	1782
L174	L174	V86	982.9
L174	L174	I87	751
L174	L174	L89	3791.5
L174	L174	M109	248.1
L174	L174	M112	2040.3
L174	L174	L116	326.6
L174	L174	I144	516.8
L174	L174	I149	847.3
L174	L174	V161	425.9
L174	L174	L167	500.6
L174	L174	I169	151.4
L174	L174	L170	124.1
L174	L174	L337	873.1
L174	L337	V26	4550.5
L174	L337	L78	4638
L174	L337	L170	5989.1
M182	M182	V186	142.7
M182	M182	V187	720.4
M182	V186	V187	3751
M182	V187	V186	6235.6

First Res	Second Res	Third Res	Scaled Flow
V186	V186	M182	142.7
V186	V186	V187	2494.4
V186	V186	V333	562.2
V187	V187	M182	720.4
V187	V187	V186	2494.4
V187	V187	V333	464.3
I197	I197	L66	598.6
I197	I197	L198	3226.2
I197	I197	L225	1715
I197	I197	I238	291.7
I197	I197	M239	527.7
I197	I197	V241	2767.5
I197	I197	V250	863.5
I197	I197	L253	677.6
I197	I197	M262	926.6
I197	I197	L265	4769.8
I197	I197	L268	2913.6
I197	I197	L288	1225.9
I197	I197	L292	1373.2
I197	I197	V293	1452.5
I197	I197	L294	1030.8
I197	L198	L265	6342.9
I197	L225	I238	7065.4
I197	L225	V241	4532.7
I197	L225	L292	5945.8
I197	I238	L225	3440
I197	I238	V241	2971.3
I197	M239	L294	4394.4
I197	V241	L225	6244.9
I197	V241	I238	8407.9
I197	V241	L292	6484.5
I197	L265	L198	9819.4
I197	L288	L292	6083.8
I197	L288	V293	4761.9
I197	L292	L225	5268.9
I197	L292	V241	4170.8
I197	L292	L288	6439.2
I197	V293	L288	5189.7
I197	L294	M239	5740.9
L198	L198	I197	3226.2
L198	L198	L225	720.4

First Res	Second Res	Third Res	Scaled Flow
L198	L198	I238	105.2
L198	L198	M239	813.3
L198	L198	V241	1030.8
L198	L198	V250	132.4
L198	L198	L253	272.3
L198	L198	M262	1275.3
L198	L198	L265	7993.3
L198	L198	L268	2366.9
L198	L198	L288	464.3
L198	L198	L292	916.4
L198	L198	V293	765.6
L198	L198	L294	495.1
L198	L225	I238	6307.7
L198	L225	V241	5120
L198	L225	L288	4821.1
L198	L225	L292	5320.2
L198	I238	L225	3516.3
L198	I238	V241	3269.4
L198	M239	L253	5276.1
L198	M239	L294	5484.9
L198	V241	L225	5954.3
L198	V241	I238	6820.6
L198	V241	L292	5476.1
L198	V241	L294	5526.4
L198	L253	M239	3611.3
L198	L288	L225	4104.5
L198	L288	L292	5311.4
L198	L288	V293	4615.8
L198	L292	L225	5874.2
L198	L292	V241	5199.1
L198	L292	L288	6888.4
L198	V293	L288	5554.6
L198	L294	M239	4547
L198	L294	V241	4136.4
M201	M201	L253	76.6
I209	L125	M216	5169.8
I209	I209	L125	527.7
I209	I209	L154	178.1
I209	I209	I215	398.9
I209	I209	M216	220.8
I209	I209	I220	562.2

First Res	Second Res	Third Res	Scaled Flow
I209	I209	M262	161.3
I209	I209	L283	165.5
I209	I209	L288	76.6
I209	I209	M291	312.2
I209	I209	V293	464.3
I209	I209	L357	982.9
I209	M216	L125	3928.7
I209	I220	L283	6123.2
I209	I220	M291	5429.7
I209	L283	I220	4204.4
I209	L283	M291	3711.3
I209	L288	V293	3758.7
I209	M291	I220	4465.5
I209	M291	L283	4445.3
I209	V293	L288	6186.7
V212	V212	I220	142.7
V212	V212	V250	83.1
V212	V212	L283	97.3
V212	V212	M291	813.3
V212	I220	L283	4839.5
V212	I220	M291	3791.7
V212	L283	I220	4401.1
V212	L283	M291	3073.5
V212	M291	I220	6618.3
V212	M291	L283	5899.2
I215	I215	L125	110.6
I215	I215	M137	71.5
I215	I215	I169	76.8
I215	I215	I209	398.9
I215	I215	M291	94.6
M216	L116	M219	4081.9
M216	I130	L277	4424
M216	M216	L116	105.2
M216	M216	L125	2018
M216	M216	I130	237
M216	M216	L154	132.4
M216	M216	L167	83.1
M216	M216	I209	220.8
M216	M216	M219	139
M216	M216	L277	97.3
M216	M216	V361	77.5

First Res	Second Res	Third Res	Scaled Flow
M216	M219	L116	4387.1
M216	L277	I130	3506.7
M219	V53	M112	10474.9
M219	V86	L89	4091.3
M219	L89	V86	3720.8
M219	L89	M112	4271.8
M219	M112	V53	2397.8
M219	M112	L89	4192.4
M219	M112	L116	2635.4
M219	L116	M112	8415.8
M219	L125	M216	3821.9
M219	M216	L125	4486.2
M219	M219	V53	3180.2
M219	M219	V86	139
M219	M219	L89	94.6
M219	M219	M112	87.4
M219	M219	L116	2095.7
M219	M219	L125	71.5
M219	M219	V161	1933.3
M219	M219	M216	139
I220	I220	L154	527.7
I220	I220	I209	562.2
I220	I220	V212	142.7
I220	I220	L283	3226.2
I220	I220	L288	122.7
I220	I220	M291	2767.5
I220	I220	V293	76.6
I220	I220	L357	155.5
I220	L288	V293	4698.4
I220	V293	L288	4197.4
L225	I197	L198	6542.3
L225	I197	L265	6911.9
L225	I197	L268	7882
L225	L198	I197	4342.2
L225	L198	L265	6410.1
L225	L198	L268	5681
L225	L225	L66	237
L225	L225	I197	1715
L225	L225	L198	720.4
L225	L225	I238	2245.1
L225	L225	M239	562.2

First Res	Second Res	Third Res	Scaled Flow
L225	L225	V241	3750
L225	L225	L265	1016.1
L225	L225	L268	254.1
L225	L225	I276	312.2
L225	L225	L277	813.3
L225	L225	L288	1912.2
L225	L225	L292	3940.5
L225	L225	V293	972.2
L225	L225	L294	1811.2
L225	L225	L367	562.2
L225	M239	L294	3917.5
L225	L265	I197	5359.4
L225	L265	L198	7488.6
L225	L265	L268	6909.2
L225	L268	I197	3684.1
L225	L268	L198	4000.7
L225	L268	L265	4164.8
L225	L288	L292	4783.5
L225	L288	V293	5989.5
L225	L292	L288	8538.8
L225	V293	L288	4221.6
L225	L294	M239	6692.3
I238	I197	L198	5525.6
I238	I197	L225	2945.5
I238	I197	V241	3564.7
I238	I197	L265	5985.7
I238	I197	V293	4545.2
I238	L198	I197	4198.9
I238	L198	L265	5410.7
I238	L225	I197	7157
I238	L225	V241	5467.6
I238	L225	L292	7191.6
I238	I238	I130	495.1
I238	I238	I197	291.7
I238	I238	L198	105.2
I238	I238	L225	2245.1
I238	I238	M239	254.1
I238	I238	V241	2018
I238	I238	L265	101.7
I238	I238	I276	863.5
I238	I238	L277	1623.2

First Res	Second Res	Third Res	Scaled Flow
I238	I238	L287	165.5
I238	I238	L288	813.3
I238	I238	L292	972.2
I238	I238	V293	122.7
I238	I238	L294	381.7
I238	M239	L294	4539.2
I238	V241	I197	8077.4
I238	V241	L225	5098.9
I238	V241	L292	6501.2
I238	V241	L294	7221.5
I238	L265	I197	4561.6
I238	L265	L198	5426.2
I238	L288	L292	5912.4
I238	L288	V293	6830.5
I238	L292	L225	4571
I238	L292	V241	4431
I238	L292	L288	6379.3
I238	L292	V293	6558.1
I238	V293	I197	3587.3
I238	V293	L288	3766.4
I238	V293	L292	3351.5
I238	L294	M239	5141.9
I238	L294	V241	3465.7
M239	I197	L198	4815.8
M239	I197	V241	5051.8
M239	I197	L265	5478.9
M239	L198	I197	5680.4
M239	L198	L265	6979.6
M239	L225	I238	5287.2
M239	L225	V241	5565.2
M239	L225	L288	5428
M239	L225	L292	5744.2
M239	I238	L225	4088.9
M239	I238	V241	4082.9
M239	I238	L277	4764.7
M239	M239	I197	527.7
M239	M239	L198	813.3
M239	M239	L225	562.2
M239	M239	I238	254.1
M239	M239	V241	464.3
M239	M239	V250	178.1

First Res	Second Res	Third Res	Scaled Flow
M239	M239	L253	1715
M239	M239	L265	708.2
M239	M239	L277	76.6
M239	M239	L288	153.8
M239	M239	L292	407.7
M239	M239	V293	677.6
M239	M239	L294	2767.5
M239	V241	I197	4831.6
M239	V241	L225	5203.2
M239	V241	I238	4936
M239	V241	L292	5301.4
M239	V241	L294	3241.5
M239	L265	I197	6181.6
M239	L265	L198	6676.1
M239	L277	I238	3499.6
M239	L277	L288	3661.2
M239	L288	L225	3655.6
M239	L288	L277	4340.8
M239	L288	L292	4811.4
M239	L288	V293	3968.6
M239	L292	L225	5141.4
M239	L292	V241	5075.2
M239	L292	L288	6394.6
M239	L292	V293	4137.4
M239	V293	L288	6318.7
M239	V293	L292	4956.5
M239	L294	V241	7903.4
V241	I197	L198	7039.5
V241	I197	L265	8231.9
V241	I197	L268	8439.7
V241	L198	I197	3943.9
V241	L198	L265	6954.8
V241	L198	L268	5526.4
V241	L225	L277	10152.9
V241	I238	L277	8723.5
V241	M239	L294	3593.9
V241	V241	L66	407.7
V241	V241	I197	2767.5
V241	V241	L198	1030.8
V241	V241	L225	3750
V241	V241	I238	2018

First Res	Second Res	Third Res	Scaled Flow
V241	V241	M239	464.3
V241	V241	V250	291.7
V241	V241	L265	1077.4
V241	V241	L268	495.1
V241	V241	I276	165.5
V241	V241	L277	76.6
V241	V241	L287	122.7
V241	V241	L288	1030.8
V241	V241	L292	3393.2
V241	V241	V293	407.7
V241	V241	L294	2366.9
V241	V241	L367	191.4
V241	L265	I197	4758.1
V241	L265	L198	7175.3
V241	L265	L268	6292.4
V241	L268	I197	3539.1
V241	L268	L198	4136.4
V241	L268	L265	4565
V241	I276	L277	4346.5
V241	L277	L225	1928.1
V241	L277	I238	2714.5
V241	L277	I276	3595.7
V241	L277	L288	2991.6
V241	L277	L292	1974.3
V241	L288	L277	6726.2
V241	L288	L292	4610.9
V241	L288	V293	6123.7
V241	L292	L277	9429.1
V241	L292	L288	9794.5
V241	L292	V293	8599.3
V241	L292	L367	10142
V241	V293	L288	4291.7
V241	V293	L292	2837.1
V241	V293	L367	4788.6
V241	L294	M239	7803.2
V241	L367	L292	2669.4
V241	L367	V293	3820.1
V250	I197	L198	7063.6
V250	I197	V241	6083.1
V250	I197	L265	7682
V250	I197	L268	6886.9

First Res	Second Res	Third Res	Scaled Flow
V250	I197	V293	5608.4
V250	L198	I197	3872.1
V250	L198	L265	5533.6
V250	L198	L268	4371.3
V250	M239	L253	3970.7
V250	M239	L294	4631.3
V250	V241	I197	4145.2
V250	V241	L294	4893.5
V250	V250	I197	863.5
V250	V250	L198	132.4
V250	V250	V212	83.1
V250	V250	M239	178.1
V250	V250	V241	291.7
V250	V250	L253	272.3
V250	V250	L265	138.4
V250	V250	L268	132.4
V250	V250	L287	178.1
V250	V250	M291	105.2
V250	V250	V293	153.8
V250	V250	L294	178.1
V250	L253	M239	4475.9
V250	L265	I197	4306.1
V250	L265	L198	5658.4
V250	L265	L268	4767
V250	L268	I197	3775.3
V250	L268	L198	4371.3
V250	L268	L265	4661.9
V250	V293	I197	3194.9
V250	L294	M239	4631.3
V250	L294	V241	4253
L253	I197	L198	6021.1
L253	I197	L265	6124.1
L253	I197	V293	5048.1
L253	L198	I197	4431.9
L253	L198	M262	4114
L253	L198	L265	5719
L253	M239	L294	6865.7
L253	L253	I197	677.6
L253	L253	L198	272.3
L253	L253	M201	76.6
L253	L253	M239	1715

First Res	Second Res	Third Res	Scaled Flow
L253	L253	V250	272.3
L253	L253	M262	193.5
L253	L253	L265	485.3
L253	L253	L287	83.1
L253	L253	V293	205.7
L253	L253	L294	464.3
L253	M262	L198	3743.3
L253	M262	L265	4018.2
L253	L265	I197	5481.9
L253	L265	L198	6955
L253	L265	M262	5370.3
L253	V293	I197	3428.6
L253	L294	M239	3879.5
M262	I197	L198	4870.4
M262	I197	L265	4830.5
M262	I197	L268	4315.8
M262	I197	V293	6078
M262	L198	I197	5646.6
M262	L198	L265	6280
M262	M262	I150	71.5
M262	M262	I197	926.6
M262	M262	L198	1275.3
M262	M262	I209	161.3
M262	M262	L253	193.5
M262	M262	L265	2310.4
M262	M262	L268	1733.9
M262	M262	V293	102.3
M262	L265	I197	8002.6
M262	L265	L198	8973.8
M262	L268	I197	5898.8
M262	V293	I197	3033.5
L265	L225	I238	7010.3
L265	L225	V241	5451.1
L265	L225	L288	5221.4
L265	L225	L292	5689.2
L265	I238	L225	3354.6
L265	I238	V241	3238.2
L265	M239	L294	4981.5
L265	V241	L225	5598.3
L265	V241	I238	6949.7
L265	V241	L292	5477.7

First Res	Second Res	Third Res	Scaled Flow
L265	V241	L294	5166.6
L265	L265	L66	173
L265	L265	I197	4769.8
L265	L265	L198	7993.3
L265	L265	L225	1016.1
L265	L265	I238	101.7
L265	L265	M239	708.2
L265	L265	V241	1077.4
L265	L265	V250	138.4
L265	L265	L253	485.3
L265	L265	M262	2310.4
L265	L265	L268	3371.9
L265	L265	L288	454.8
L265	L265	L292	957.8
L265	L265	V293	587.6
L265	L265	L294	708.2
L265	L288	L225	3820.5
L265	L288	L292	5255.5
L265	L288	V293	4802.2
L265	L292	L225	5542.1
L265	L292	V241	5195.7
L265	L292	L288	6996.8
L265	V293	L288	5256.5
L265	L294	M239	4981.5
L265	L294	V241	4324.9
L268	I197	L225	8419.3
L268	I197	V241	8495.4
L268	I197	L265	5249.4
L268	I197	L288	9484.4
L268	I197	L292	9382.9
L268	I197	V293	10169.1
L268	L198	L265	6681.3
L268	L225	I197	2741
L268	L225	V241	4739.9
L268	L225	L288	4865
L268	L225	L292	5564.5
L268	V241	I197	3418.6
L268	V241	L225	5858.7
L268	V241	L292	6255.5
L268	L265	I197	6026.4
L268	L265	L198	8975.8

First Res	Second Res	Third Res	Scaled Flow
L268	L268	L66	272.3
L268	L268	I197	2913.6
L268	L268	L198	2366.9
L268	L268	L225	254.1
L268	L268	V241	495.1
L268	L268	V250	132.4
L268	L268	M262	1733.9
L268	L268	L265	3371.9
L268	L268	L288	89.9
L268	L268	L292	113.7
L268	L268	V293	83.1
L268	L288	I197	2354.1
L268	L288	L225	3709.1
L268	L288	L292	4859.2
L268	L288	V293	4428.3
L268	L292	I197	2464.2
L268	L292	L225	4488.8
L268	L292	V241	4082.6
L268	L292	L288	5141.5
L268	L292	V293	4282.4
L268	V293	I197	2477.3
L268	V293	L288	4346.4
L268	V293	L292	3972.4
I276	L225	I238	3940.8
I276	L225	V241	5551.5
I276	L225	L288	4672.9
I276	L225	L292	5294.2
I276	L225	L294	4770.8
I276	I238	L225	5664.7
I276	I238	V241	6073.4
I276	V241	L225	4634.9
I276	V241	I238	3527.5
I276	V241	L292	4563
I276	V241	L294	4511.1
I276	I276	V23	527.7
I276	I276	L66	70.7
I276	I276	I130	237
I276	I276	V134	220.8
I276	I276	L225	312.2
I276	I276	I238	863.5
I276	I276	V241	165.5

First Res	Second Res	Third Res	Scaled Flow
I276	I276	L277	1535.8
I276	I276	L288	191.4
I276	I276	L292	291.7
I276	I276	L294	142.7
I276	L277	L288	6646.2
I276	L288	L225	4056.9
I276	L288	L277	3050.8
I276	L288	L292	5156.6
I276	L292	L225	5185.9
I276	L292	V241	5353.6
I276	L292	L288	5818
I276	L294	L225	3832.1
I276	L294	V241	4340.1
L277	L225	V241	7639.1
L277	L225	L292	5584.3
L277	I238	V241	8404.3
L277	V241	L225	3763.1
L277	V241	I238	2976.7
L277	V241	L288	2467.3
L277	V241	L292	3678.2
L277	L277	V23	205.7
L277	L277	I130	1373.2
L277	L277	V134	113.7
L277	L277	M216	97.3
L277	L277	L225	813.3
L277	L277	I238	1623.2
L277	L277	M239	76.6
L277	L277	V241	76.6
L277	L277	I276	1535.8
L277	L277	L288	1623.2
L277	L277	L292	813.3
L277	L277	V293	312.2
L277	L277	L367	291.7
L277	L288	V241	6966.1
L277	L288	L292	7435
L277	L288	V293	7429
L277	L292	L225	5584.3
L277	L292	V241	7466.8
L277	L292	L288	5345.7
L277	L292	V293	5408.7
L277	L292	L367	5299.3

First Res	Second Res	Third Res	Scaled Flow
L277	V293	L288	3809.7
L277	V293	L292	3857.7
L277	V293	L367	4310.9
L277	L367	L292	3702.3
L277	L367	V293	4222.6
L283	L283	L154	916.4
L283	L283	I169	87.4
L283	L283	I209	165.5
L283	L283	V212	97.3
L283	L283	I220	3226.2
L283	L283	L288	381.7
L283	L283	M291	1452.5
L283	L283	V293	76.6
L283	L288	V293	5903.8
L283	V293	L288	3828
V285	L78	M81	5585.7
V285	L78	I144	5872.3
V285	L78	L337	5535.1
V285	M81	L78	4161.2
V285	M81	I144	4148.6
V285	I144	L78	3608.8
V285	I144	M81	3422.3
V285	V285	L78	527.7
V285	V285	M81	205.7
V285	V285	L90	133.9
V285	V285	V134	1623.2
V285	V285	L141	122.7
V285	V285	I144	97.3
V285	V285	L337	527.7
V285	L337	L78	5535.1
L287	I238	V241	4418.7
L287	V241	I238	4091.6
L287	V241	L288	3795.6
L287	V241	L292	4649
L287	L287	L159	76.6
L287	L287	I238	165.5
L287	L287	V241	122.7
L287	L287	V250	178.1
L287	L287	L253	83.1
L287	L287	L288	76.6
L287	L287	L292	132.4

First Res	Second Res	Third Res	Scaled Flow
L287	L287	V293	220.8
L287	L287	L367	76.6
L287	L288	V241	3390.9
L287	L288	L292	4683.7
L287	L288	V293	4012.7
L287	L288	L367	3402.1
L287	L292	V241	4738.5
L287	L292	L288	5343.6
L287	L292	V293	4028.6
L287	L292	L367	4326.6
L287	V293	L288	5248
L287	V293	L292	4618.1
L287	V293	L367	4692.8
L287	L367	L288	3402.1
L287	L367	L292	3792.3
L287	L367	V293	3588.2
L288	L154	M291	3080.1
L288	I197	L198	6481.9
L288	I197	V241	5136.2
L288	I197	L265	7286.2
L288	I197	L268	8106.4
L288	L198	I197	4374.8
L288	L198	L265	6441.6
L288	L198	L268	5812
L288	I220	L283	4190.3
L288	I220	M291	4102.6
L288	L225	V241	6541.7
L288	L225	L294	8020.3
L288	M239	L294	4635
L288	V241	I197	4735.1
L288	V241	L225	4733.1
L288	V241	L292	3105.4
L288	V241	L294	6891.4
L288	L265	I197	4937.5
L288	L265	L198	6467.7
L288	L265	L268	6295.4
L288	L268	I197	3450.3
L288	L268	L198	3665.3
L288	L268	L265	3954.1
L288	I276	L277	2964.9
L288	L277	I276	6659.3

First Res	Second Res	Third Res	Scaled Flow
L288	L283	I220	5773.6
L288	L288	L66	153.8
L288	L288	I130	272.3
L288	L288	L154	70.7
L288	L288	I197	1225.9
L288	L288	L198	464.3
L288	L288	I209	76.6
L288	L288	I220	122.7
L288	L288	L225	1912.2
L288	L288	I238	813.3
L288	L288	M239	153.8
L288	L288	V241	1030.8
L288	L288	L265	454.8
L288	L288	L268	89.9
L288	L288	I276	191.4
L288	L288	L277	1623.2
L288	L288	L283	381.7
L288	L288	L287	76.6
L288	L288	M291	334
L288	L288	L292	5271.1
L288	L288	V293	2913.6
L288	L288	L294	132.4
L288	L288	L367	863.5
L288	M291	L154	4639
L288	M291	I220	5418.5
L288	L292	V241	11156
L288	L292	L294	12395.6
L288	L294	L225	2944.6
L288	L294	M239	4460.2
L288	L294	V241	3497
L288	L294	L292	1750.9
M291	L288	V293	5481.3
M291	M291	L30	70.7
M291	M291	L154	1092.6
M291	M291	I209	312.2
M291	M291	V212	813.3
M291	M291	I215	94.6
M291	M291	I220	2767.5
M291	M291	V250	105.2
M291	M291	L283	1452.5
M291	M291	L288	334

First Res	Second Res	Third Res	Scaled Flow
M291	M291	V293	122.7
M291	M291	L357	133.9
M291	V293	L288	4150.1
L292	I197	L198	5771
L292	I197	L265	6538
L292	I197	L268	8201.9
L292	L198	I197	4772.5
L292	L198	L265	6873.9
L292	L198	L268	6782.8
L292	M239	L294	4359.3
L292	L265	I197	5572.8
L292	L265	L198	7085.1
L292	L265	L268	7551.6
L292	L268	I197	3489.8
L292	L268	L198	3489.8
L292	L268	L265	3769.6
L292	L292	L66	495.1
L292	L292	I197	1373.2
L292	L292	L198	916.4
L292	L292	L225	3940.5
L292	L292	I238	972.2
L292	L292	M239	407.7
L292	L292	V241	3393.2
L292	L292	L265	957.8
L292	L292	L268	113.7
L292	L292	I276	291.7
L292	L292	L277	813.3
L292	L292	L287	132.4
L292	L292	L288	5271.1
L292	L292	V293	2018
L292	L292	L294	863.5
L292	L292	L367	1811.2
L292	L294	M239	5757.9
V293	I197	L198	6124.5
V293	I197	V241	6672.5
V293	I197	L265	7357.5
V293	I197	L268	8735.5
V293	L198	I197	4564.1
V293	L198	M262	5562.4
V293	L198	L265	7053.2
V293	L198	L268	6681.8

First Res	Second Res	Third Res	Scaled Flow
V293	I220	L283	4420.8
V293	I220	M291	4154.9
V293	L225	I238	6744.1
V293	L225	V241	6492.7
V293	L225	L292	4709.9
V293	L225	L294	5399.5
V293	I238	L225	3446.6
V293	I238	V241	3735.3
V293	I238	L277	3654.9
V293	M239	L253	5288.5
V293	M239	L294	5609
V293	V241	I197	3955.8
V293	V241	L225	4671
V293	V241	I238	5258.3
V293	V241	L292	3954.4
V293	V241	L294	4831.2
V293	L253	M239	3591.8
V293	M262	L198	3014.5
V293	M262	L265	3598.4
V293	M262	L268	4086.2
V293	L265	I197	5007.6
V293	L265	L198	6441.6
V293	L265	M262	6064.2
V293	L265	L268	6774.1
V293	L268	I197	3348.4
V293	L268	L198	3436.8
V293	L268	M262	3878.3
V293	L268	L265	3815.1
V293	L277	I238	4727.6
V293	L283	I220	4420.8
V293	L283	M291	3645.6
V293	L288	L292	7151.3
V293	M291	I220	4650.8
V293	M291	L283	4080.7
V293	L292	L225	6910.4
V293	L292	V241	8064.6
V293	L292	L288	5495.4
V293	V293	I197	1452.5
V293	V293	L198	765.6
V293	V293	I209	464.3
V293	V293	I220	76.6

First Res	Second Res	Third Res	Scaled Flow
V293	V293	L225	972.2
V293	V293	I238	122.7
V293	V293	M239	677.6
V293	V293	V241	407.7
V293	V293	V250	153.8
V293	V293	L253	205.7
V293	V293	M262	102.3
V293	V293	L265	587.6
V293	V293	L268	83.1
V293	V293	L277	312.2
V293	V293	L283	76.6
V293	V293	L287	220.8
V293	V293	L288	2913.6
V293	V293	M291	122.7
V293	V293	L292	2018
V293	V293	L294	334
V293	V293	L367	1715
V293	L294	L225	3644.5
V293	L294	M239	4392.8
V293	L294	V241	4532.8
L294	I197	L198	6099.1
L294	I197	L265	6407.9
L294	L198	I197	4565
L294	L198	L265	6194.2
L294	L225	I238	6800.3
L294	L225	V241	4880.5
L294	L225	L288	7964
L294	L225	L292	6864.2
L294	I238	L225	3487.3
L294	I238	V241	3138.8
L294	V241	L225	5799.5
L294	V241	I238	7273.1
L294	V241	L292	7143.1
L294	L265	I197	5534
L294	L265	L198	7147.1
L294	L288	L225	3021.2
L294	L288	L292	4356.4
L294	L288	V293	4198.4
L294	L292	L225	4750.2
L294	L292	V241	4159.9
L294	L292	L288	7947.1

First Res	Second Res	Third Res	Scaled Flow
L294	L292	V293	5423
L294	L292	L367	5571.5
L294	V293	L288	5440.3
L294	V293	L292	3852.1
L294	V293	L367	4484.5
L294	L294	I197	1030.8
L294	L294	L198	495.1
L294	L294	L225	1811.2
L294	L294	I238	381.7
L294	L294	M239	2767.5
L294	L294	V241	2366.9
L294	L294	V250	178.1
L294	L294	L253	464.3
L294	L294	L265	708.2
L294	L294	I276	142.7
L294	L294	L288	132.4
L294	L294	L292	863.5
L294	L294	V293	334
L294	L294	L367	237
L294	L367	L292	3571.9
L294	L367	V293	4047.4
V300	V300	V45	132.4
V333	V186	V187	4967.3
V333	V187	V186	4644.2
V333	V333	V186	562.2
V333	V333	V187	464.3
L337	V26	L89	7417.6
L337	V26	I149	8615.7
L337	V26	L170	6560.3
L337	L77	M81	6205.8
L337	L77	V86	6459.6
L337	L77	L89	6786.6
L337	L77	L174	6079
L337	L78	M81	9788.1
L337	M81	L77	4357.2
L337	M81	L78	3009.8
L337	V86	L77	3498.2
L337	V86	I87	3815.5
L337	I87	V86	4725.9
L337	I87	L89	5001.2
L337	I87	M112	5400.2

First Res	Second Res	Third Res	Scaled Flow
L337	L89	V26	3110.5
L337	L89	L77	4451.4
L337	L89	I87	4890.4
L337	L89	M112	5608.2
L337	L89	L174	4926.5
L337	M112	I87	3649.3
L337	M112	L89	3875.7
L337	M112	L174	3466.4
L337	I149	V26	2403.1
L337	L170	V26	4615.3
L337	L174	L77	4967.8
L337	L174	L89	6138
L337	L174	M112	6249.5
L337	L337	V16	813.3
L337	L337	V26	2767.5
L337	L337	L75	122.7
L337	L337	L77	1349.8
L337	L337	L78	4139.3
L337	L337	M81	598.6
L337	L337	V86	272.3
L337	L337	I87	527.7
L337	L337	L89	495.1
L337	L337	M109	83.1
L337	L337	M112	142.7
L337	L337	L141	70.7
L337	L337	I144	863.5
L337	L337	I149	122.7
L337	L337	L167	97.3
L337	L337	I169	110.6
L337	L337	L170	1623.2
L337	L337	L174	873.1
L337	L337	V285	527.7
L357	I220	M291	4639.6
L357	M291	I220	4464.2
L357	L357	I209	982.9
L357	L357	I220	155.5
L357	L357	M291	133.9
V361	L125	M216	4932.7
V361	M216	L125	3694.6
V361	V361	M120	68.8
V361	V361	L125	239.6

First Res	Second Res	Third Res	Scaled Flow
V361	V361	M137	1468.6
V361	V361	L154	68.6
V361	V361	L159	68.6
V361	V361	L167	248.1
V361	V361	M216	77.5
L367	L225	V241	6243.1
L367	L225	L292	4440.6
L367	L225	L294	5040.4
L367	V241	L225	4457.7
L367	V241	L292	3700.2
L367	V241	L294	4400.5
L367	L288	L292	5280.2
L367	L288	V293	4285.2
L367	L292	L225	7585.9
L367	L292	V241	8852.7
L367	L292	L288	7630.1
L367	V293	L288	5997.5
L367	L294	L225	3820.2
L367	L294	V241	4671
L367	L367	L225	562.2
L367	L367	V241	191.4
L367	L367	L277	291.7
L367	L367	L287	76.6
L367	L367	L288	863.5
L367	L367	L292	1811.2
L367	L367	V293	1715
L367	L367	L294	237

Table S11. Community membership for +ATP inactive p38γ

module1	16, 26, 30, 33, 53, 58, 75, 77, 78, 81, 86, 87, 89, 109, 112, 116, 125, 141, 144, 149, 161, 167, 169, 170, 174, 216, 219, 285, 337
module2	23, 66, 120, 130, 134, 154, 197, 198, 209, 216, 220, 225, 238, 239, 241, 265, 268, 276, 277, 283, 287, 288, 291, 292, 293, 294, 367
module3	66, 150, 197, 198, 209, 225, 238, 239, 241, 250, 253, 262, 265, 268, 288, 292, 293, 294
module4	197, 198, 201, 225, 238, 239, 241, 250, 253, 262, 265, 277, 287, 288, 292, 293, 294
module5	30, 130, 154, 169, 209, 212, 215, 216, 220, 250, 262, 283, 288, 291, 293, 357, 361
module6	53, 77, 86, 87, 89, 112, 116, 161, 167, 169, 174, 215, 219, 283, 337
module7	116, 125, 130, 137, 154, 167, 209, 215, 216, 219, 277, 361
module8	120, 125, 130, 137, 154, 159, 167, 215, 216, 287, 361
module9	182, 186, 187, 333
module10	23, 78, 81, 90, 134, 141, 144, 276, 277, 285, 337
module11	41, 119
module12	23, 45, 300
module13	288, 294
module14	238, 293
module15	55
module16	66, 292
module17	250, 294
module18	323
module19	343

Table S12. Methyl 3-residue flow for activated p38γ

First Res	Second Res	Third Res	Scaled Flow
V16	V16	L283	59.9
V16	V16	V343	42.6
V23	V23	V41	97.3
V23	V23	I55	70.7
V23	V23	L119	728.4
V26	V26	V33	83.1
V26	V26	V45	46.5
V26	V26	L58	97.3
V26	V26	L77	220.8
V26	V26	L78	2494.4
V26	V26	V86	972.2
V26	V26	L89	191.4
V26	V26	L141	165.5
V26	V26	I169	70.7
V26	V26	L170	178.1
V26	V26	L287	97.3
V26	V26	V323	6979.5
V26	V33	V45	3931.0
V26	V33	L141	3594.1
V26	V45	V33	3454.1
V26	L141	V33	4264.5
V26	I169	L170	3407.1
V26	L170	I169	4278.3
L30	L30	V41	55.1
L30	L30	I55	5271.1
L30	L30	I87	637.0
L30	L30	L89	191.4
L30	L30	M109	272.3
L30	L30	M112	1092.6
L30	L30	L119	139.0
L30	L30	L167	495.1
L30	L30	I197	122.7
L30	I55	L119	11723.1
L30	L119	I55	1681.1
V33	V26	L78	4002.8
V33	V33	V26	83.1
V33	V33	V45	1373.2
V33	V33	L78	178.1
V33	V33	L141	1452.5
V33	V33	V343	122.7
V33	L78	V26	4842.9
V41	V23	L119	3579.0
V41	L30	I55	4400.4
V41	L30	M112	2888.5
V41	V41	V23	97.3
V41	V41	L30	55.1
V41	V41	I55	153.8
V41	V41	M81	55.1
V41	V41	M112	464.3
V41	V41	L119	41.3
V41	I55	L30	5617.7
V41	I55	M112	3996.8
V41	I55	L119	3935.7
V41	M112	L30	5119.5
V41	M112	I55	5548.7
V41	L119	V23	2962.0
V41	L119	I55	2905.6
V45	V26	L78	3988.5
V45	V26	V86	3187.2
V45	V33	L141	7461.5
V45	V45	V26	46.5
V45	V45	V33	1373.2
V45	V45	L78	50.6
V45	V45	V86	97.3
V45	V45	I87	97.3
V45	V45	L141	59.9
V45	V45	L287	153.8
V45	V45	V300	59.9
V45	V45	V343	381.7
V45	L78	V26	4062.5
V45	L78	V86	3263.3
V45	L78	L141	3383.3
V45	V86	V26	3765.7
V45	V86	L78	3785.4
V45	L141	V33	2734.4
V45	L141	L78	3510.2
V45	L287	V343	3683.8
V45	V343	L287	4791.9

First Res	Second Res	Third Res	Scaled Flow
V53	V53	L116	237.0
V53	V53	V134	49.1
V53	V53	V161	76.6
V53	V53	L167	407.7
V53	V53	I169	65.1
V53	V53	V212	65.1
V53	V53	M219	291.7
V53	V53	L287	178.1
V53	L116	M219	4061.7
V53	M219	L116	4317.2
I55	I55	V23	70.7
I55	I55	L30	5271.1
I55	I55	V41	153.8
I55	I55	M81	142.7
I55	I55	I87	1715.0
I55	I55	L89	237.0
I55	I55	M109	272.3
I55	I55	M112	2494.4
I55	I55	L119	797.9
I55	I55	L167	435.2
I55	I55	I197	70.7
I55	M81	M109	3939.5
I55	M109	M81	4706.7
L58	V26	L78	4277.3
L58	V26	V323	5270.4
L58	L58	V26	97.3
L58	L58	L75	464.3
L58	L58	L77	407.7
L58	L58	L78	105.2
L58	L58	M81	165.5
L58	L58	M109	142.7
L58	L58	I149	89.9
L58	L58	L174	272.3
L58	L58	M182	105.2
L58	L58	V250	220.8
L58	L58	L253	83.1
L58	L58	V323	89.9
L58	L75	M182	5018.1
L58	L77	M81	5346.5
L58	L77	L174	4363.8
L58	L78	V26	4358.6

First Res	Second Res	Third Res	Scaled Flow
L58	L78	V323	3744.0
L58	M81	L77	4101.5
L58	M81	M109	4305.9
L58	M109	M81	4142.6
L58	L174	L77	3847.9
L58	M182	L75	3285.8
L58	V250	L253	5213.5
L58	L253	V250	4061.2
L58	V323	V26	5172.5
L58	V323	L78	3605.9
L66	L66	L89	165.5
L66	L66	I144	178.1
L66	L66	L159	161.3
L66	L66	I169	2366.9
L66	L66	L170	1373.2
L75	L75	L58	464.3
L75	L75	M182	1373.2
L75	L75	M201	191.4
L75	L75	V250	191.4
L75	L75	L253	105.2
L75	L75	V333	89.9
L75	L75	L337	334.0
L75	M182	M201	7384.4
L75	M201	M182	3596.1
L75	V250	L253	4971.9
L75	L253	V250	4262.8
L77	V26	L78	4357.6
L77	V26	V86	3999.8
L77	V26	V323	5833.6
L77	L77	V26	220.8
L77	L77	L58	407.7
L77	L77	L78	357.2
L77	L77	M81	2494.4
L77	L77	V86	105.2
L77	L77	I87	291.7
L77	L77	M109	637.0
L77	L77	I144	1092.6
L77	L77	L170	153.8
L77	L77	L174	1452.5
L77	L77	M182	435.2
L77	L77	M201	55.1

First Res	Second Res	Third Res	Scaled Flow
L77	L77	V285	59.9
L77	L77	V323	191.4
L77	L78	V26	5030.6
L77	L78	V86	4427.8
L77	L78	V323	4217.9
L77	V86	V26	3296.1
L77	V86	L78	3160.6
L77	M182	M201	6137.8
L77	M201	M182	3539.9
L77	V323	V26	5607.0
L77	V323	L78	3511.6
L78	V26	V323	9000.2
L78	V33	V45	4457.1
L78	V33	L141	3149.6
L78	V45	V33	3296.9
L78	L77	M81	4608.1
L78	L77	L174	4136.2
L78	L78	V26	2494.4
L78	L78	V33	178.1
L78	L78	V45	50.6
L78	L78	L58	105.2
L78	L78	L77	357.2
L78	L78	M81	435.2
L78	L78	V86	1030.8
L78	L78	I87	165.5
L78	L78	L89	598.6
L78	L78	M109	334.0
L78	L78	L141	1030.8
L78	L78	I149	165.5
L78	L78	I169	165.5
L78	L78	L170	562.2
L78	L78	L174	334.0
L78	L78	V285	495.1
L78	L78	L287	97.3
L78	L78	V323	1157.6
L78	M81	L77	4914.6
L78	M81	M109	4619.0
L78	M109	M81	4240.7
L78	L141	V33	5745.1
L78	I169	L170	3332.1
L78	L170	I169	4852.8

First Res	Second Res	Third Res	Scaled Flow
L78	L174	L77	4050.0
L78	V323	V26	5755.0
M81	I55	I87	3782.7
M81	I55	M112	4418.5
M81	M81	V41	55.1
M81	M81	I55	142.7
M81	M81	L58	165.5
M81	M81	L77	2494.4
M81	M81	L78	435.2
M81	M81	I87	312.2
M81	M81	M109	1912.2
M81	M81	M112	153.8
M81	M81	I144	813.3
M81	M81	I169	89.9
M81	M81	L170	435.2
M81	M81	L174	972.2
M81	M81	V285	132.4
M81	I87	I55	4709.3
M81	M112	I55	4504.5
M81	I169	L170	3205.0
M81	L170	I169	4972.1
V86	V26	L78	4706.9
V86	V26	V323	6937.2
V86	L78	V26	4831.6
V86	V86	V26	972.2
V86	V86	V45	97.3
V86	V86	L77	105.2
V86	V86	L78	1030.8
V86	V86	L141	237.0
V86	V86	I149	105.2
V86	V86	V241	50.6
V86	V86	V323	813.3
V86	V323	V26	6429.4
I87	L30	I55	5049.2
I87	I55	L30	7977.1
I87	I55	M112	6916.0
I87	L77	M81	4644.1
I87	M81	L77	4741.1
I87	M81	M109	4314.2
I87	I87	L30	637.0
I87	I87	V45	97.3

First Res	Second Res	Third Res	Scaled Flow
I87	I87	I55	1715.0
I87	I87	L77	291.7
I87	I87	L78	165.5
I87	I87	M81	312.2
I87	I87	M109	357.2
I87	I87	M112	381.7
I87	I87	L119	215.5
I87	I87	L283	65.1
I87	I87	V285	178.1
I87	M109	M81	4498.9
I87	M112	I55	3661.8
L89	V26	L78	3998.3
L89	L30	I55	5254.3
L89	L30	M112	4271.8
L89	I55	L30	5577.2
L89	I55	M112	5295.3
L89	L66	I169	4449.7
L89	L66	L170	3782.9
L89	L78	V26	5729.7
L89	L89	V26	191.4
L89	L89	L30	191.4
L89	L89	I55	237.0
L89	L89	L66	165.5
L89	L89	L78	598.6
L89	L89	M109	312.2
L89	L89	M112	55.1
L89	L89	L159	173.6
L89	L89	L167	42.6
L89	L89	I169	165.5
L89	L89	L170	237.0
L89	L89	I215	178.1
L89	M112	L30	3156.0
L89	M112	I55	3685.6
L89	I169	L66	4449.7
L89	I169	L170	3731.6
L89	L170	L66	4175.6
L89	L170	I169	4119.0
L90	L90	L174	74.4
L90	L90	M201	119.4
M109	L30	I55	5547.9
M109	L30	M112	4680.7

First Res	Second Res	Third Res	Scaled Flow
M109	I55	L30	5547.9
M109	I55	I87	4100.0
M109	I55	M112	5565.4
M109	L77	M81	3764.3
M109	M81	L77	6344.6
M109	I87	I55	4455.2
M109	M109	L30	272.3
M109	M109	I55	272.3
M109	M109	L58	142.7
M109	M109	L77	637.0
M109	M109	L78	334.0
M109	M109	M81	1912.2
M109	M109	I87	357.2
M109	M109	L89	312.2
M109	M109	M112	42.6
M109	M109	I144	407.7
M109	M109	L167	65.1
M109	M109	I169	89.9
M109	M109	L170	122.7
M109	M109	L174	916.4
M109	M109	M182	50.6
M109	M109	L337	464.3
M109	M112	L30	2960.8
M109	M112	I55	3520.4
M109	I169	L170	3645.2
M109	L170	I169	3930.8
M112	L30	I55	5095.5
M112	I55	L30	8187.7
M112	I55	I87	6973.6
M112	M81	M109	4700.8
M112	I87	I55	2898.8
M112	M109	M81	3484.8
M112	M112	L30	1092.6
M112	M112	V41	464.3
M112	M112	I55	2494.4
M112	M112	M81	153.8
M112	M112	I87	381.7
M112	M112	L89	55.1
M112	M112	M109	42.6
M112	M112	L119	139.0
M112	M112	V161	178.1

First Res	Second Res	Third Res	Scaled Flow
L116	L116	V53	237.0
L116	L116	L125	4346.9
L116	L116	M137	46.5
L116	L116	L159	315.7
L116	L116	M216	97.3
L116	L116	M219	1623.2
L116	L116	L367	76.6
L119	L30	I55	4425.2
L119	L30	M112	3702.3
L119	I55	L30	7716.1
L119	I55	I87	5460.7
L119	I55	M112	6433.6
L119	I87	I55	3521.5
L119	M112	L30	3702.3
L119	M112	I55	3689.7
L119	L119	V23	728.4
L119	L119	L30	139.0
L119	L119	V41	41.3
L119	L119	I55	797.9
L119	L119	I87	215.5
L119	L119	M112	139.0
L119	L119	I197	133.9
L119	L119	L265	41.3
M120	M120	L225	220.8
M120	M120	V241	42.6
M120	M120	L265	55.1
M120	M120	L277	598.6
M120	M120	L292	70.7
M120	L225	V241	4601.0
M120	L225	L292	4505.2
M120	V241	L225	3092.0
M120	V241	L265	3729.9
M120	V241	L292	3710.6
M120	L265	V241	3941.2
M120	L292	L225	3381.4
M120	L292	V241	4144.2
L125	L116	M219	9708.4
L125	L125	L116	4346.9
L125	L125	M137	46.5
L125	L125	L159	361.1
L125	L125	I169	59.9

First Res	Second Res	Third Res	Scaled Flow
L125	L125	M219	357.2
L125	L125	L367	65.1
L125	M137	I169	3645.7
L125	I169	M137	3852.6
L125	M219	L116	2362.8
I130	I130	I215	110.6
I130	I130	M216	41.3
I130	I130	I220	1275.3
I130	I130	I238	149.8
I130	I130	L277	440.0
I130	I130	V285	173.6
I130	I238	L277	4006.3
I130	L277	I238	5498.6
V134	V134	V53	49.1
M137	L116	L125	4425.9
M137	L125	L116	4425.9
M137	M137	L116	46.5
M137	M137	L125	46.5
M137	M137	L141	42.6
M137	M137	I169	1623.2
M137	M137	L170	97.3
M137	M137	I209	49.1
M137	M137	L283	89.9
M137	M137	L287	357.2
M137	I169	L170	7159.1
M137	L170	I169	2681.8
L141	V26	L78	3640.5
L141	V26	V323	5701.4
L141	V33	V45	7510.6
L141	V45	V33	2673.1
L141	L78	V26	6771.3
L141	L78	V323	5722.6
L141	M137	I169	3429.4
L141	L141	V26	165.5
L141	L141	V33	1452.5
L141	L141	V45	59.9
L141	L141	L78	1030.8
L141	L141	V86	237.0
L141	L141	M137	42.6
L141	L141	I149	76.6
L141	L141	I169	122.7

First Res	Second Res	Third Res	Scaled Flow
L141	L141	L170	254.1
L141	L141	L174	191.4
L141	L141	L287	76.6
L141	L141	V323	122.7
L141	L141	V343	76.6
L141	I169	M137	4367.6
L141	I169	L170	3547.1
L141	L170	I169	4314.4
L141	L287	V343	3869.0
L141	V323	V26	5279.5
L141	V323	L78	2849.0
L141	V343	L287	3869.0
I144	L66	I169	4632.4
I144	L66	L170	3951.1
I144	L77	M81	5111.7
I144	L77	L174	5469.2
I144	M81	L77	4494.0
I144	M81	M109	5070.6
I144	M109	M81	3936.0
I144	I144	L66	178.1
I144	I144	L77	1092.6
I144	I144	M81	813.3
I144	I144	M109	407.7
I144	I144	I169	122.7
I144	I144	L170	178.1
I144	I144	L174	254.1
I144	I144	M182	142.7
I144	I144	V361	256.9
I144	I169	L66	4206.8
I144	I169	L170	3684.1
I144	L170	L66	3951.1
I144	L170	I169	4056.9
I144	L174	L77	3224.9
I149	L78	V86	3864.4
I149	L78	L141	3987.3
I149	V86	L78	3445.4
I149	L141	L78	3298.5
I149	I149	L58	89.9
I149	I149	L78	165.5
I149	I149	V86	105.2
I149	I149	L141	76.6

First Res	Second Res	Third Res	Scaled Flow
I149	I149	L337	70.7
I149	I149	L367	334.0
I150	I150	L198	357.2
I150	I150	M239	2245.1
I150	I150	V241	46.5
I150	I150	V250	165.5
I150	I150	L253	863.5
I150	I150	M262	254.1
I150	I150	L268	105.2
I150	I150	M291	132.4
I150	I150	L292	50.6
I150	L198	M262	6035.6
I150	M239	V241	9484.6
I150	V241	M239	2463.8
I150	V241	L292	3836.5
I150	V250	L253	3886.7
I150	L253	V250	6691.8
I150	M262	L198	5442.6
I150	L292	V241	3907.7
L154	L154	V361	215.5
L159	L66	I169	4181.9
L159	L66	L170	4388.2
L159	L116	L125	5302.3
L159	L116	M219	4230.8
L159	L125	L116	5529.9
L159	L159	L66	161.3
L159	L159	L89	173.6
L159	L159	L116	315.7
L159	L159	L125	361.1
L159	L159	L167	80.7
L159	L159	I169	294.9
L159	L159	L170	49.1
L159	L159	I215	128.9
L159	L159	M216	167.3
L159	L159	M219	305.1
L159	L159	L287	49.1
L159	I169	L66	4956.4
L159	I169	L170	4876.0
L159	L170	L66	3309.8
L159	L170	I169	3103.1
L159	M219	L116	4187.1

First Res	Second Res	Third Res	Scaled Flow
V161	V161	V53	76.6
V161	V161	M112	178.1
V161	V161	L167	65.1
V161	V161	V212	132.4
V161	V161	L283	272.3
L167	L30	I55	5966.2
L167	I55	L30	5708.9
L167	L167	L30	495.1
L167	L167	V53	407.7
L167	L167	I55	435.2
L167	L167	L89	42.6
L167	L167	M109	65.1
L167	L167	L159	80.7
L167	L167	V161	65.1
L167	L167	I169	435.2
L167	L167	L287	527.7
L167	L167	V343	142.7
L167	L167	V361	80.7
L167	L287	V343	5181.1
L167	V343	L287	3501.2
I169	V26	L78	3929.5
I169	V26	V323	4669.9
I169	L78	V26	4839.0
I169	L78	L141	3802.9
I169	L78	V323	3598.3
I169	M81	M109	4051.4
I169	M81	I144	3292.7
I169	M109	M81	4051.4
I169	L141	L78	3521.5
I169	I144	M81	3550.7
I169	I169	V26	70.7
I169	I169	V53	65.1
I169	I169	L66	2366.9
I169	I169	L78	165.5
I169	I169	M81	89.9
I169	I169	L89	165.5
I169	I169	M109	89.9
I169	I169	L125	59.9
I169	I169	M137	1623.2
I169	I169	L141	122.7
I169	I169	I144	122.7

First Res	Second Res	Third Res	Scaled Flow
I169	I169	L159	294.9
I169	I169	L167	435.2
I169	I169	L170	1297.7
I169	I169	L287	165.5
I169	I169	V323	254.1
I169	I169	V343	46.5
I169	L287	V343	4582.2
I169	V323	V26	6477.1
I169	V323	L78	4052.7
I169	V343	L287	3393.3
L170	V26	L78	3989.1
L170	V26	V323	5720.3
L170	L77	M81	4026.6
L170	L77	I144	3660.0
L170	L77	L174	3374.1
L170	L78	V26	5697.4
L170	L78	V323	4807.6
L170	M81	L77	5468.9
L170	M81	M109	5265.8
L170	M109	M81	3660.4
L170	M137	I169	2962.0
L170	I144	L77	3805.1
L170	I169	M137	7024.8
L170	L170	V26	178.1
L170	L170	L66	1373.2
L170	L170	L77	153.8
L170	L170	L78	562.2
L170	L170	M81	435.2
L170	L170	L89	237.0
L170	L170	M109	122.7
L170	L170	M137	97.3
L170	L170	L141	254.1
L170	L170	I144	178.1
L170	L170	L159	49.1
L170	L170	I169	1297.7
L170	L170	L174	598.6
L170	L170	V323	142.7
L170	L174	L77	5126.4
L170	V323	V26	5397.1
L170	V323	L78	3175.9
L174	L77	M81	5205.9

First Res	Second Res	Third Res	Scaled Flow
L174	M81	L77	4290.0
L174	L174	L58	272.3
L174	L174	L77	1452.5
L174	L174	L78	334.0
L174	L174	M81	972.2
L174	L174	L90	74.4
L174	L174	M109	916.4
L174	L174	L141	191.4
L174	L174	I144	254.1
L174	L174	L170	598.6
L174	L174	M182	132.4
L174	L174	M201	312.2
L174	M182	M201	4129.7
L174	M201	M182	5240.8
M182	L77	I144	4489.8
M182	L77	L174	4877.8
M182	M109	L174	3106.0
M182	I144	L77	3242.7
M182	L174	L77	3456.0
M182	L174	M109	3886.5
M182	M182	L58	105.2
M182	M182	L75	1373.2
M182	M182	L77	435.2
M182	M182	M109	50.6
M182	M182	I144	142.7
M182	M182	L174	132.4
M182	M182	V187	142.7
M182	M182	M201	2627.8
M182	M182	V333	55.1
M182	M182	L337	165.5
V186	V186	M201	105.2
V186	V186	M219	105.2
V187	M182	M201	4758.1
V187	V187	M182	142.7
V187	V187	L198	464.3
V187	V187	M201	70.7
V187	V187	M262	562.2
V187	L198	M262	5832.0
V187	M201	M182	4016.0
V187	M262	L198	6237.7
I197	L30	I55	5295.9

First Res	Second Res	Third Res	Scaled Flow
I197	I55	L30	4644.2
I197	I55	L119	3142.6
I197	L119	I55	3672.9
I197	I197	L30	122.7
I197	I197	I55	70.7
I197	I197	L119	133.9
I197	I197	M239	65.1
I197	I197	V250	50.6
I197	I197	L265	142.7
I197	I197	L288	42.6
I197	I197	V293	312.2
L198	I150	M239	5747.6
L198	L198	I150	357.2
L198	L198	V187	464.3
L198	L198	M239	46.5
L198	L198	V241	89.9
L198	L198	V250	122.7
L198	L198	L253	562.2
L198	L198	M262	5794.6
L198	L198	L294	50.6
L198	M239	I150	3407.7
L198	M239	V241	3434.6
L198	V241	M239	3982.6
L198	V241	L294	4046.3
L198	V250	L253	3930.8
L198	L253	V250	6182.3
L198	L294	V241	3554.2
M201	L75	M182	2565.3
M201	L77	L174	3209.1
M201	L174	L77	5003.1
M201	M182	L75	7869.7
M201	M201	L75	191.4
M201	M201	L77	55.1
M201	M201	L90	119.4
M201	M201	L174	312.2
M201	M201	M182	2627.8
M201	M201	V186	105.2
M201	M201	V187	70.7
M201	M201	V333	42.6
I209	I209	M137	49.1
V212	V212	V53	65.1

First Res	Second Res	Third Res	Scaled Flow
V212	V212	V161	132.4
V212	V212	I215	165.5
V212	V212	L277	272.3
V212	V212	L283	55.1
V212	V212	L287	122.7
I215	I215	L89	178.1
I215	I215	I130	110.6
I215	I215	L159	128.9
I215	I215	V212	165.5
I215	I215	M219	122.7
I215	I215	L277	42.6
M216	L116	M219	3716.9
M216	M216	L116	97.3
M216	M216	I130	41.3
M216	M216	L159	167.3
M216	M216	M219	191.4
M216	M216	M291	97.3
M216	M219	L116	4417.5
M219	L116	L125	8196.8
M219	L125	L116	4383.4
M219	M219	V53	291.7
M219	M219	L116	1623.2
M219	M219	L125	357.2
M219	M219	L159	305.1
M219	M219	V186	105.2
M219	M219	I215	122.7
M219	M219	M216	191.4
M219	M219	V300	65.1
I220	I220	I130	1275.3
I220	I220	I238	435.2
I220	I220	L277	312.2
I220	I238	L277	5007.0
I220	L277	I238	4502.0
L225	L225	M120	220.8
L225	L225	I238	1092.6
L225	L225	M239	89.9
L225	L225	V241	1225.9
L225	L225	L265	562.2
L225	L225	L277	637.0
L225	L225	L288	677.6
L225	L225	L292	1373.2

First Res	Second Res	Third Res	Scaled Flow
L225	L225	V293	637.0
L225	L225	L294	2366.9
L225	I238	L277	5408.9
L225	M239	V241	2833.4
L225	V241	M239	6657.1
L225	V241	L265	5207.8
L225	L265	V241	3759.4
L225	L265	L294	3432.6
L225	L277	I238	4319.1
L225	L288	L292	4452.8
L225	L292	L288	6088.8
L225	L294	L265	6968.0
I238	I130	I220	3400.8
I238	I220	I130	4637.6
I238	L225	L292	5594.2
I238	L225	L294	5459.6
I238	I238	I130	149.8
I238	I238	I220	435.2
I238	I238	L225	1092.6
I238	I238	M239	122.7
I238	I238	V241	191.4
I238	I238	L265	237.0
I238	I238	L268	407.7
I238	I238	L277	2494.4
I238	I238	L288	220.8
I238	I238	M291	220.8
I238	I238	L292	205.7
I238	I238	V293	254.1
I238	I238	L294	562.2
I238	M239	V241	3704.2
I238	V241	M239	4159.8
I238	V241	L265	4187.1
I238	V241	L288	4166.3
I238	V241	L292	4305.6
I238	V241	L294	3550.6
I238	L265	V241	4444.5
I238	L265	L294	4103.4
I238	L288	V241	4334.7
I238	L288	L292	4855.4
I238	L292	L225	3106.2
I238	L292	V241	4391.4

First Res	Second Res	Third Res	Scaled Flow
I238	L292	L288	4759.8
I238	L294	L225	4162.6
I238	L294	V241	4972.7
I238	L294	L265	5414.1
M239	L198	M262	4537.4
M239	L225	I238	3513.0
M239	L225	V241	2696.4
M239	L225	L292	3126.8
M239	L225	L294	3616.4
M239	I238	L225	3788.3
M239	M239	I150	2245.1
M239	M239	I197	65.1
M239	M239	L198	46.5
M239	M239	L225	89.9
M239	M239	I238	122.7
M239	M239	V241	1373.2
M239	M239	V250	132.4
M239	M239	L253	237.0
M239	M239	M262	76.6
M239	M239	L265	720.4
M239	M239	L268	765.6
M239	M239	L288	153.8
M239	M239	M291	55.1
M239	M239	L292	598.6
M239	M239	V293	142.7
M239	M239	L294	527.7
M239	V241	L225	6705.3
M239	V241	L288	6873.9
M239	V241	L292	5383.3
M239	V250	L253	4339.4
M239	L253	V250	5075.0
M239	M262	L198	5068.7
M239	L265	L294	5031.2
M239	L288	V241	3157.3
M239	L288	L292	4078.6
M239	L292	L225	5426.4
M239	L292	V241	3756.8
M239	L292	L288	6196.9
M239	L294	L225	5995.9
M239	L294	L265	4478.6
V241	I150	M239	2980.5

First Res	Second Res	Third Res	Scaled Flow
V241	L198	M262	4947.4
V241	I238	L277	4649.2
V241	M239	I150	8594.5
V241	V241	V86	50.6
V241	V241	M120	42.6
V241	V241	I150	46.5
V241	V241	L198	89.9
V241	V241	L225	1225.9
V241	V241	I238	191.4
V241	V241	M239	1373.2
V241	V241	M262	113.7
V241	V241	L265	1912.2
V241	V241	L277	153.8
V241	V241	L288	1811.2
V241	V241	L292	2018.0
V241	V241	V293	113.7
V241	V241	L294	1535.8
V241	M262	L198	5234.8
V241	L277	I238	4385.0
V250	I150	M239	4490.6
V250	L198	M262	5199.1
V250	M239	I150	4238.3
V250	V250	L58	220.8
V250	V250	L75	191.4
V250	V250	I150	165.5
V250	V250	I197	50.6
V250	V250	L198	122.7
V250	V250	M239	132.4
V250	V250	L253	2913.6
V250	V250	M262	122.7
V250	M262	L198	5199.1
L253	I150	M239	5924.9
L253	L198	M262	5924.9
L253	M239	I150	3798.4
L253	L253	L58	83.1
L253	L253	L75	105.2
L253	L253	I150	863.5
L253	L253	L198	562.2
L253	L253	M239	237.0
L253	L253	V250	2913.6
L253	L253	M262	677.6

First Res	Second Res	Third Res	Scaled Flow
L253	M262	L198	6353.0
M262	I150	M239	5111.4
M262	M239	I150	3754.3
M262	M239	V241	3626.2
M262	V241	M239	3982.7
M262	V241	L294	3756.2
M262	V250	L253	3848.6
M262	L253	V250	6490.4
M262	M262	I150	254.1
M262	M262	V187	562.2
M262	M262	L198	5794.6
M262	M262	M239	76.6
M262	M262	V241	113.7
M262	M262	V250	122.7
M262	M262	L253	677.6
M262	M262	L294	191.4
M262	L294	V241	4299.1
L265	L225	L292	5188.2
L265	L225	L294	3432.6
L265	I238	L277	5047.0
L265	V241	L288	7054.9
L265	V241	L292	8498.6
L265	L265	L119	41.3
L265	L265	M120	55.1
L265	L265	I197	142.7
L265	L265	L225	562.2
L265	L265	I238	237.0
L265	L265	M239	720.4
L265	L265	V241	1912.2
L265	L265	L268	113.7
L265	L265	L277	97.3
L265	L265	L288	254.1
L265	L265	L292	113.7
L265	L265	V293	59.9
L265	L265	L294	2366.9
L265	L277	I238	4000.5
L265	L288	V241	3090.9
L265	L288	L292	5275.6
L265	L292	L225	3236.8
L265	L292	V241	3003.7
L265	L292	L288	4255.7

First Res	Second Res	Third Res	Scaled Flow
L265	L294	L225	6968.0
L268	I150	M239	3490.4
L268	M239	I150	6414.8
L268	L265	L294	4182.5
L268	L268	I150	105.2
L268	L268	I238	407.7
L268	L268	M239	765.6
L268	L268	L265	113.7
L268	L268	L294	165.5
L268	L294	L265	4603.3
I276	I276	L292	215.5
L277	L225	V241	4965.8
L277	L225	L294	6293.2
L277	V241	L225	3193.2
L277	V241	L265	4402.2
L277	V241	L288	4142.8
L277	V241	L292	3944.8
L277	V241	L294	4230.7
L277	L265	V241	3927.0
L277	L265	L294	4282.7
L277	L277	M120	598.6
L277	L277	I130	440.0
L277	L277	V212	272.3
L277	L277	I215	42.6
L277	L277	I220	312.2
L277	L277	L225	637.0
L277	L277	I238	2494.4
L277	L277	V241	153.8
L277	L277	L265	97.3
L277	L277	L288	165.5
L277	L277	L292	334.0
L277	L277	L294	89.9
L277	L288	V241	4223.9
L277	L288	L292	4398.9
L277	L292	V241	4918.9
L277	L292	L288	5379.9
L277	L294	L225	3543.0
L277	L294	V241	3704.0
L277	L294	L265	4203.2
L283	L283	V16	59.9
L283	L283	I87	65.1

First Res	Second Res	Third Res	Scaled Flow
L283	L283	M137	89.9
L283	L283	V161	272.3
L283	L283	V212	55.1
L283	L283	L287	562.2
L283	L283	M291	407.7
L283	L283	V343	334.0
L283	L287	V343	4630.3
L283	V343	L287	3888.3
V285	L77	M81	3898.5
V285	M81	L77	4701.8
V285	V285	L77	59.9
V285	V285	L78	495.1
V285	V285	M81	132.4
V285	V285	I87	178.1
V285	V285	I130	173.6
L287	V26	L78	4302.5
L287	V26	V323	4968.5
L287	L78	V26	4302.5
L287	L78	L141	3659.6
L287	L78	V323	3397.2
L287	M137	I169	4686.9
L287	L141	L78	3460.2
L287	I169	M137	3752.5
L287	L287	V26	97.3
L287	L287	V45	153.8
L287	L287	V53	178.1
L287	L287	L78	97.3
L287	L287	M137	357.2
L287	L287	L141	76.6
L287	L287	L159	49.1
L287	L287	L167	527.7
L287	L287	I169	165.5
L287	L287	V212	122.7
L287	L287	L283	562.2
L287	L287	V323	220.8
L287	L287	V343	1623.2
L287	V323	V26	6143.8
L287	V323	L78	4200.8
L288	L225	L294	5196.9
L288	I238	L277	4736.7
L288	M239	V241	2756.3

First Res	Second Res	Third Res	Scaled Flow
L288	V241	M239	6991.8
L288	V241	L265	7036.0
L288	L265	V241	3185.2
L288	L265	L294	4329.3
L288	L277	I238	4378.1
L288	L288	I197	42.6
L288	L288	L225	677.6
L288	L288	I238	220.8
L288	L288	M239	153.8
L288	L288	V241	1811.2
L288	L288	L265	254.1
L288	L288	L277	165.5
L288	L288	L292	3066.4
L288	L288	V293	464.3
L288	L288	L294	407.7
L288	L294	L225	4338.0
L288	L294	L265	5010.6
M291	I150	M239	4633.2
M291	M239	I150	3771.4
M291	M291	I150	132.4
M291	M291	M216	97.3
M291	M291	I238	220.8
M291	M291	M239	55.1
M291	M291	L283	407.7
M291	M291	V293	55.1
L292	I150	M239	3285.4
L292	L225	L294	6481.4
L292	I238	L277	4341.2
L292	M239	I150	6491.0
L292	V241	L265	8574.3
L292	L265	V241	2930.4
L292	L265	L294	3916.1
L292	L277	I238	5005.8
L292	L292	M120	70.7
L292	L292	I150	50.6
L292	L292	L225	1373.2
L292	L292	I238	205.7
L292	L292	M239	598.6
L292	L292	V241	2018.0
L292	L292	L265	113.7
L292	L292	I276	215.5

First Res	Second Res	Third Res	Scaled Flow
L292	L292	L277	334.0
L292	L292	L288	3066.4
L292	L292	V293	237.0
L292	L292	L294	334.0
L292	L294	L225	3712.1
L292	L294	L265	5271.3
V293	L225	V241	5187.7
V293	L225	L292	4789.5
V293	L225	L294	4684.6
V293	M239	V241	3991.6
V293	V241	L225	3090.2
V293	V241	M239	3769.6
V293	V241	L265	4342.3
V293	V241	L288	3549.8
V293	V241	L292	3909.4
V293	V241	L294	3250.1
V293	L265	V241	3740.1
V293	L265	L294	3350.0
V293	L288	V241	5319.9
V293	L288	L292	5542.5
V293	L292	L225	3466.1
V293	L292	V241	4749.5
V293	L292	L288	4493.0
V293	V293	I197	312.2
V293	V293	L225	637.0
V293	V293	I238	254.1
V293	V293	M239	142.7
V293	V293	V241	113.7
V293	V293	L265	59.9
V293	V293	L288	464.3
V293	V293	M291	55.1
V293	V293	L292	237.0
V293	V293	L294	677.6
V293	L294	L225	4796.2
V293	L294	V241	5586.1
V293	L294	L265	6685.0
L294	L198	M262	4360.6
L294	I238	L277	6166.7
L294	V241	L288	5867.0
L294	V241	L292	6375.4
L294	M262	L198	6011.8

First Res	Second Res	Third Res	Scaled Flow
L294	L277	I238	3636.0
L294	L288	V241	3375.9
L294	L288	L292	5165.7
L294	L292	V241	3441.8
L294	L292	L288	4846.5
L294	L294	L198	50.6
L294	L294	L225	2366.9
L294	L294	I238	562.2
L294	L294	M239	527.7
L294	L294	V241	1535.8
L294	L294	M262	191.4
L294	L294	L265	2366.9
L294	L294	L268	165.5
L294	L294	L277	89.9
L294	L294	L288	407.7
L294	L294	L292	334.0
L294	L294	V293	677.6
V300	V300	V45	59.9
V300	V300	M219	65.1
V300	V300	V343	97.3
V323	I169	L170	4241.4
V323	L170	I169	3622.9
V323	V323	V26	6979.5
V323	V323	L58	89.9
V323	V323	L77	191.4
V323	V323	L78	1157.6
V323	V323	V86	813.3
V323	V323	L141	122.7
V323	V323	I169	254.1
V323	V323	L170	142.7
V323	V323	L287	220.8
V333	L75	M182	3933.7
V333	M182	L75	3519.5
V333	M182	M201	4177.7
V333	M201	M182	3953.8
V333	V333	L75	89.9
V333	V333	M182	55.1
V333	V333	M201	42.6
L337	L75	M182	4438.6
L337	M182	L75	3629.3
L337	L337	L75	334.0

First Res	Second Res	Third Res	Scaled Flow
L337	L337	M109	464.3
L337	L337	I149	70.7
L337	L337	M182	165.5
V343	V33	V45	3430.1
V343	V33	L141	4080.7
V343	V45	V33	4726.1
V343	L141	V33	3645.6
V343	V343	V16	42.6
V343	V343	V33	122.7
V343	V343	V45	381.7
V343	V343	L141	76.6
V343	V343	L167	142.7
V343	V343	I169	46.5
V343	V343	L283	334.0
V343	V343	L287	1623.2
V343	V343	V300	97.3
V361	V361	I144	256.9
V361	V361	L154	215.5
V361	V361	L167	80.7
L367	L116	L125	4721.7
L367	L125	L116	4549.8
L367	L367	L116	76.6
L367	L367	L125	65.1
L367	L367	I149	334.0

Table S13. Community membership for activated p38γ

module1	86, 119, 120, 150, 197, 198, 225, 238, 239, 241, 250, 253, 262, 265, 268, 276, 277, 288, 291, 292, 293, 294
module2	26, 33, 45, 58, 77, 78, 81, 86, 87, 89, 109, 141, 149, 169, 170, 174, 241, 285, 287, 323
module3	23, 30, 41, 45, 55, 77, 78, 81, 87, 89, 109, 112, 119, 161, 167, 197, 265, 283, 285
module4	26, 30, 41, 55, 58, 66, 77, 78, 81, 86, 87, 89, 90, 109, 112, 141, 144, 167, 169, 170, 174, 182, 201, 285, 323, 337
module5	26, 53, 66, 77, 78, 81, 89, 109, 116, 125, 137, 141, 144, 159, 167, 169, 170, 174, 209, 283, 287, 323, 343
module6	58, 75, 77, 90, 109, 144, 174, 182, 186, 187, 201, 250, 253, 333, 337
module7	53, 116, 125, 137, 159, 169, 186, 215, 216, 219, 300, 367
module8	26, 33, 45, 78, 86, 87, 137, 141, 170, 174, 287, 300, 323, 343
module9	150, 182, 187, 198, 201, 239, 241, 250, 253, 262, 294
module10	120, 130, 212, 215, 220, 225, 238, 241, 265, 268, 277, 288, 291, 292, 293, 294
module11	58, 75, 150, 197, 198, 239, 250, 253, 262
module12	16, 26, 33, 45, 53, 78, 137, 141, 159, 167, 169, 212, 283, 287, 300, 323, 343
module13	130, 215, 216, 220, 238, 277, 285
module14	53, 87, 109, 112, 116, 134, 137, 150, 159, 161, 167, 169, 212, 216, 238, 239, 283, 287, 291, 293
module15	26, 30, 66, 78, 89, 109, 112, 130, 159, 167, 169, 170, 212, 215, 219, 277, 287
module16	58, 78, 86, 116, 125, 141, 149, 337, 367
module17	144, 154, 167, 361
module18	78, 149
module19	55, 167
module20	182, 337
module21	58, 182
module22	55, 89
module23	55, 197
module24	357

Table S14. Methyl 3-residue flow for +BIRB796 p38γ

First Res	Second Res	Third Res	Scaled Flow
V16	V16	L30	527.7
V16	V16	V33	653.3
V16	V16	I55	384.8
V16	V16	L78	1749.4
V16	V16	M81	348.6
V16	V16	I87	106
V16	V16	L89	116.9
V16	V16	M109	150.7
V16	V16	V343	106
V16	V16	L367	353.4
V16	L30	L89	5135.1
V16	M81	V343	5646.3
V16	L89	L30	3299.8
V16	V343	M81	4068.4
V23	V23	V26	379.4
V23	V23	L30	911.5
V23	V23	V41	1139.5
V23	V23	I55	384.8
V23	V23	L66	571.9
V23	V23	L75	310.8
V23	V23	I87	1749.4
V23	V23	L89	982.6
V23	V23	L90	95.9
V23	V23	M112	141.8
V23	V23	L174	348.6
V23	L66	L89	5280.4
V23	L66	L90	6800.3
V23	L89	L66	6559.7
V23	L90	L66	4046.2
V26	V26	V23	379.4
V26	V26	L30	1749.4
V26	V26	L66	448.2
V26	V26	L75	706.6
V26	V26	V86	155.8
V26	V26	I87	412.6
V26	V26	L89	1874.9
V26	V26	L174	670.1
V26	V26	V361	240.6
V26	L66	L89	4508.6
V26	L89	L66	8512.1
L30	L30	V16	527.7
L30	L30	V23	911.5
L30	L30	V26	1749.4
L30	L30	V33	181.8
L30	L30	L66	982.6
L30	L30	I87	844.9
L30	L30	L89	1416
L30	L30	L90	724.5
L30	L30	V361	301
L30	L66	L89	5492.2
L30	L66	L90	5936.1
L30	L89	L66	6545.1
L30	L90	L66	5226.4
V33	V33	V16	653.3
V33	V33	L30	181.8
V33	V33	L337	97.3
V41	V41	V23	1139.5
V41	V41	V53	187.7
V41	V41	I55	324.5
V41	V41	L77	293.5
V41	V41	L78	141.8
V41	V41	I87	1139.5
V41	V41	M112	527.7
V41	V41	I149	249.7
V41	V41	L174	724.5
V41	V53	M112	4442.5
V41	L77	I149	4881.9
V41	L77	L174	3971.3
V41	M112	V53	6114.9
V41	I149	L77	4670.2
V41	L174	L77	5415.3
V45	V45	L78	225.1
V45	V45	L90	225.1
V53	V53	V41	187.7
V53	V53	I87	205.7
V53	V53	M109	2332.2
V53	V53	M112	3633.1
V53	V53	L116	782.7

First Res	Second Res	Third Res	Scaled Flow
V53	V53	L154	1749.4
V53	V53	V161	348.6
V53	V53	L170	3633.1
I55	V23	I87	4928.6
I55	I55	V16	384.8
I55	I55	V23	384.8
I55	I55	V41	324.5
I55	I55	L66	218.2
I55	I55	L78	401.3
I55	I55	I87	143.8
I55	I55	V333	130.6
I55	I87	V23	3713.8
L58	L58	L66	205.7
L58	L58	L77	246.2
L58	L58	L78	348.6
L58	L58	M81	571.9
L58	L58	V86	486.5
L58	L58	L89	412.6
L58	L66	L89	4912.4
L58	L89	L66	6061.3
L66	L66	V23	571.9
L66	L66	V26	448.2
L66	L66	L30	982.6
L66	L66	I55	218.2
L66	L66	L58	205.7
L66	L66	L89	4665.1
L66	L66	L90	3870.3
L66	L66	L174	106
L75	L75	V23	310.8
L75	L75	V26	706.6
L75	L75	L174	150.7
L75	L75	V186	612.1
L77	L77	V41	293.5
L77	L77	L58	246.2
L77	L77	V86	187.7
L77	L77	I149	2754.3
L77	L77	L174	2149.7
L77	L77	V333	412.6
L78	L78	V16	1749.4
L78	L78	V41	141.8
L78	L78	V45	225.1

First Res	Second Res	Third Res	Scaled Flow
L78	L78	I55	401.3
L78	L78	L58	348.6
L78	L78	M81	670.1
L78	L78	L89	187.7
L78	L78	M109	124.5
L78	L78	V343	293.5
L78	M81	V343	5826.3
L78	V343	M81	4404.5
M81	M81	V16	348.6
M81	M81	L58	571.9
M81	M81	L78	670.1
M81	M81	V343	2996.1
V86	V26	L89	4323.2
V86	L77	L174	4148.7
V86	V86	V26	155.8
V86	V86	L58	486.5
V86	V86	L77	187.7
V86	V86	L89	116.9
V86	V86	L174	320
V86	L89	V26	4018.4
V86	L174	L77	4840.7
I87	V26	L89	4979.5
I87	V53	M112	5019
I87	I87	V16	106
I87	I87	V23	1749.4
I87	I87	V26	412.6
I87	I87	L30	844.9
I87	I87	V41	1139.5
I87	I87	V53	205.7
I87	I87	I55	143.8
I87	I87	L89	171.1
I87	I87	M112	187.7
I87	I87	V161	106
I87	I87	V361	106.8
I87	L89	V26	3838.9
I87	M112	V53	4894.5
L89	V16	L78	3890.1
L89	V23	I87	6021.8
L89	L66	L90	12887.1
L89	L78	V16	4398.1
L89	I87	V23	3337.1

First Res	Second Res	Third Res	Scaled Flow
L89	L89	V16	116.9
L89	L89	V23	982.6
L89	L89	V26	1874.9
L89	L89	L30	1416
L89	L89	L58	412.6
L89	L89	L66	4665.1
L89	L89	L78	187.7
L89	L89	V86	116.9
L89	L89	I87	171.1
L89	L89	L90	619.3
L89	L89	L174	155.8
L89	L90	L66	3467.4
L90	L66	L89	11847.7
L90	L89	L66	3971.9
L90	L90	V23	95.9
L90	L90	L30	724.5
L90	L90	V45	225.1
L90	L90	L66	3870.3
L90	L90	L89	619.3
M109	V16	L78	4210.8
M109	V53	L170	6922.9
M109	L78	V16	4009.9
M109	M109	V16	150.7
M109	M109	V53	2332.2
M109	M109	L78	124.5
M109	M109	M112	2332.2
M109	M109	L116	454.5
M109	M109	L154	272.7
M109	M109	L170	1034.3
M109	M109	V343	208.6
M109	M112	L170	6922.9
M109	L154	L170	4382
M109	L170	V53	4407.2
M109	L170	M112	4407.2
M109	L170	L154	7098.5
M112	V23	I87	4007.5
M112	I87	V23	4315.9
M112	M112	V23	141.8
M112	M112	V41	527.7
M112	M112	V53	3633.1
M112	M112	I87	187.7

First Res	Second Res	Third Res	Scaled Flow
M112	M112	M109	2332.2
M112	M112	L154	527.7
M112	M112	L170	3633.1
M112	L154	L170	3697.6
M112	L170	L154	10957.6
L116	V53	M109	5231.3
L116	V53	L154	6055.7
L116	V53	L170	6458.1
L116	M109	V53	4292.6
L116	L116	V53	782.7
L116	L116	M109	454.5
L116	L116	I144	527.7
L116	L116	L154	106
L116	L116	L159	793.7
L116	L116	V161	293.5
L116	L116	L170	268.9
L116	L154	V53	3271.4
L116	L154	L170	4400.8
L116	L170	V53	4473.6
L116	L170	L154	5643
M120	M120	M219	679.5
M120	M120	L283	130.6
L125	L125	I215	338.8
L125	L125	L283	284.9
I130	I130	I220	793.7
I130	I130	V300	1520.3
I130	I130	V361	173.9
V134	V134	M291	571.9
M137	M137	I209	763.7
M137	M137	V212	619.3
M137	M137	I215	246.2
M137	M137	M219	246.2
M137	M137	L283	844.9
M137	M137	M291	2627.8
M137	M137	V293	106
M137	I209	I215	5509.5
M137	V212	V293	6878.2
M137	I215	I209	3743.1
M137	V293	V212	4070.4
L141	L141	I150	205.7
L141	L141	I197	1225.9

First Res	Second Res	Third Res	Scaled Flow
L141	L141	L198	1749.4
L141	L141	M201	724.5
L141	L141	I209	310.8
L141	L141	I215	225.1
L141	L141	M239	141.8
L141	L141	V250	116.9
L141	L141	M262	659.4
L141	L141	L268	268.9
L141	L141	L283	106
L141	L141	M291	448.2
L141	L141	L294	171.1
L141	I150	L268	5125.5
L141	L198	V250	7968.5
L141	M201	V250	6378.6
L141	I209	I215	4619.3
L141	I215	I209	4186.4
L141	I215	L283	4517.3
L141	M239	M262	4532
L141	M239	L294	4483.3
L141	V250	L198	2990.6
L141	V250	M201	3640.7
L141	M262	M239	7556.6
L141	M262	L268	5266.9
L141	L268	I150	5534.2
L141	L268	M262	3766.5
L141	L283	I215	3708.7
L141	L294	M239	4709.6
I144	I144	L116	527.7
I144	I144	L159	150.7
I144	I144	I169	95.9
I149	I149	V41	249.7
I149	I149	L77	2754.3
I149	I149	L174	436.1
I149	I149	V333	199.3
I150	L141	L198	3447.2
I150	I150	L141	205.7
I150	I150	I197	448.2
I150	I150	L198	911.5
I150	I150	M201	527.7
I150	I150	V212	448.2
I150	I150	V250	619.3
I150	I150	M262	248.1
I150	I150	L265	724.5
I150	I150	L268	4665.1
I150	I150	V293	205.7
I150	I197	L198	4050.3
I150	L198	L141	5726.2
I150	L198	I197	5303.8
I150	L198	M201	5248.3
I150	M201	L198	4241.1
I150	M201	V250	4655.9
I150	V212	V293	5913.8
I150	V250	M201	4935.4
I150	M262	L268	2539.2
I150	L268	M262	12287.7
I150	V293	V212	4662
L154	V53	M109	7293.9
L154	V53	M112	7384
L154	M109	V53	3452.9
L154	M109	M112	4220.2
L154	M112	V53	4312.6
L154	M112	M109	5206.6
L154	M112	L170	3540.2
L154	L154	V53	1749.4
L154	L154	M109	272.7
L154	L154	M112	527.7
L154	L154	L116	106
L154	L154	L170	3870.3
L154	L170	M112	11193.9
L159	L159	L116	793.7
L159	L159	I144	150.7
L159	L159	V161	228.3
V161	V161	V53	348.6
V161	V161	I87	106
V161	V161	L116	293.5
V161	V161	L159	228.3
V161	V161	L167	106
L167	L167	V161	106
L167	L167	I215	448.2
I169	I169	I144	95.9
L170	L170	V53	3633.1
L170	L170	M109	1034.3

First Res	Second Res	Third Res	Scaled Flow
L170	L170	M112	3633.1
L170	L170	L116	268.9
L170	L170	L154	3870.3
L174	V26	L89	5622.5
L174	L66	L89	4774
L174	L77	I149	7572
L174	L89	V26	3559.1
L174	L89	L66	5261.3
L174	I149	L77	3657.9
L174	L174	V23	348.6
L174	L174	V26	670.1
L174	L174	V41	724.5
L174	L174	L66	106
L174	L174	L75	150.7
L174	L174	L77	2149.7
L174	L174	V86	320
L174	L174	L89	155.8
L174	L174	I149	436.1
L174	L174	V186	124.5
M182	M182	V187	494.7
M182	M182	V323	96.5
V186	V186	L75	612.1
V186	V186	L174	124.5
V186	V186	V187	996.5
V186	V186	V300	97.3
V187	V187	M182	494.7
V187	V187	V186	996.5
I197	I150	L268	5879.4
I197	I197	L141	1225.9
I197	I197	I150	448.2
I197	I197	L198	2149.7
I197	I197	M201	348.6
I197	I197	I209	1488
I197	I197	V212	268.9
I197	I197	I215	527.7
I197	I197	I238	95.9
I197	I197	M239	116.9
I197	I197	V250	379.4
I197	I197	M262	369
I197	I197	L265	225.1
I197	I197	L268	320

First Res	Second Res	Third Res	Scaled Flow
I197	I197	M291	106
I197	I197	V293	670.1
I197	L198	M201	7493
I197	M201	L198	3353.8
I197	M201	V250	4658.5
I197	V212	V293	4643.6
I197	I238	M239	4391.8
I197	I238	M262	3244.2
I197	I238	V293	3128.3
I197	M239	I238	4607.9
I197	M239	M262	4640.5
I197	V250	M201	4786
I197	M262	I238	4865.6
I197	M262	M239	6633.4
I197	M262	L268	4570.6
I197	L268	I150	5273.9
I197	L268	M262	4217.9
I197	V293	V212	6304.9
I197	V293	I238	5578.5
L198	I150	L268	6172.7
L198	I197	I209	7884.4
L198	L198	L141	1749.4
L198	L198	I150	911.5
L198	L198	I197	2149.7
L198	L198	M201	2299.8
L198	L198	I209	137.1
L198	L198	V212	1225.9
L198	L198	I215	106
L198	L198	M216	187.7
L198	L198	I238	911.5
L198	L198	M239	1058.5
L198	L198	V241	187.7
L198	L198	V250	1874.9
L198	L198	M262	974.2
L198	L198	L265	571.9
L198	L198	L268	724.5
L198	L198	I276	348.6
L198	L198	M291	187.7
L198	L198	L292	106
L198	L198	V293	1225.9
L198	L198	L294	670.1

First Res	Second Res	Third Res	Scaled Flow
L198	I209	I197	2720.4
L198	I209	I215	4318.1
L198	V212	V293	5584.3
L198	I215	I209	4036.2
L198	M216	I276	3904.9
L198	M216	L292	5384.8
L198	I238	M239	4940.9
L198	I238	V241	6731.9
L198	I238	I276	7630.6
L198	M239	I238	5279.4
L198	M239	V241	8272.9
L198	M239	M262	6355.7
L198	M239	L294	5527.9
L198	V241	I238	3952.2
L198	V241	M239	4545.5
L198	V241	I276	4040
L198	M262	M239	6345
L198	L268	I150	5615.8
L198	I276	M216	4679.5
L198	I276	I238	5368.3
L198	I276	V241	4841.3
L198	I276	L292	4755.9
L198	L292	M216	4649.6
L198	L292	I276	3426.8
L198	V293	V212	5584.3
L198	L294	M239	4565.9
M201	I150	L268	5907.5
M201	I197	L198	3219.6
M201	L198	I197	7527.6
M201	M201	L141	724.5
M201	M201	I150	527.7
M201	M201	I197	348.6
M201	M201	L198	2299.8
M201	M201	M239	187.7
M201	M201	V250	2459
M201	M201	L253	2149.7
M201	M201	M262	248.1
M201	M201	L265	782.7
M201	M201	L268	412.6
M201	M201	L294	844.9
M201	M239	M262	5211.7

First Res	Second Res	Third Res	Scaled Flow
M201	M239	L294	3925.7
M201	M262	M239	5840.3
M201	M262	L268	4131.9
M201	L268	I150	5430.2
M201	L268	M262	4665.2
M201	L294	M239	6475.5
I209	M137	M291	5015.2
I209	L141	L198	4749.1
I209	I197	L198	7574.7
I209	L198	L141	3780.4
I209	L198	I197	3238.3
I209	L198	V212	3852.1
I209	I209	M137	763.7
I209	I209	L141	310.8
I209	I209	I197	1488
I209	I209	L198	137.1
I209	I209	V212	118.6
I209	I209	I215	1967.9
I209	I209	M262	158
I209	I209	L283	384.8
I209	I209	M291	679.5
I209	I209	V293	261
I209	V212	L198	3714.1
I209	V212	V293	4491.8
I209	M291	M137	4789.7
I209	V293	V212	5553.9
V212	M137	M291	4337.8
V212	I150	L268	4925.8
V212	I197	L198	3589.7
V212	I197	I209	4511
V212	L198	I197	6324.1
V212	I209	I197	3628.9
V212	V212	M137	619.3
V212	V212	I150	448.2
V212	V212	I197	268.9
V212	V212	L198	1225.9
V212	V212	I209	118.6
V212	V212	I238	982.6
V212	V212	M239	619.3
V212	V212	V241	571.9
V212	V212	M262	1012

First Res	Second Res	Third Res	Scaled Flow
V212	V212	L265	95.9
V212	V212	L268	1139.5
V212	V212	I276	1058.5
V212	V212	M291	1139.5
V212	V212	L292	106
V212	V212	V293	3870.3
V212	I238	M239	5638.5
V212	I238	V241	5727.5
V212	I238	I276	6561.3
V212	M239	I238	4674.2
V212	M239	V241	6147
V212	M239	M262	5567
V212	V241	I238	4610.5
V212	V241	M239	5969.1
V212	M262	M239	7050.7
V212	L268	I150	7132.2
V212	I276	I238	6784.7
V212	I276	L292	6248.3
V212	M291	M137	5599.4
V212	L292	I276	2964.3
I215	M137	M291	4965.6
I215	L141	L198	4588.3
I215	I197	L198	5757.3
I215	L198	L141	3767
I215	L198	I197	3611.6
I215	I215	L125	338.8
I215	I215	M137	246.2
I215	I215	L141	225.1
I215	I215	L167	448.2
I215	I215	I197	527.7
I215	I215	L198	106
I215	I215	I209	1967.9
I215	I215	M216	246.2
I215	I215	M239	106
I215	I215	M262	353.4
I215	I215	L277	246.2
I215	I215	L283	1631.4
I215	I215	M291	141.8
I215	I215	L292	571.9
I215	M216	L277	5929.3
I215	M216	L292	4814.7

First Res	Second Res	Third Res	Scaled Flow
I215	M239	M262	4584.5
I215	M262	M239	6621.6
I215	L277	M216	5929.3
I215	L277	L292	4523.2
I215	M291	M137	4273.2
I215	L292	M216	6322.4
I215	L292	L277	5939.6
M216	I215	L283	4088.4
M216	M216	L198	187.7
M216	M216	I215	246.2
M216	M216	I238	1225.9
M216	M216	M239	141.8
M216	M216	V241	95.9
M216	M216	I276	1749.4
M216	M216	L277	7522.2
M216	M216	L283	293.5
M216	M216	L288	246.2
M216	M216	L292	4385.6
M216	M216	V293	187.7
M216	I238	M239	7692.3
M216	I238	V241	8097.7
M216	I238	I276	6263.3
M216	M239	I238	3661.8
M216	M239	V241	5394.3
M216	V241	I238	3499.8
M216	V241	M239	4897.5
M216	V241	I276	2998.9
M216	V241	L288	3510.6
M216	I276	I238	7567.7
M216	I276	V241	8383.7
M216	L283	I215	4306.2
M216	L288	V241	4493.1
M219	M137	M291	4919.7
M219	M219	M120	679.5
M219	M219	M137	246.2
M219	M219	L283	571.9
M219	M219	V285	535.2
M219	M219	M291	155.8
M219	M291	M137	4338.9
I220	I220	I130	793.7
I220	I220	V300	284.9

First Res	Second Res	Third Res	Scaled Flow
I220	I220	V361	238.5
L225	L225	I238	246.2
L225	L225	M239	448.2
L225	L225	V241	1058.5
L225	L225	I276	225.1
L225	L225	L288	1520.3
L225	L225	L294	2299.8
L225	L225	L357	250.8
L225	I238	M239	4515
L225	I238	V241	4010.5
L225	I238	I276	5829.4
L225	M239	I238	5443.2
L225	M239	V241	5249
L225	M239	L294	3626.9
L225	V241	I238	6765
L225	V241	M239	7344.4
L225	V241	I276	6120.2
L225	I276	I238	5682.3
L225	I276	V241	3536.6
L225	L294	M239	7812.2
I238	I197	L198	3342.5
I238	L198	I197	6753.6
I238	V212	V293	5135.5
I238	M216	L277	8452.4
I238	M216	L292	6759.9
I238	L225	L294	4407
I238	I238	I197	95.9
I238	I238	L198	911.5
I238	I238	V212	982.6
I238	I238	M216	1225.9
I238	I238	L225	246.2
I238	I238	M239	2996.1
I238	I238	V241	2996.1
I238	I238	M262	1313.9
I238	I238	L268	95.9
I238	I238	I276	6693
I238	I238	L277	412.6
I238	I238	L288	782.7
I238	I238	L292	619.3
I238	I238	V293	1416
I238	I238	L294	320

First Res	Second Res	Third Res	Scaled Flow
I238	M239	V241	6289.4
I238	M239	M262	8611.1
I238	M239	L294	9712.1
I238	V241	M239	6289.4
I238	M262	M239	5262.4
I238	M262	L268	7491.5
I238	L268	M262	3019.2
I238	L277	M216	5482.7
I238	L277	L292	4888.1
I238	L292	M216	5056.7
I238	L292	L277	5637
I238	V293	V212	6120.1
I238	L294	L225	4765.7
I238	L294	M239	3310.7
M239	L141	I197	3878.1
M239	L141	L198	3233.8
M239	I197	L141	3694.2
M239	I197	L198	3349.9
M239	L198	L141	6338.6
M239	L198	I197	6893.1
M239	L198	M201	6551.8
M239	M201	L198	3599.8
M239	V212	V293	5068.4
M239	M216	I276	3123.2
M239	M216	L292	4922.4
M239	L225	L294	3184.5
M239	I238	I276	9369.4
M239	M239	L141	141.8
M239	M239	I197	116.9
M239	M239	L198	1058.5
M239	M239	M201	187.7
M239	M239	V212	619.3
M239	M239	I215	106
M239	M239	M216	141.8
M239	M239	L225	448.2
M239	M239	I238	2996.1
M239	M239	V241	5599.2
M239	M239	M262	5718.9
M239	M239	L265	293.5
M239	M239	L268	448.2
M239	M239	I276	1317.9

First Res	Second Res	Third Res	Scaled Flow
M239	M239	L288	571.9
M239	M239	L292	171.1
M239	M239	V293	982.6
M239	M239	L294	2806.7
M239	M239	L357	172.1
M239	M262	L268	13029.4
M239	L268	M262	2208.8
M239	I276	M216	6800.6
M239	I276	I238	5534.2
M239	L292	M216	5170.8
M239	V293	V212	6114
M239	L294	L225	7945.8
V241	V212	V293	4859.4
V241	M216	I238	2345.4
V241	M216	I276	2821.3
V241	M216	L292	4765.3
V241	I238	M216	9523
V241	I238	I276	8176.2
V241	M239	M262	13602.3
V241	V241	L198	187.7
V241	V241	V212	571.9
V241	V241	M216	95.9
V241	V241	L225	1058.5
V241	V241	I238	2996.1
V241	V241	M239	5599.2
V241	V241	M262	1465.7
V241	V241	L265	141.8
V241	V241	L268	116.9
V241	V241	I276	2008.2
V241	V241	L288	1416
V241	V241	L292	106
V241	V241	V293	1139.5
V241	V241	L294	1139.5
V241	V241	L357	764.3
V241	M262	M239	4220.1
V241	M262	L268	7570.4
V241	L268	M262	3025.3
V241	I276	M216	8575.2
V241	I276	I238	6120.6
V241	I276	L292	7914.3
V241	L292	M216	4880.8

First Res	Second Res	Third Res	Scaled Flow
V241	L292	I276	2667
V241	V293	V212	6459.6
V250	L141	L198	2896.6
V250	I150	L268	6965.1
V250	I197	L198	3404.3
V250	L198	L141	8042.3
V250	L198	I197	6790.6
V250	M201	L253	7593.9
V250	V250	L141	116.9
V250	V250	I150	619.3
V250	V250	I197	379.4
V250	V250	L198	1874.9
V250	V250	M201	2459
V250	V250	L253	379.4
V250	V250	L265	1520.3
V250	V250	L268	141.8
V250	V250	V293	141.8
V250	V250	L294	155.8
V250	V250	L367	475.7
V250	L253	M201	3183.3
V250	L268	I150	4432.5
L253	M201	V250	7524.6
L253	V250	M201	3460.1
L253	V250	L265	4756.3
L253	L253	M201	2149.7
L253	L253	V250	379.4
L253	L253	L265	141.8
L253	L253	L294	782.7
L253	L265	V250	3587.3
M262	I150	L268	4155.8
M262	I197	L198	3893.3
M262	I197	I209	4634.2
M262	L198	I197	5594.9
M262	L198	M201	6118.9
M262	M201	L198	3773.8
M262	I209	I197	3640.1
M262	I209	I215	3918.7
M262	V212	V293	6365.5
M262	I215	I209	4920.8
M262	I238	I276	7774.1
M262	M239	V241	14056.8

First Res	Second Res	Third Res	Scaled Flow
M262	M239	L294	11403.9
M262	V241	M239	4054.9
M262	M262	L141	659.4
M262	M262	I150	248.1
M262	M262	I197	369
M262	M262	L198	974.2
M262	M262	M201	248.1
M262	M262	I209	158
M262	M262	V212	1012
M262	M262	I215	353.4
M262	M262	I238	1313.9
M262	M262	M239	5718.9
M262	M262	V241	1465.7
M262	M262	L265	95.1
M262	M262	L268	1881
M262	M262	I276	659.4
M262	M262	M291	95.1
M262	M262	L292	128.4
M262	M262	V293	516.8
M262	M262	L294	937.7
M262	L268	I150	9455.6
M262	I276	I238	5753.9
M262	V293	V212	4877
M262	L294	M239	2655.7
L265	I150	L268	6897.6
L265	I197	L198	3961.4
L265	L198	I197	5336.6
L265	L198	M201	4428.4
L265	M201	L198	4995.7
L265	M201	L253	6144
L265	V212	V293	4173.6
L265	M239	V241	6061
L265	M239	M262	6179.1
L265	M239	L294	5068.5
L265	V241	M239	4952.2
L265	V241	M262	4038.4
L265	L253	M201	3569.3
L265	M262	M239	4739.6
L265	M262	V241	3791.1
L265	M262	L268	3835.6
L265	L265	I150	724.5

First Res	Second Res	Third Res	Scaled Flow
L265	L265	I197	225.1
L265	L265	L198	571.9
L265	L265	M201	782.7
L265	L265	V212	95.9
L265	L265	M239	293.5
L265	L265	V241	141.8
L265	L265	V250	1520.3
L265	L265	L253	141.8
L265	L265	M262	95.1
L265	L265	L268	225.1
L265	L265	V293	412.6
L265	L265	L294	187.7
L265	L265	L357	228.7
L265	L268	I150	4681.2
L265	L268	M262	4627.8
L265	V293	V212	6263.9
L265	L294	M239	4460
L268	I197	L198	4020.6
L268	L198	I197	5339.9
L268	L198	M201	5218.4
L268	L198	V250	5809.1
L268	M201	L198	4260.6
L268	M201	V250	5436.1
L268	V212	V293	7429.3
L268	I238	M239	3914
L268	I238	V241	4391.8
L268	I238	M262	2575.2
L268	I238	V293	3510.6
L268	M239	I238	6039.2
L268	M239	V241	6798.6
L268	M239	M262	4715.7
L268	M239	L294	5712.9
L268	V241	I238	4607.9
L268	V241	M239	4622.9
L268	V241	M262	2696.3
L268	V250	L198	3480.7
L268	V250	M201	3989.5
L268	V250	L265	3808.3
L268	M262	I238	7788.8
L268	M262	M239	9243.7
L268	M262	V241	7772.5

First Res	Second Res	Third Res	Scaled Flow
L268	L265	V250	4313.6
L268	L268	L141	268.9
L268	L268	I150	4665.1
L268	L268	I197	320
L268	L268	L198	724.5
L268	L268	M201	412.6
L268	L268	V212	1139.5
L268	L268	I238	95.9
L268	L268	M239	448.2
L268	L268	V241	116.9
L268	L268	V250	141.8
L268	L268	M262	1881
L268	L268	L265	225.1
L268	L268	V293	246.2
L268	L268	L294	141.8
L268	V293	V212	4256.1
L268	V293	I238	4493.1
L268	L294	M239	4078.1
I276	V212	V293	6235.4
I276	M216	L277	8654.2
I276	M216	L292	6197.3
I276	L225	L294	4563
I276	M239	V241	5760.6
I276	M239	M262	7289.5
I276	M239	L294	7332.5
I276	V241	M239	7300.7
I276	M262	M239	5582.1
I276	I276	L198	348.6
I276	I276	V212	1058.5
I276	I276	M216	1749.4
I276	I276	L225	225.1
I276	I276	I238	6693
I276	I276	M239	1317.9
I276	I276	V241	2008.2
I276	I276	M262	659.4
I276	I276	L277	782.7
I276	I276	L288	320
I276	I276	M291	128.8
I276	I276	L292	1416
I276	I276	V293	619.3
I276	I276	L294	205.7

First Res	Second Res	Third Res	Scaled Flow
I276	L277	M216	5869.2
I276	L277	L292	4701.3
I276	L292	M216	5515.7
I276	L292	L277	6169.6
I276	V293	V212	4998.8
I276	L294	L225	4448.9
I276	L294	M239	3718.9
L277	I215	L283	4088.4
L277	I238	I276	5656.1
L277	I276	I238	7145.1
L277	L277	I215	246.2
L277	L277	M216	7522.2
L277	L277	I238	412.6
L277	L277	I276	782.7
L277	L277	L283	293.5
L277	L277	L288	225.1
L277	L277	L292	3408.7
L277	L283	I215	4306.2
L283	M137	M291	5382.3
L283	M216	L277	6049.8
L283	L277	M216	6049.8
L283	L283	M120	130.6
L283	L283	L125	284.9
L283	L283	M137	844.9
L283	L283	L141	106
L283	L283	I209	384.8
L283	L283	I215	1631.4
L283	L283	M216	293.5
L283	L283	M219	571.9
L283	L283	L277	293.5
L283	L283	M291	527.7
L283	M291	M137	4491.4
V285	V285	M219	535.2
L288	M216	I276	4115.4
L288	M216	L277	5975.2
L288	L225	L294	6928.7
L288	I238	M239	5427.6
L288	I238	V241	4485.3
L288	I238	I276	7380.7
L288	M239	I238	4811.2
L288	M239	V241	5232.9

First Res	Second Res	Third Res	Scaled Flow
L288	M239	L294	5585.6
L288	V241	I238	5886.3
L288	V241	M239	7747
L288	V241	I276	6256.8
L288	I276	M216	4450.5
L288	I276	I238	5388
L288	I276	V241	3480.5
L288	L277	M216	5824.4
L288	L288	M216	246.2
L288	L288	L225	1520.3
L288	L288	I238	782.7
L288	L288	M239	571.9
L288	L288	V241	1416
L288	L288	I276	320
L288	L288	L277	225.1
L288	L288	V293	246.2
L288	L288	L294	268.9
L288	L288	V300	95.9
L288	L294	L225	3521.8
L288	L294	M239	4364.8
M291	L141	L198	4919.1
M291	I197	L198	4008
M291	I197	I209	3191.7
M291	L198	L141	3781.7
M291	L198	I197	4641.8
M291	I209	I197	5606.5
M291	I209	I215	5797
M291	V212	V293	7041.4
M291	I215	I209	3548.9
M291	I215	L283	3502.6
M291	L283	I215	5192.3
M291	M291	V134	571.9
M291	M291	M137	2627.8
M291	M291	L141	448.2
M291	M291	I197	106
M291	M291	L198	187.7
M291	M291	I209	679.5
M291	M291	V212	1139.5
M291	M291	I215	141.8
M291	M291	M219	155.8
M291	M291	M262	95.1

First Res	Second Res	Third Res	Scaled Flow
M291	M291	I276	128.8
M291	M291	L283	527.7
M291	M291	V293	348.6
M291	V293	V212	4480.2
L292	L198	V212	3739.7
L292	L198	V293	3607.5
L292	V212	L198	3739.7
L292	V212	V293	4599
L292	M216	L277	8549.6
L292	I238	M239	6136
L292	I238	V241	6484
L292	I238	I276	5607.3
L292	M239	I238	4101.9
L292	M239	V241	5553.3
L292	M239	M262	5401.7
L292	V241	I238	3837.1
L292	V241	M239	4915.9
L292	V241	M262	3752.3
L292	V241	I276	3123.8
L292	M262	M239	5195.5
L292	M262	V241	4077
L292	I276	I238	8061
L292	I276	V241	7588.4
L292	L277	M216	6539.6
L292	L292	L198	106
L292	L292	V212	106
L292	L292	I215	571.9
L292	L292	M216	4385.6
L292	L292	I238	619.3
L292	L292	M239	171.1
L292	L292	V241	106
L292	L292	M262	128.4
L292	L292	I276	1416
L292	L292	L277	3408.7
L292	L292	V293	155.8
L292	V293	L198	3975.7
L292	V293	V212	5068.4
V293	M137	M291	3951.7
V293	I150	L268	5168.2
V293	L198	V250	6770
V293	M216	I276	3617.3

First Res	Second Res	Third Res	Scaled Flow
V293	M216	L292	5220.5
V293	I238	M239	5553.4
V293	I238	V241	5331.4
V293	I238	I276	8061
V293	M239	I238	4659.9
V293	M239	V241	6115.7
V293	M239	M262	6945.4
V293	V241	I238	4787.1
V293	V241	M239	6544.1
V293	V250	L198	3222.8
V293	V250	L265	3550.4
V293	M262	M239	5586.9
V293	M262	L268	5063.9
V293	L265	V250	4837.9
V293	L268	I150	5438
V293	L268	M262	3859.1
V293	I276	M216	5277.9
V293	I276	I238	5607.3
V293	M291	M137	5484.4
V293	L292	M216	4967.8
V293	V293	M137	106
V293	V293	I150	205.7
V293	V293	I197	670.1
V293	V293	L198	1225.9
V293	V293	I209	261
V293	V293	V212	3870.3
V293	V293	M216	187.7
V293	V293	I238	1416
V293	V293	M239	982.6
V293	V293	V241	1139.5
V293	V293	V250	141.8
V293	V293	M262	516.8
V293	V293	L265	412.6
V293	V293	L268	246.2
V293	V293	I276	619.3
V293	V293	L288	246.2
V293	V293	M291	348.6
V293	V293	L292	155.8
L294	L141	L198	3535.2
L294	L198	L141	5448.5
L294	L198	V250	5622.5

First Res	Second Res	Third Res	Scaled Flow
L294	M201	V250	6416.3
L294	I238	M239	3440.8
L294	I238	V241	4107.1
L294	I238	I276	6208.1
L294	M239	I238	9570.9
L294	M239	V241	8770.6
L294	M239	M262	9173.8
L294	V241	I238	6629.5
L294	V241	M239	5089.6
L294	V241	I276	6351.1
L294	V250	L198	3559.1
L294	V250	M201	3701.6
L294	V250	L265	3939.3
L294	M262	M239	5050.6
L294	M262	L268	6350.3
L294	L265	V250	4139.7
L294	L268	M262	3300
L294	I276	I238	5455.9
L294	I276	V241	3457.9
L294	L294	L141	171.1
L294	L294	L198	670.1
L294	L294	M201	844.9
L294	L294	L225	2299.8
L294	L294	I238	320
L294	L294	M239	2806.7
L294	L294	V241	1139.5
L294	L294	V250	155.8
L294	L294	L253	782.7
L294	L294	M262	937.7
L294	L294	L265	187.7
L294	L294	L268	141.8
L294	L294	I276	205.7
L294	L294	L288	268.9
V300	V300	I130	1520.3
V300	V300	V186	97.3
V300	V300	I220	284.9
V300	V300	L288	95.9
V300	V300	V333	128.8
V323	V323	M182	96.5
V333	L77	I149	5363.5
V333	I149	L77	4324.3

First Res	Second Res	Third Res	Scaled Flow
V333	V333	I55	130.6
V333	V333	L77	412.6
V333	V333	I149	199.3
V333	V333	V300	128.8
L337	L337	V33	97.3
V343	V16	L78	3667.9
V343	L78	V16	4827.5
V343	V343	V16	106
V343	V343	L78	293.5
V343	V343	M81	2996.1
V343	V343	M109	208.6
L357	M239	V241	4658.5
L357	V241	M239	7548.6
L357	L357	L225	250.8
L357	L357	M239	172.1
L357	L357	V241	764.3
L357	L357	L265	228.7
V361	V26	L30	4132.6
V361	L30	V26	4415.4
V361	V361	V26	240.6
V361	V361	L30	301
V361	V361	I87	106.8
V361	V361	I130	173.9
V361	V361	I220	238.5
V361	V361	L367	126.2
L367	L367	V16	353.4
L367	L367	V250	475.7
L367	L367	V361	126.2

Table S15. Community membership for +BIRB796 p38γ

module1	141, 150, 197, 198, 201, 209, 212, 215, 216, 238, 239, 241, 250, 262, 265, 268, 276, 291, 292, 293, 294, 357
module2	120, 125, 137, 141, 150, 161, 167, 197, 198, 201, 209, 212, 215, 216, 219, 238, 239, 241, 250, 253, 262, 265, 268, 276, 277, 283, 291, 292, 293, 294, 357, 367
module3	198, 215, 216, 238, 239, 241, 262, 276, 277, 283, 288, 292, 293
module4	197, 198, 212, 216, 225, 238, 239, 241, 262, 276, 277, 288, 291, 292, 293, 294
module5	16, 23, 41, 53, 78, 87, 109, 112, 116, 144, 154, 159, 161, 167, 169, 170, 343
module6	16, 23, 26, 30, 33, 45, 55, 58, 66, 75, 78, 86, 87, 89, 90, 174, 361
module7	141, 198, 201, 225, 238, 239, 241, 250, 253, 262, 265, 268, 276, 288, 294, 357
module8	137, 150, 197, 198, 209, 212, 216, 238, 239, 241, 250, 262, 265, 268, 276, 288, 291, 292, 293
module9	134, 137, 141, 197, 198, 209, 212, 215, 219, 262, 276, 283, 291, 293
module10	23, 26, 41, 55, 58, 66, 75, 77, 86, 89, 149, 174, 186, 333
module11	16, 23, 26, 30, 41, 53, 55, 66, 75, 78, 87, 89, 90, 112, 161, 174, 333, 361
module12	16, 30, 33, 41, 45, 55, 58, 78, 81, 87, 89, 109, 337, 343, 367
module13	16, 58, 78, 81, 109, 343
module14	216, 225, 238, 239, 241, 276, 277, 288, 293, 294, 300
module15	130, 186, 220, 288, 300, 333, 361, 367
module16	75, 174, 182, 186, 187, 300, 323
module17	120, 137, 219, 283, 285, 291
module18	198, 239
module19	198, 238
module20	198, 276
module21	58, 66
module22	119
module23	239, 276
module24	239, 268
module25	241, 268
module26	287

References

1 Tugarinov, V., Kanelis, V. & Kay, L. E. Isotope labeling strategies for the study of high-molecular-weight proteins by solution NMR spectroscopy. *Nat. Protoc.* **1**, 749-754, doi:10.1038/nprot.2006.101 (2006).

2 Tugarinov, V. & Kay, L. Ile, Leu, and Val methyl assignments of the 723-residue malate synthase G using a new labeling strategy and novel NMR methods. *J. Am. Chem. Soc.* **125**, 13868-13878, doi:10.1021/ja030345s (2003).

3 Hyberts, S. G., Takeuchi, K. & Wagner, G. Poisson-gap sampling and forward maximum entropy reconstruction for enhancing the resolution and sensitivity of protein NMR data. *J. Am. Chem. Soc.* **132**, 2145-2147, doi:10.1021/ja908004w (2010).

4 Aoto, P. C., Fenwick, R. B., Kroon, G. J. A. & Wright, P. E. Accurate scoring of non-uniform sampling schemes for quantitative NMR. *Journal of magnetic resonance (San Diego, Calif : 1997)* **246**, 31-35, doi:10.1016/j.jmr.2014.06.020 (2014).

5 Otten, R., Chu, B., Krewulak, K. D., Vogel, H. J. & Mulder, F. A. A. Comprehensive and cost-effective NMR spectroscopy of methyl groups in large proteins. *J. Am. Chem. Soc.* **132**, 2952-2960, doi:10.1021/ja907706a (2010).

6 Neri, D., Szyperski, T., Otting, G., Senn, H. & Wüthrich, K. Stereospecific nuclear magnetic resonance assignments of the methyl groups of valine and leucine in the DNA-binding domain of the 434 repressor by biosynthetically directed fractional 13C labeling. *Biochemistry* **28**, 7510-7516 (1989).

7 Shi, Z., Resing, K. A. & Ahn, N. G. Networks for the allosteric control of protein kinases. *Curr. Opin. Struct. Biol.* **16**, 686-692, doi:10.1016/j.sbi.2006.10.011 (2006).

8 Huse, M. & Kuriyan, J. The conformational plasticity of protein kinases. *Cell* **109**, 275-282 (2002).

9 Diskin, R., Engelberg, D. & Livnah, O. A novel lipid binding site formed by the MAP kinase insert in p38 alpha. *J. Mol. Biol.* **375**, 70-79, doi:10.1016/j.jmb.2007.09.002 (2008).

10 Kannan, N. & Neuwald, A. F. Evolutionary constraints associated with functional specificity of the CMGC protein kinases MAPK, CDK, GSK, SRPK, DYRK, and CK2alpha. *Protein science : a publication of the Protein Society* **13**, 2059-2077, doi:10.1110/ps.04637904 (2004).

11 Tokunaga, Y., Takeuchi, K., Takahashi, H. & Shimada, I. Allosteric enhancement of MAP kinase p38α's activity and substrate selectivity by docking interactions. *Nat. Struct. Mol. Biol.*, doi:10.1038/nsmb.2861 (2014).

12 Masterson, L. R. *et al.* Dynamics connect substrate recognition to catalysis in protein kinase A. *Nat. Chem. Biol.*, 1-8, doi:10.1038/nchembio.452 (2010).

13 Srivastava, Atul K. *et al.* Synchronous Opening and Closing Motions Are Essential for cAMP-Dependent Protein Kinase A Signaling. *Structure/Folding and Design* **22**, 1735-1743, doi:10.1016/j.str.2014.09.010 (2014).
14 Taylor, S. S. & Kornev, A. P. Protein kinases: evolution of dynamic regulatory proteins. *Trends Biochem. Sci.* **36**, 65-77, doi:10.1016/j.tibs.2010.09.006 (2011).
15 Zhang, J. *et al.* Targeting Bcr-Abl by combining allosteric with ATP-binding-site inhibitors. *Nature* **463**, 501-506, doi:10.1038/nature08675 (2010).
16 Skora, L., Mestan, J., Fabbro, D., Jahnke, W. & Grzesiek, S. NMR reveals the allosteric opening and closing of Abelson tyrosine kinase by ATP-site and myristoyl pocket inhibitors. *Proc. Natl. Acad. Sci. U. S. A.*, doi:10.1073/pnas.1314712110 (2013).
17 Zhang, Y.-Y., Wu, J.-W. & Wang, Z.-X. Mitogen-activated protein kinase (MAPK) phosphatase 3-mediated cross-talk between MAPKs ERK2 and p38alpha. *J. Biol. Chem.* **286**, 16150-16162, doi:10.1074/jbc.M110.203786 (2011).
18 Diskin, R., Lebendiker, M., Engelberg, D. & Livnah, O. Structures of p38alpha active mutants reveal conformational changes in L16 loop that induce autophosphorylation and activation. *J. Mol. Biol.* **365**, 66-76, doi:10.1016/j.jmb.2006.08.043 (2007).
19 Salvador, J. M. *et al.* Alternative p38 activation pathway mediated by T cell receptor-proximal tyrosine kinases. *Nat. Immunol.* **6**, 390-395, doi:10.1038/ni1177 (2005).
20 Tzarum, N., Diskin, R., Engelberg, D. & Livnah, O. Active Mutants of the TCR-Mediated p38α Alternative Activation Site Show Changes in the Phosphorylation Lip and DEF Site Formation. *J. Mol. Biol.* **405**, 1154-1169, doi:10.1016/j.jmb.2010.11.023 (2011).
21 De Nicola, G. F. *et al.* Mechanism and consequence of the autoactivation of p38α mitogen-activated protein kinase promoted by TAB1. *Nat. Struct. Mol. Biol.*, doi:10.1038/nsmb.2668 (2013).
22 Ahn, Y.-Y., Bagrow, J. P. & Lehmann, S. Link communities reveal multiscale complexity in networks. *Nature* **466**, 761-764, doi:10.1038/nature09182 (2010).
23 Kalinka, A. T. & Tomancak, P. linkcomm: an R package for the generation, visualization, and analysis of link communities in networks of arbitrary size and type. *Bioinformatics* **27**, 2011-2012, doi:10.1093/bioinformatics/btr311 (2011).
24 cluster: Cluster Analysis Basics and Extensions v. R package version 2.0.3 (2015).
